# Genomic analyses of withers height and linear conformation traits in German Warmblood horses using imputed sequence-level genotypes

**DOI:** 10.1186/s12711-024-00914-6

**Published:** 2024-06-13

**Authors:** Paula Reich, Sandra Möller, Kathrin F. Stock, Wietje Nolte, Mario von Depka Prondzinski, Reinhard Reents, Ernst Kalm, Christa Kühn, Georg Thaller, Clemens Falker-Gieske, Jens Tetens

**Affiliations:** 1 Department of Animal Sciences, Georg-August-University Göttingen, 37077 Göttingen, Germany; 2 Center for Integrated Breeding Research (CiBreed), Georg-August-University Göttingen, 37075 Göttingen, Germany; 3 IT Solutions for Animal Production (vit), 27283 Verden, Germany; 4 Saxon State Office for Environment, Agriculture and Geology, 01468 Moritzburg, Germany; 5 Werlhof Institute, 30159 Hannover, Germany; 6 Institute of Animal Breeding and Husbandry, Kiel University, 24098 Kiel, Germany; 7 Institute of Genome Biology, Research Institute for Farm Animal Biology (FBN), 18196 Dummerstorf, Germany; 8 Faculty of Agricultural and Environmental Sciences, University of Rostock, 18059 Rostock, Germany; 9 Present Address: Friedrich-Loeffler-Institute, 17493 Greifswald - Riems Island, Germany

## Abstract

**Background:**

Body conformation, including withers height, is a major selection criterion in horse breeding and is associated with other important traits, such as health and performance. However, little is known about the genomic background of equine conformation. Therefore, the aim of this study was to use imputed sequence-level genotypes from up to 4891 German Warmblood horses to identify genomic regions associated with withers height and linear conformation traits. Furthermore, the traits were genetically characterised and putative causal variants for withers height were detected.

**Results:**

A genome-wide association study (GWAS) for withers height confirmed the presence of a previously known quantitative trait locus (QTL) on *Equus caballus* (ECA) chromosome 3 close to the *LCORL*/*NCAPG* locus, which explained 16% of the phenotypic variance for withers height. An additional significant association signal was detected on ECA1. Further investigations of the region on ECA3 identified a few promising candidate causal variants for withers height, including a nonsense mutation in the coding sequence of the *LCORL* gene. The estimated heritability for withers height was 0.53 and ranged from 0 to 0.34 for the conformation traits. GWAS identified significantly associated variants for more than half of the investigated conformation traits, among which 13 showed a peak on ECA3 in the same region as withers height. Genetic parameter estimation revealed high genetic correlations between these traits and withers height for the QTL on ECA3.

**Conclusions:**

The use of imputed sequence-level genotypes from a large study cohort led to the discovery of novel QTL associated with conformation traits in German Warmblood horses. The results indicate the high relevance of the QTL on ECA3 for various conformation traits, including withers height, and contribute to deciphering causal mutations for body size in horses.

**Supplementary Information:**

The online version contains supplementary material available at 10.1186/s12711-024-00914-6.

## Background

Conformation, along with health and performance, represents one of the major selection criteria in horse breeding [1]. Conformation not only affects the overall appearance of an individual, but it is also associated with locomotor health and sports performance [2–6]. Hence, assessing the conformation of young horses is a means to indirectly select for performance [1] prior to the participation of animals in riding competitions, resulting in shortened generation intervals and increased selection intensity [7]. Accordingly, deciphering the genetic background of morphological traits is of great interest to simultaneously improve conformation and correlated traits, such as health and performance. Knowledge of causal mutations underlying specific traits allows for the development of genetic tests and the consideration of the identified variants in breeding programmes.

A common method to identify genomic regions that are associated with traits of interest is to perform genome-wide association studies (GWAS) [8, 9]. To date, GWAS have primarily been based on data from single nucleotide polymorphism (SNP) arrays [10, 11]. However, while mapping causal variants to large confidence intervals in the genome is possible with marker data, accurate identification of causal mutations requires whole-genome sequence (WGS) data [12]. Since WGS data should directly include the causal variants [13], analyses based on these do not require linkage disequilibrium (LD) between quantitative trait loci (QTL) and assayed SNPs [14]. Accordingly, the application of WGS data in GWAS should be beneficial [15] and has been demonstrated to increase both the significance level and the number of association signals compared to the use of SNP array data [16]. However, for WGS data to be advantageous, it is necessary to have WGS data for a considerable number of animals [17]. Yet, the sequencing of large samples of individuals remains expensive [18]. A cost-effective alternative to generate sequence-level data for large cohorts of animals is to apply genotype imputation [15], which is the prediction of genotypes for markers that are not directly genotyped in a study sample [19].

Although several studies have estimated genetic parameters for conformation traits in different horse breeds (e.g., [20–24]), little is known about the genomic background of horse conformation. Most studies on the association between morphological traits and genetic variants are based on candidate gene approaches (e.g., [25–28]), while only a few GWAS have been performed to identify genomic regions associated with equine conformation [6, 9, 29, 30]. Most of these GWAS are based on medium- or high-density SNP array data, and only one of the studies used imputed sequence-level genotypes [9].

One conformation trait that is of great importance in horse breeding is withers height, which is essential for the classification, appearance, function and performance of horses and a critical factor for their marketability [31]. Body size has also been associated with the health of the locomotor system [1] and with diseases such as equine recurrent laryngeal neuropathy [32]. Compared to other conformation traits, the genetic and genomic features of withers height have been investigated in more detail. GWAS conducted on various horse breeds have identified several loci associated with withers height or size on different chromosomes, including *Equus caballus* (ECA) chromosomes 3 [29, 33], 6 [33, 34], 9 [29, 33], and 11 [33, 35]. In particular, the QTL on ECA3 close to the *LCORL* (*ligand dependent nuclear receptor corepressor like*) and *NCAPG* (*non-SMC condensin I complex subunit G*) genes has been confirmed in several studies for diverse horse breeds [31, 32, 35–38]. This locus is also known to influence body size in numerous other species, including humans [39, 40], cattle [41, 42], sheep [43], pigs [44], rabbits [45], donkeys [46], chicken [47], and dogs [48]. To date, no causal variants underlying this QTL for withers height in horses have been identified, but there is growing evidence that *LCORL* retrocopies contributed to morphological changes during equid evolution and possibly also influence body size in present horses [49, 50].

Previous studies on the genomic background of equine conformation were often limited in sample size. For German Warmblood horses, which represent a large horse population of global importance, the availability of genotype data has considerably improved in recent years due to the initiation of a collaborative project of five German Warmblood breeding associations and partners from industry and science. Aiming at implementing genomic selection in sport horses, a reference population of 5000 warmblood horses with genotype and phenotype data was established [37, 51].

The aim of our study was to use this comprehensive dataset to identify genomic regions associated with withers height and linear conformation traits in German Warmblood horses by performing GWAS using imputed sequence-level genotypes. The investigated traits were further characterised by genetic parameter estimation. In addition, our aim was also to identify putative causal variants for withers height and correlated conformation traits. To verify the suitability of the imputed data set and the methodology, withers height was used as a reference trait, since it is objectively measurable and well characterised on both the genetic and genomic levels. The combined number of animals, markers, and traits included in the analyses is unique for the horse species and, to the best of our knowledge, no comparable study on conformation traits in horses has previously been conducted.

## Methods

### Dataset

Genotype and phenotype data were available for 5000 horses belonging to five German Warmblood breeding associations, namely Holstein (HOL), Oldenburg (OL), Oldenburg International (OS), Trakehner (TRAK), and Westfalian (WESTF). As these horses were meant to serve as a reference panel for genomic selection, they were selected based on the following criteria: (a) a low pedigree relationship (to broadly represent the current genetics of German Warmblood horses) and (b) a low level of preselection (achieved by primarily including mares in the panel) [37]. The population structure and the suitability of combining the five German Warmblood subpopulations into one large cohort for genomic applications were investigated and described by Vosgerau et al*.* [37]. A multidimensional scaling plot showing the population structure of the horses included in this study is in Additional file 1: Figure S1.

### Genotype data and imputation

The horses were genotyped using commercially available SNP arrays. The GGP Equine 70 k BeadChip (Neogen/Illumina) with 65,157 SNPs was used to genotype the first cohort of 788 individuals. All remaining 4212 horses were genotyped using the GGP Equine Plus BeadChip (Neogen/Illumina), that contains 6790 additional SNPs for a total of 71,947 SNPs [52]. The genotype data were lifted over to the new genome assembly EquCab3.0, separately for each group. Subsequently, samples and variants with a call rate lower than 0.9 were filtered out using the PLINK 1.9 software [53]. After merging the two datasets with different marker densities, phasing of the joint dataset was performed using the Beagle 5.1 [54] software with default parameter settings and the effective population size (N_e_) set to 1000. As a result, 63,049 SNPs were available for 4972 horses.

For these horses, genotype imputation to sequence level was performed using a reference panel of 175 horses that were compiled in a previous study [55]. Briefly, publicly available WGS data for 317 horses from 46 breeds were mapped against the reference genome EquCab3.0 and variant calling was performed using the Genome Analysis Toolkit (GATK) version 4.1.7.0 [56] according to the GATK best practices recommendations [57]. Subsequent investigations showed that the accuracy of imputing warmblood horses from medium marker density to sequence level was highest using the Beagle 5.1 software and a reference panel that included only a subset of the horses from the variant call set, i.e., 175 of the initial 317 horses. For this approach, the genome-wide imputation accuracy was 0.66 [55]. The 175 horses represented in the panel used were chosen based on their genetic relationship to warmblood horses and included mainly warmblood and Quarter horses, Arabians, Thoroughbreds, and Standardbreds.

Prior to imputation, the programme conform-gt version 24May16.cee [58] was used to make the markers genotyped on the warmblood horses consistent with the reference panel. As a result, 61,559 SNPs were available for subsequent analyses, of which 58,356 were located on autosomes. Imputation from medium marker density to sequence level was performed from the aforementioned 58,356 autosomal SNPs to 20,730,805 variants, using Beagle 5.1 [59] with default parameter settings and N_e_ set to 1000. Subsequently, variants with a minor allele frequency (MAF) lower than 0.01 were discarded using PLINK 1.9 [53], resulting in a set of 13,091,438 variants.

### Phenotype data

Available phenotype data for the genotyped horses included withers height and 81 conformation traits from linear profiling. The conformation data were collected applying the assessment scheme from the Oldenburg breeding association, which uses a seven-point scale from − 3 to + 3. The lowest and highest scores represented opposite biological extremes of the trait, while 0 was the average. For 20 of the 81 traits, representing defect traits, the scale only ranged from 0 to + 3.

The number of horses with both genotype and phenotype data ranged from 3113 to 4891 for the conformation traits and was equal to 2709 for withers height. All individuals with measurements for withers height were females and they were distributed among the breeding associations as follows: 1079 HOL, 614 OL, 169 OS, 553 TRAK, and 294 WESTF. The mares were measured between 2009 and 2020, at an average age of 4.3 ± 2.6 years. Measurements took place at the time of their studbook registration (SBR) or of the mare performance test (MPT). Withers height ranged from 153 to 180 cm, with a mean of 167.6 ± 3.6 cm.

The conformation data for the 4891 horses were collected between 2014 and 2019 at four different types of events: SBR (n = 1987), MPT (n = 1578), stallion pre-selection (SPS; n = 1201), and auction (AUC; n = 125). In total, 3614 mares and 1277 stallions were assessed by the five German Warmblood breeding associations HOL (n = 1288), OL (n = 1506), OS (n = 456), TRAK (n = 765), and WESTF (n = 876). At the time of assessment, the horses were on average 3.8 ± 2.2 years old. A detailed overview of the 81 conformation traits and their descriptive statistics is in Additional file 2: Table S1.

### Estimation of genetic parameters

To estimate the genetic variance explained by all SNPs and thereby the SNP-based heritability for all conformation traits, a genetic restricted maximum likelihood (GREML) analysis was performed using the Genome-wide Complex Trait Analysis (GCTA) version 1.93.2beta software [60, 61]. The analysis was based on the genetic relationship matrix (GRM) calculated from the portion of the 61,559 SNPs that had a MAF higher than 0.01 and that were located on autosomes (56,495 SNPs in total). In the case of withers height, only the age of the mares (in days) was included as a covariate. For the 81 conformation traits, the following fixed effects were considered in the analysis: age class (younger than 3 years old, between 3 and 4 years old, between 4 and 5 years old, and older than 5 years old at the time of assessment), sex (mare and stallion), judge (39 classes), and type of event at which the assessment took place (AUC, MPT, SBR, and SPS).

To determine the proportion of variance explained by single chromosomes and QTL, the analysis was repeated for withers height using multiple GRM, which were built separately for each chromosome and for two QTL on ECA1 and ECA3 that were detected in the subsequent GWAS. The QTL regions were defined based on visual inspection of the Manhattan plots, resulting in a 6-Mb region around the top associated SNPs (107.4 ± 3 Mb) on ECA3. To account for possible LD between the QTL and variants close to the defined region, variants within 14 Mb around those SNPs (100.4–114.4 Mb) were excluded from the GRM for the rest of ECA3. Similarly, the GRM for the QTL on ECA1 was built from a 2-Mb region around the top associated SNPs (55.2 ± 2 Mb), and a 4-Mb region (55.2–59.2 Mb) was excluded from the GRM for the rest of the chromosome.

All remaining analyses were performed only for the 61 conformation traits that had a *p*-value for the genetic variance calculated from the log-likelihood ratio test (LRT) in the GREML analysis lower than or equal to 0.05 (see Additional file 2: Table S1). For these traits (described in Table 1), the number of horses with available genotype and phenotype data ranged from 4768 to 4891.

**Table 1:** Coding, descriptive statistics and heritability estimates for 61 conformation traits in German Warmblood horses

Code	Trait description	N	Min	Max	Mean	SD	h^2^ ± SE
WithHeigh	Withers height	2709	153	180	167.587	3.563	0.528 ± 0.037
BrType	Breed type	4891	− 3	3	0.641	0.976	0.256 ± 0.023
GenExpr	Gender expression	4891	− 3	3	0.371	0.962	0.232 ± 0.023
Frame	Frame	4879	− 3	3	0.170	0.908	0.267 ± 0.026
Caliber	Caliber	4891	− 3	3	0.040	0.612	0.134 ± 0.022
ChestWt	Chest width	4769	− 3	3	− 0.017	0.423	0.048 ± 0.017
Barrel	Barrel	4769	− 3	3	0.008	0.309	0.030 ± 0.016
Condition	Condition	4891	− 3	3	0.032	0.397	0.045 ± 0.017
Develop	Development	4891	− 3	3	0.016	0.537	0.033 ± 0.015
LenLegs	Length of legs	4879	− 3	3	0.079	0.716	0.159 ± 0.022
HarmProp	Harmony of proportions	4879	− 3	3	0.186	0.655	0.067 ± 0.016
BodyShp	Body shape	4879	− 3	3	0.134	0.657	0.055 ± 0.017
BodyDir	Body direction	4879	− 3	3	0.027	0.353	0.046 ± 0.015
HeadShp	Head shape	4891	− 3	3	0.314	0.944	0.338 ± 0.024
HeadLen	Head length	4769	− 2	3	0.074	0.357	0.070 ± 0.017
EyeSize	Eye size	4891	− 3	3	0.172	0.705	0.104 ± 0.019
EyeColW	Eye colour (white)	4768	0	3	0.035	0.228	0.057 ± 0.018
HeadNeck	Head-neck connection	4769	− 3	2	0.001	0.360	0.049 ± 0.016
Cheeks	Cheeks (jowl)	4769	− 3	2	− 0.063	0.347	0.029 ± 0.014
LenNeck	Length of neck	4891	− 3	3	− 0.063	0.546	0.084 ± 0.019
SetNeck	Set of neck	4891	− 3	3	− 0.161	0.588	0.041 ± 0.016
MusNeck	Muscling area of neck	4769	− 3	3	0.062	0.595	0.110 ± 0.020
ShpNeckS	Shape of neck (straightness)	4769	− 3	3	− 0.025	0.560	0.085 ± 0.018
ShpNeckT	Shape of neck (thickness)	4768	− 3	3	− 0.021	0.430	0.064 ± 0.017
NeckWith	Neck connection to withers	4769	0	3	0.034	0.216	0.031 ± 0.014
LenWith	Length of withers	4891	− 3	3	0.094	0.681	0.087 ± 0.018
HeighWith	Height of withers	4891	− 3	3	0.113	0.680	0.105 ± 0.020
LenShoul	Length of shoulder	4891	− 3	3	0.084	0.449	0.025 ± 0.014
ShoulPos	Shoulder position	4769	0	3	0.035	0.219	0.027 ± 0.015
CoursTop	Course of topline	4891	− 3	3	− 0.028	0.510	0.043 ± 0.015
LenBack	Length of back	4891	− 3	3	0.124	0.609	0.107 ± 0.020
LineBack	Line (strength) of back	4891	− 3	3	− 0.175	0.567	0.103 ± 0.020
LineLoins	Line (strength) of loins	4891	− 3	3	− 0.056	0.608	0.077 ± 0.018
LenCroup	Length of croup	4891	− 3	3	− 0.029	0.567	0.035 ± 0.015
AngCroup	Angle (inclination) of croup	4891	− 3	3	0.085	0.647	0.089 ± 0.019
ShpCroup	Shape of croup	4769	− 3	3	− 0.004	0.300	0.023 ± 0.013
SetTail	Set of tail	4891	− 2	3	0.159	0.528	0.106 ± 0.020
PosCarp	Position of carpus	4769	− 3	2	0.026	0.278	0.049 ± 0.017
LenFLP	Length of forelimb pastern	4891	− 2	3	0.085	0.530	0.069 ± 0.017
StanFLP	Stance of forelimb pastern	4891	− 3	3	0.085	0.572	0.136 ± 0.020
ToeAxisFL	Broken toe axis in front limbs	4769	0	2	0.018	0.159	0.022 ± 0.014
ForeJoint	Definition of foreleg joints	4891	− 3	3	− 0.055	0.286	0.042 ± 0.016
CarCanArt	Definition of carpus-cannon articulation	4769	− 2	3	0.029	0.228	0.028 ± 0.015
LenHLP	Length of hind limb pastern	4891	− 3	3	0.040	0.420	0.064 ± 0.017
StanHLP	Stance of hind limb pastern	4891	− 2	3	0.125	0.543	0.078 ± 0.017
HockAng	Hock angulation	4891	− 3	3	0.129	0.762	0.113 ± 0.019
HindLeg	Hind leg (roundness)	4768	0	3	0.064	0.300	0.042 ± 0.015
CapHock	Capped hock	4891	0	3	0.100	0.375	0.063 ± 0.017
TarCanArt	Definition of tarsus-cannon articulation	4891	− 3	3	0.023	0.381	0.016 ± 0.011
SizeJoint	Size of joints	4769	− 3	3	− 0.094	0.510	0.097 ± 0.020
ShpFeet	Shape of feet (hoof size)	4891	− 3	3	− 0.032	0.544	0.188 ± 0.022
HeelHeigh	Heel height	4891	− 3	3	− 0.026	0.401	0.060 ± 0.016
HoofAsym	Hoof asymmetry (uneven shape of feet)	4879	0	3	0.045	0.263	0.031 ± 0.014
ToeStanFL	Toe stance of forelegs	4891	− 3	3	− 0.099	0.740	0.177 ± 0.022
SPosFL	Standing position of front limbs	4891	− 3	3	− 0.045	0.309	0.028 ± 0.014
ToeStanHL	Toe stance of hind legs	4891	− 3	3	0.013	0.225	0.023 ± 0.014
SPosHL	Standing position of hind limbs	4891	− 3	3	− 0.038	0.385	0.060 ± 0.017
PosHock	Position of hock—back view	4891	− 3	3	0.007	0.237	0.040 ± 0.015
CorrLMov	Correctness of limb movement	4879	− 3	3	0.010	0.317	0.025 ± 0.014
RotHock	Rotation in the hock	4769	0	3	0.078	0.336	0.049 ± 0.017
TailPos	Tail position	4769	0	3	0.058	0.286	0.034 ± 0.014
TailTone	Tail tone	4769	− 2	3	− 0.011	0.306	0.047 ± 0.014

N = sample size of horses with genotype and phenotype data for the respective trait, Min = minimum, Max = maximum manifestation reported, SD = standard deviation, h^2^ = heritability (ratio of genetic to phenotypic variance), SE = standard error

To estimate the genetic correlations between the 61 conformation traits and between conformation traits and withers height, a bivariate GREML analysis was performed using GCTA version 1.93.2beta [61, 62]. Only animals with available observations for all traits were used for this analysis, i.e., 4768 for the combination of two conformation traits and 2938 for the combination of one conformation trait and withers height. These analyses were based on the same set of variants as in the univariate GREML. Likewise, the fixed effects were the same as those used for the conformation traits in the univariate analysis, except that the effect of sex was omitted when withers height was included in the analysis, as withers height was only available for mares.

Subsequent GWAS revealed a significant association signal on ECA3 that was similar for withers height and 13 conformation traits. For these traits, an additional bivariate GREML analysis with multiple GRM was carried out to determine the genetic correlations for the QTL on ECA3 and the rest of the genome separately: one GRM was calculated from the SNPs in the QTL region on ECA3, defined as a 6-Mb region around the top associated SNPs (107.4 ± 3 Mb), and a second GRM was calculated using all autosomal SNPs except those in the QTL region on ECA3 (100.4–114.4 Mb, to account for possible LD structure).

### Genome-wide association studies

To identify genomic regions associated with each trait, a mixed linear model (MLM) based GWAS was performed for withers height and all 61 conformation traits in GCTA version 1.93.2beta [61, 63]. The analysis was performed using a leaving-one-chromosome-out (LOCO) approach, i.e., the GRM was calculated based on the 56,495 medium-density SNPs that were located on autosomes and had a MAF higher than 0.01, except those on the chromosome where the candidate SNP was located. For withers height, the MLM was as follows:

$${\text{WH}}_{ijk} = \mu + \beta_{1} {\text{age}}_{i} + \beta_{2} {\text{SNP}}_{j} + g^{ - } + e_{ijk} ,$$

where ${\text{WH}}_{ijk}$ is the withers height of the $k$th animal, $\mu$ is the overall mean, ${\text{age}}_{i}$ is the covariate of age at the time of assessment (in days), ${\text{SNP}}_{j}$ is the SNP genotype indicator variable ($j$ = 0, 1, 2; representing the number of minor alleles), ${g}^{-}$ is the additive genetic effect of all SNPs except those on the chromosome where the candidate SNP is located, ${\beta }_{1}$ and ${\beta }_{2}$ are the respective regression coefficients, and ${e}_{ijk}$ is the random residual effect.

For all conformation traits, the following MLM was used:

$${\text{CT}}_{ijklmn} = \mu + {\text{age}}_{i} + {\text{sex}}_{j} + {\text{judge}}_{k} + {\text{event}}_{l} + \beta {\text{SNP}}_{m} + g^{ - } + e_{ijklmn} ,$$

where ${\text{CT}}_{ijklmn}$ is the phenotype of the $n$th animal, $\mu$ is the overall mean, ${\text{age}}_{i}$ is the fixed effect of the $i$th age class at the time of assessment ($i$ = 3, 4, 5, 6; representing the following age classes: horses younger than 3 years old, between 3 and 4 years old, between 4 and 5 years old, and older than 5 years old), ${\text{sex}}_{j}$ is the fixed effect of the $j$th sex of the horse ($j$ = mare or stallion), ${\text{judge}}_{k}$ is the fixed effect of the $k$th judge assessing the horse ($k$ = 1, …, 39), ${\text{event}}_{l}$ is the fixed effect of the $l$th event where the assessment took place ($l$ = AUC, MPT, SBR, and SPS), $\beta$ is the fixed effect of the candidate SNP, ${\text{SNP}}_{m}$ is the additive genotype code of the $m$th SNP ($m$ = 0, 1, 2; representing the number of minor alleles), ${g}^{-}$ is the additive genetic effect of all SNPs except those on the chromosome where the candidate SNP is located, and ${e}_{ijklmn}$ is the random residual effect.

Subsequently, to verify the identified QTL effect, a conditional GWAS was performed for withers height and the 13 conformation traits that showed a significant association signal on ECA3. In the case of withers height, the top associated SNP, rs68603062, identified in the preliminary GWAS was included in the model as an additional fixed effect to test if the discovered peak was caused by a single QTL. For the conformation traits, three different scenarios were considered: either the top associated SNP from the GWAS for the respective conformation trait itself, the top associated SNP from the GWAS for withers height, or withers height itself was included in the initial model as an additional fixed effect. The two latter scenarios were conducted to test for possible pleiotropic effects between the conformation traits and withers height.

For all GWAS, a genome-wide significance threshold was set by applying a Bonferroni correction (i.e., p ≤ 0.05 divided by the number of tests) to account for multiple testing. Manhattan plots were generated using R version 4.0.3 [64].

### Identification of putative causal variants for withers height

To identify putative causal variants for withers height in horses, we further investigated the QTL detected on ECA3. For this purpose, two complementary approaches were applied, one based on LD structure and one based on identical-by-descent (IBD) sharing. Finally, to relate the findings of both methods to functional annotations, the Ensembl Variant Effect Predictor (VEP) release 104 web interface [65] was used to predict the effect of the 20,960 variants that showed a statistically significant association with withers height. Standard settings were applied for the horse species, except that the distance to the transcript for which VEP assigns upstream and downstream consequences was set to 100,000 bp.

For the LD-based approach, the LD structure between the 20,960 significantly associated variants and the top SNPs from GWAS was determined using the option --r2 in PLINK 1.9 [53] to define a set of putative candidate mutations. Variants in high LD with the top SNP (R^2^ > 0.8) were considered as potentially causal and their annotations from variant effect prediction were taken into account to assess the plausibility that they affect withers height in horses.

For the approach based on IBD sharing, first, a GREML analysis was performed using the option --reml-pred-rand in GCTA version 1.93.2beta [60, 61], which uses the best linear unbiased prediction (BLUP) method, to predict the genomic estimated breeding value (gEBV) for withers height for all 2709 mares with genotype and phenotype data. To obtain the chromosomal gEBV for withers height specific to ECA3 (ECA3 gEBV), two GRM were fitted in the model: one calculated from the SNPs on ECA3 and one built from the SNPs on all autosomes except ECA3. The age of the horses was included as a fixed effect.

Subsequently, the ten horses with the highest and the ten horses with the lowest ECA3 gEBV for withers height were used to detect runs of homozygosity (ROH) in the QTL region using the flag --homozyg in PLINK 1.9 [53] to identify potential selection targets for withers height. Our approach was based on the following assumptions: (a) ROH that are shared between individuals are not only identical-by-state but also IBD; (b) there is a single founder mutation either for the “tall” or the “short” allele; (c) as the QTL explains a comparatively large proportion of the variance, it is expected that in one of the extreme groups, short or tall, the founder haplotype is enriched, i.e., there are also more homozygotes; and (d) given the high heritability of withers height, it is more appropriate to use chromosomal gEBV than phenotypes in these analyses.

ROH detection was performed for the region on ECA3 within which genome-wide statistically significant variants were identified in the preceding GWAS (97,840,010–117,983,557 ± 100,000 bp). In the first round of analysis, the horses with the highest and the lowest ECA3 gEBV were considered separately. In the second run, all 20 horses were analysed simultaneously, but including the information about their phenotype (high vs. low ECA3 gEBV). ROH were required to contain at least 50 SNPs and have a minimum length of 100 kb, with a density of at least one SNP per 50 kb. Consecutive SNPs separated by a distance of more than 1000 kb were not permitted in the same ROH. In each scanning window of 50 SNPs, not more than three heterozygous and five missing genotypes were tolerated. High- and moderate-impact variants that were significantly associated with withers height and located within the identified ROH were further considered in search of potential causal mutations.

Finally, the results from both approaches (based on LD and on IBD sharing) were combined, and shared candidate mutations were further investigated. The LD between these candidate variants was calculated using the option --ld in PLINK 1.9 [53]. AlphaFold version 2.3.0 [66] was applied to predict the structure of the proteins that were altered by the candidate mutations, which was compared to the wild-type proteins. For this purpose, protein sequences were obtained from Ensembl release 104 [67] in accordance with the VEP results. The predicted protein structures were visualised using the UCSF ChimeraX version 1.5 software [68] and the relaxed models with the highest confidence. To validate the imputed genotypes, putative causal variants were Sanger-sequenced at Microsynth Seqlab GmbH (Göttingen) after amplification of the target DNA segments using the primers listed in Additional file 3: Table S2. For this purpose, the ten horses with the highest and the ten horses with the lowest ECA3 gEBV for withers height were chosen.

## Results

### SNP-based heritability

**Fig. 1:**
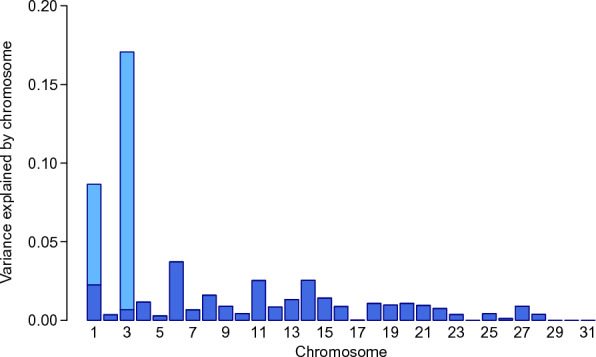
Estimated proportions of the phenotypic variance for withers height. Proportions of the variance explained by each of the 31 equine autosomes (dark blue) and the two quantitative trait loci on chromosomes 1 and 3 (light blue). Summed up, the marker-based heritability for withers height was 0.51

For withers height, the SNP-based heritability estimated by GREML analysis was 0.53 (± 0.04). When fitting multiple GRM, which were built separately for each autosome and the two QTL, in the model, the heritability estimate was slightly lower (0.51 ± 0.04). The, by far, largest proportion of the phenotypic variance for withers height was explained by ECA3 (17.1%, i.e., about 33% of the genetic variance originated from this chromosome), followed by ECA1 (8.7%) and ECA 6 (3.7%). On ECA3, almost all of the variance was explained by the QTL between 104 and 110 Mb that was identified in the subsequent GWAS (see “GWAS for withers height”). The estimated proportions of the phenotypic variance explained by each of the autosomes and the QTL are shown in Fig. 1.

For the conformation traits, the estimated proportion of the phenotypic variance explained by all autosomal SNPs ranged from 0 to 0.34. The highest SNP-based heritability was predicted for head shape (0.34), frame (0.27), breed type (0.26), and gender expression (0.23). Heritability estimates for all 81 conformation traits are shown in Additional file 2: Table S1. For 20 traits, the GREML *p*-value was larger than 0.05, i.e., those traits showed no or very little genetic variance and were therefore excluded from further investigations and all subsequent analyses, i.e., GWAS and the estimation of genetic correlations, were conducted for the remaining 61 conformation traits listed in Table 1.

### GWAS for withers height

**Fig. 2:**
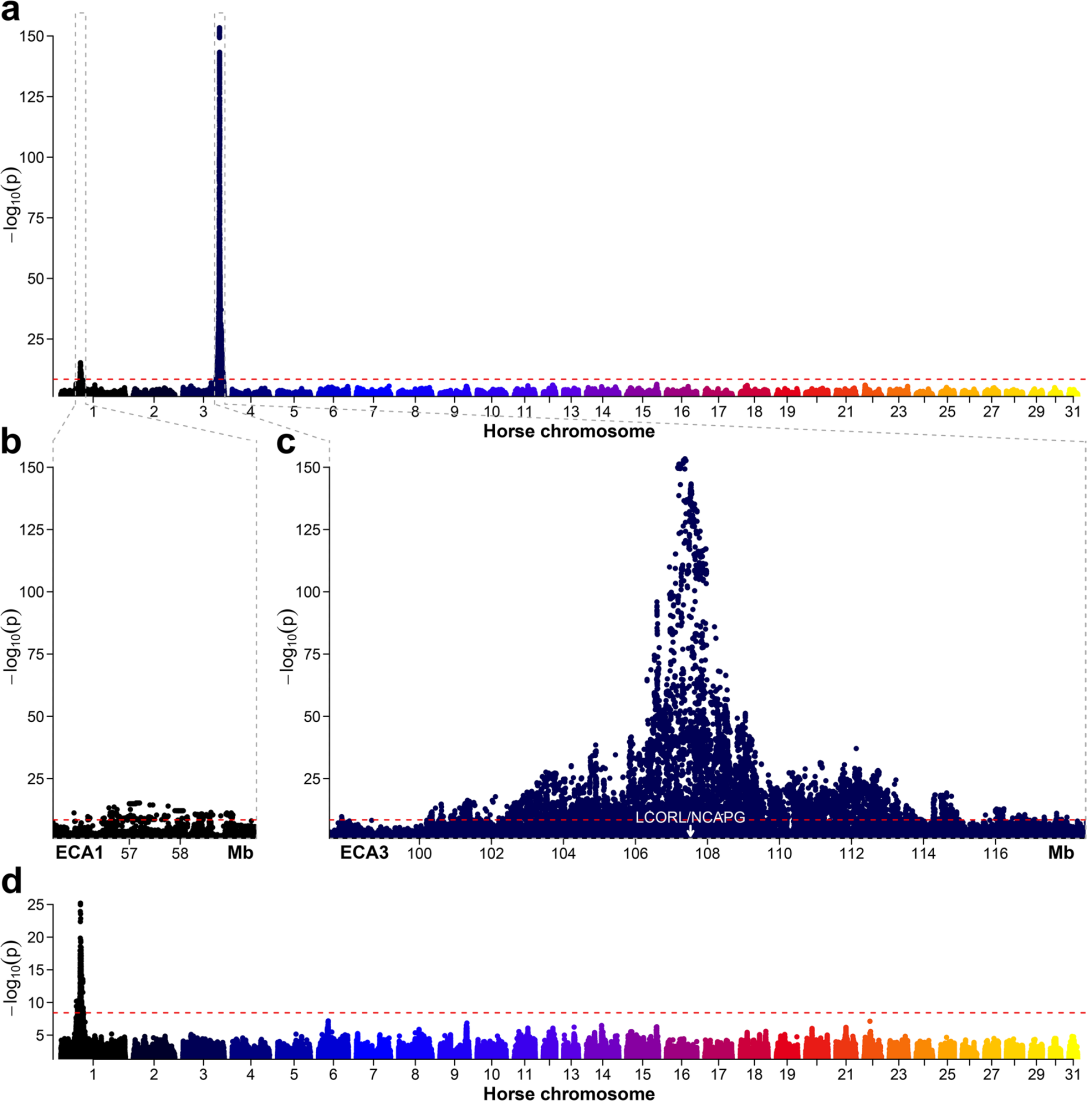
Results of the genome-wide association study for withers height in 2709 mares. Manhattan plots of the –log_10_ *p*-values for the association of variants with withers height for the whole genome (**a**) and the two quantitative trait loci on *Equus caballus* chromosome 1 (ECA1) (**b**) and 3 (ECA3) (**c**). In **d**, the top associated SNP rs68603062 from the preliminary GWAS (**a**) was included in the model as an additional fixed effect. The red dashed lines indicate the genome-wide significance threshold with α = 0.05 and Bonferroni correction for multiple testing (*p* = 3.8 × 10^–9^). Due to computational limitations, variants with a *p*-value > 0.05 were excluded from the plots

GWAS for withers height revealed two genome-wide significant association signals between 55.91 and 59.04 Mb on ECA1 and between 97.84 and 117.98 Mb on ECA3 (Fig. 2a). On ECA1, 22 SNPs located between 57,132,845 and 57,201,369 bp that all had a MAF of 0.08 showed the same lowest empirical *p*-value for this chromosome (*p* = 6.3 × 10^–16^) (Fig. 2b). On ECA3, four SNPs showed the lowest empirical *p*-value of 4.2 × 10^–154^ (Fig. 2c), i.e., rs68603062 (position on EquCab3.0: 107,373,887 bp), rs68603064 (107,374,136 bp), rs1136437528 (107,374,798 bp), and rs1149496287 (107,375,521 bp). For the 2709 horses included in the GWAS, all four SNPs had a MAF of 0.47. They were located upstream of the *LCORL* gene and downstream of the *NCAPG* gene.

When including the top associated SNP, rs68603062, as a fixed effect in the MLM, the peak on ECA3 completely disappeared. Only the signal on ECA1 was genome-wide significant in that case, with the top associated SNPs showing a lower empirical *p*-value than before (*p* = 6.5 × 10^–26^) (Fig. 2d). The quantile–quantile (QQ) plots for the initial and the conditional GWAS for withers height are shown in Additional file 4: Figure S2.

### GWAS for conformation traits

For 33 of 61 conformation traits, at least one genome-wide significantly associated SNP could be detected by GWAS (all discovered QTL are listed in Additional file 5: Table S3 and the corresponding Manhattan plots are in Additional file 6: Figure S3). For 14 of these 33 traits, significantly associated variants were identified on more than one chromosome, and for 13 of the 33 traits, a significant association signal was detected on ECA3 in the region also associated with withers height: breed type, gender expression, frame, caliber, length of legs, body shape, head shape, head length, eye size, length of withers, size of joints, shape of feet (hoof size), and rotation in the hock. For these 13 traits, conditional GWAS were performed. When including the top associated SNP rs68603062 from the GWAS for withers height or the top associated SNP from the GWAS for each respective trait (see Additional file 7: Table S4) as a fixed effect in the model, the significant signal on ECA3 dropped completely in all cases. Inclusion of withers height as a quantitative covariate resulted either in a reduction or the disappearance of the peak, depending on the trait (Manhattan plots are in Additional file 8: Figure S4). Association signals other than those on ECA3 and including at least five genome-wide significant SNPs are listed in Table 2.

**Table 2:** Quantitative trait loci identified in genome-wide association studies for conformation traits in horses

Trait	ECA	Region (start–end bp)	Number of SNPs	*p*-value
Frame	1	56,598,762–57,762,540	97	1 × 10^–11^
ChestWt	1	172,700,779–177,438,466	100	6 × 10^–15^
EyeColW	1	24,334,579–24,633,589	16	7 × 10^–10^
	1	75,206,687–82,273,853	281	1 × 10^–12^
	3	72,436,610–80,584,767	274	2 × 10^–13^
	8	89,278,998–89,306,076	7	3 × 10^–12^
	16	18,919,564–21,266,169	13	2 × 10^–11^
	26	17,095,748–17,380,416	122	4 × 10^–13^
Cheeks	9	18,897,064–18,953,488	72	3 × 10^–11^
LenNeck	27	22,220,567–22,361,542	8	3 × 10^–09^
NeckWith	1	103,863,865–103,875,329	5	2 × 10^–09^
	10	59,197,862–59,954,346	128	4 × 10^–15^
	17	50,745,878–50,850,775	22	5 × 10^–10^
ShoulPos	6	22,988,914–23,562,243	12	3 × 10^–11^
	10	47,051,242–47,057,801	27	5 × 10^–11^
	16	79,426,355–79,487,232	10	6 × 10^–13^
ToeAxisF	10	64,329,104–64,339,469	5	1 × 10^–09^
	12	15,232,635–15,435,584	5	1 × 10^–10^
ForeJoint	1	125,317,343–125,333,785	46	3 × 10^–10^
ShpFeet	2	33,318,116–37,600,770	59	5 × 10^–11^
HoofAsym	17	64,636,582–67,185,489	37	5 × 10^–10^
ToeStanFL	22	31,767,254–32,395,663	191	5 × 10^–11^

Only loci that included at least five genome-wide significant single nucleotide polymorphisms (SNPs) and were not located between 97 and 118 Mb on ECA3 are listed. Trait codes are given in Table 1. ECA = *Equus caballus*, number of SNPs = number of SNPs that were genome-wide significantly associated with each respective trait at the given locus, *p*-value = *p*-value of the most significantly associated SNP at the given locus

### Genetic correlations

**Fig. 3:**
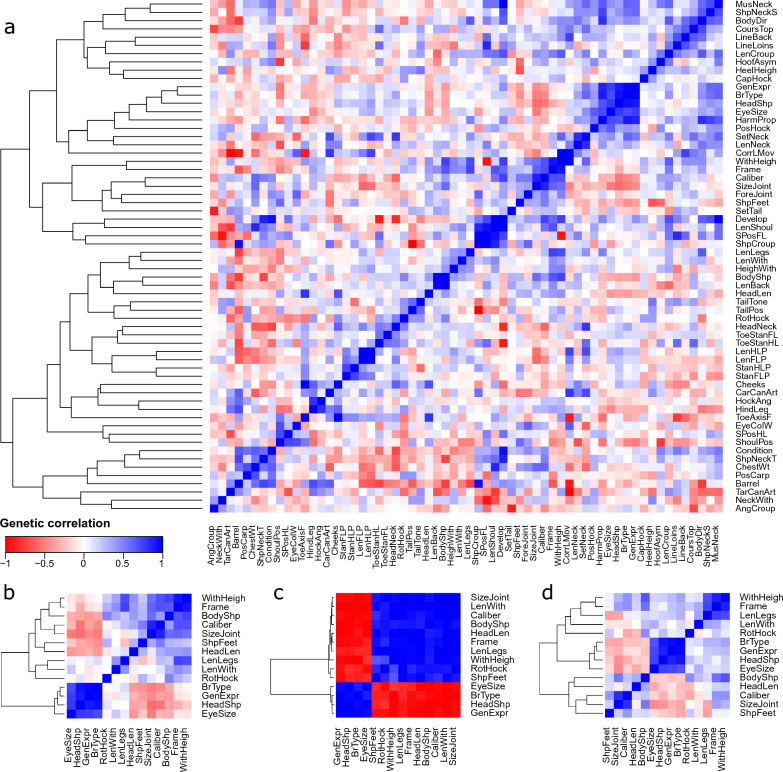
Genetic correlations of 61 conformation traits with withers height in horses. **a** includes all investigated traits. **b**–**d** only include the traits for which a genome-wide significant association signal on *Equus caballus* chromosome 3 (ECA3) between 97 and 118 Mb was detected and show their correlations for all autosomes (**b**), only ECA3 (**c**) and all autosomes except ECA3 (**d**). Trait codes are described in Table 1

Estimates for genetic correlations between conformation traits covered the full range from − 1 to + 1 (Fig. 3a and see Additional file 9: Table S5). The strongest negative correlation was predicted between tail position and shape of croup. A positive correlation of 1 was estimated for six trait combinations, e.g., body shape with length of back and length of forelimb with hindlimb pastern. However, due to the large standard errors that were estimated for some of the correlations, the estimates should be interpreted with caution.

Estimates of genetic correlations between the 13 conformation traits that showed a significant association signal on ECA3 and between these traits and withers height varied considerably, with some correlations close to 0 (Fig. 3b and Additional file 9: Table S6). However, when considering the QTL on ECA3 separately from the rest of the genome, high to very high positive (> 0.65) or negative (< − 0.79) genetic correlations were estimated for the QTL on ECA3 for all trait combinations (Fig. 3c). Estimates of genetic correlations for the remaining genome had opposite signs to those for the QTL on ECA3 in several cases, resulting in an overall correlation estimate close to 0 (Fig. 3d and see Additional file 9: Table S6).

### Identification of putative causal variants for withers height

Variant effect prediction annotated 93,863 effects to the 20,960 variants that were significantly associated with withers height, 71.5% of which were known and 28.5% were novel. Four variants, all located on ECA3, were predicted to have a high impact on protein-coding sequences. Of the 32 variants with a predicted moderate impact on protein-coding sequences, 27 were located on ECA3 and five on ECA1 (see Additional file 10: Table S7).

The LD-based approach revealed that 190 of the significantly associated variants on ECA3 were in high LD (R^2^ > 0.8) with the top SNPs from GWAS, all located between 107,007,474 and 107,796,559 bp (see Additional file 11: Table S8). The vast majority of these mutations was annotated only with modifying effects, while one variant (rs1146838995) was also predicted to have a high impact and four variants (rs1148715914, rs1138481672, rs1139684227, and rs1137124154) a moderate impact on the *LCORL*, *NCAPG* or *DCAF16* genes (Table 3). Hence, these five variants were further considered as putative candidate mutations using the LD-based approach.

**Table 3:** Putative causal variants on ECA3 for withers height in German Warmblood horses

Position	rsID	Alleles	MAF	DR2	b ± SE	*p*-value	Consequence	Gene	AA	R^2^
107,558,421	rs1146838995	G/T	0.45	0.80	2.68 ± 0.11	7 × 10^–141^	Stop gained	*LCORL*	E/*	0.91
107,558,814	rs1148715914	A/T	0.45	0.84	2.69 ± 0.11	3 × 10^–141^	Missense	*LCORL*	I/F	0.91
107,560,909	rs1138481672	T/G	0.45	0.80	2.67 ± 0.11	5 × 10^–140^	Missense	*LCORL*	L/R	0.90
107,624,530	rs1139684227	T/C	0.44	0.82	2.53 ± 0.11	7 × 10^–127^	Missense	*NCAPG*	T/A	0.81
107,633,983	rs1137124154	G/A	0.45	0.82	2.58 ± 0.11	2 × 10^–133^	Missense	*DCAF16*	R/K	0.86

Moderate and high impact variants that were genome-wide significantly associated with withers height, in high linkage disequilibrium (R^2^ > 0.8) with the top single nucleotide polymorphisms (SNPs) from a genome-wide association study (GWAS) and located on *Equus caballus* chromosome 3 (ECA3) within two runs of homozygosity shared by ten horses with a high genomically estimated breeding value for withers height (based on the markers on ECA3). MAF = minor allele frequency (the major allele is indicated first), DR2 = dosage R-squared value from imputation with Beagle 5.1, b = SNP effect of the minor allele, SE = standard error and *p*-value = *p*-value for the respective variant obtained in a GWAS including 2709 horses, AA = amino acids, R^2^ = squared correlation to the top SNPs from GWAS

**Fig. 4:**
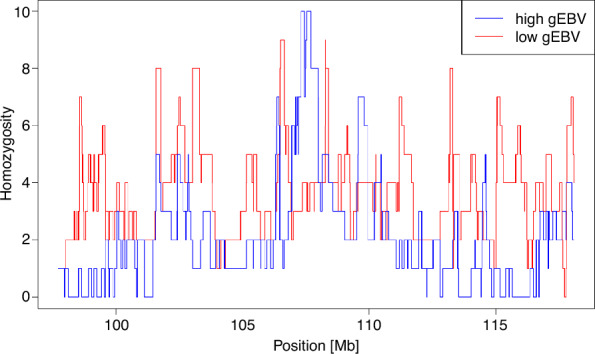
Overlapping runs of homozygosity (ROH) at a quantitative trait locus (QTL) for withers height. Number of horses with a high (blue) and low (red) chromosomal genomically estimated breeding value (gEBV) for withers height for which an ROH was detected at the respective position within the QTL on horse chromosome 3

The gEBV for withers height ranged from − 4.49 to 4.15 for all autosomes excluding ECA3, and from − 5.90 to 5.08 for ECA3. The chromosomal gEBV for ECA3 for the ten horses with the highest values ranged from 4.15 to 5.08 and those for the ten horses with the lowest values ranged from − 5.90 to − 4.08. Based on the IBD sharing method, for the ten horses with the highest ECA3 gEBV for withers height, 42 overlapping ROH were detected on this chromosome. Two of these ROH, which were located between 107,308,144 and 107,435,503 bp and between 107,545,201 and 107,706,771 bp, were shared by all ten individuals (Fig. 4). For the ten horses with the lowest ECA3 gEBV, 56 overlapping ROH regions were identified, but none of them was shared by all animals. When considering the horses with a high and a low ECA3 gEBV simultaneously, four ROH were detected that were shared between all ten individuals with a high gEBV and four of the individuals with a low gEBV. However, the latter carried a different allele for these ROH than the former. All four ROH were located within the two ROH that were shared by the ten horses with a high ECA3 gEBV (at 107,368,362–107,435,503 bp, 107,545,201–107,548,746 bp, 107,548,790–107,565,464 bp, and 107,661,952–107,706,771 bp).

Among the variants located within the two ROH that were shared by all ten horses with a high ECA3 gEBV for withers height, three were annotated with a high and six with a moderate impact (see Additional file 10: Table S7). Five of these nine variants detected by the IBD sharing method were identical to the set of candidate mutations identified by the LD-based approach. Therefore, these five variants, presented in Table 3, were considered the most likely causal mutations for withers height and were investigated further. All five candidate variants were in high LD with each other (R^2^ > 0.83), with the highest R^2^ values of 0.99 to 1.00 between the SNPs rs1146838995, rs1148715914 and rs1138481672. The LD between these three variants and the rs1139684227 and rs1137124154 SNPs was slightly lower (0.83 and 0.88, respectively), and it was 0.94 between the latter two SNPs. For all five candidate variants, the protein structure prediction revealed obvious differences from the wild-type proteins for the respective mutations (see Additional file 12: Figure S5).

Sanger sequencing data perfectly confirmed the imputed genotype distribution for the three SNPs rs1146838995, rs1148715914, and rs1138481672. All three SNPs were homozygous for the reference (major) allele in all ten horses with the lowest ECA3 gEBV and homozygous for the alternate (minor) allele in all ten horses with the highest ECA3 gEBV. The same applied to the rs1139684227 SNP, for which the sequencing results deviated slightly from the imputed genotypes (two of the ten horses with the highest ECA3 gEBV were heterozygous in the imputed data). In contrast, the rs1137124154 SNP was alternatively homozygous for the horses with the lowest (reference allele) versus the highest (alternate allele) ECA3 gEBV in the imputed data, but Sanger-sequencing revealed that three of the ten horses with the highest ECA3 gEBV were heterozygous.

## Discussion

In this study, a comprehensive dataset of German Warmblood horses was used to characterise withers height and a number of conformation traits at the genetic and genomic levels.

### SNP-based heritability

Heritability estimates for the conformation traits were low to moderate (from 0 to 0.34) and thus within a similar range as reported for linear type traits in the literature [20, 21, 23]. However, in our study, many traits showed a fairly low heritability, with estimates below 0.1 for 65 of the 81 traits studied. Besides the fact that a large proportion of the variance in some of the traits may indeed not be genetically determined, a major reason for the low heritability estimates is probably inadequate phenotype recording. Phenotyping in horses is a difficult task which often lacks objectivity and repeatability due to the strong influence of the judging person [9]. Linear profiling, the description of traits on a scale between two biological extremes in relation to the population mean, was expected to overcome some of the main shortcomings of the traditional scoring system by improving objectivity and trait definition [69]. However, it turned out that one of the main problems of the traditional grading scheme, i.e., insufficient use of the scoring scale, was also an issue in linear scoring [7]. In our dataset, on average, only 9 of the 81 conformation traits per horse deviated from 0, resulting in a rather low variance for many of the reported traits.

The estimated heritability was notably higher for withers height (0.53) than for all other conformation traits. In contrast to the linear traits, withers height is objectively measurable and not subject to the influence of the assessor. Heritability estimates were very similar to those from other studies in warmblood horses (0.49 [1], 0.53 [36], 0.57 [70]) and in the centre of the wide range of values for other horse breeds (0.28 to 0.80 [3, 21, 24, 30, 71]).

### Withers height

GWAS for withers height revealed one genome-wide significant association signal on ECA1 and one on ECA3. The QTL on ECA3 has already been reported in various studies, both in warmblood horses [31, 36] and in other horse breeds [29, 33]. Similar to the observations made in other studies [29, 36], the largest proportion of the variance for withers height could be explained by the QTL on ECA3, followed by ECA1. Accordingly, the detection of the peak on ECA3 could not only confirm the presence of a QTL for withers height on this chromosome but could at the same time show the suitability of our dataset and methodology for further analyses.

One of the four top associated SNPs, rs68603064 (BIEC2_808543), was likewise identified to be the best-associated marker in previous studies using SNP array data [29, 31–33]. However, *p*-values were considerably lower in our study using imputed sequence-level genotypes. The inclusion of one of the top associated SNPs (rs68603062) as a fixed effect in the model removed the peak on ECA3, suggesting the presence of only one single QTL for withers height in this region of the chromosome, as already stated by Vosgerau et al*.* [37].

By combining the GWAS results with variant effect prediction, LD structure analysis, and ROH detection, we were able to narrow down the pool of putative causal mutations for withers height to five variants located in the *LCORL*, *NCAPG*, and *DCAF16* (*DDB1 and CUL4 associated factor 16*) genes. The *NCAPG* gene encodes one of the three non-structural maintenance of chromosomes (SMC) subunits of the mammalian condensin I complex [72], which plays an important role for chromosome condensation during mitosis [73]. The *LCORL* gene encodes a transcription factor that may be involved in spermatogenesis [74], and *DCAF16* encodes a substrate recognition component of the CUL4-DDB1 E3 ubiquitin ligases [75].

The *NCAPG*/*LCORL*-locus has been associated with body size in various other species, including humans [40], cattle [41], sheep [43], and pigs [44], and therefore represents a strong candidate locus for withers height in horses. These previous findings, in combination with the very low *p*-values in the GWAS performed here, the high LD with the top SNPs, the predicted impacts of the variants, and their location within a shared ROH, especially, make the three SNPs rs1146838995, rs1148715914, and rs1138481672 that are located in the coding sequence (CDS) of the *LCORL* gene, and the SNP rs1139684227 in the CDS of the *NCAPG* gene, good candidates as causal variants for withers height in horses. For these variants, all sequenced horses with a high ECA3 gEBV were homozygous for the alternate (minor) allele, while all horses with a low ECA3 gEBV were homozygous for the reference (major) allele. Hence, our results indicate that for these four SNPs, which were in high LD with each other, the alternate alleles result in an increase and the reference alleles in a decrease in size of the horses. Looking at the estimated SNP effect per variant, one copy of the corresponding alternate allele is expected to increase withers height by 2.53 to 2.69 cm. Hence, homozygous horses for the alternate allele are on average about 5 cm higher than those homozygous for the reference allele.

The IBD sharing approach to identify putative causal mutations was based on the hypothesis that the identified ROH were IBD. This remains an assumption, especially because three heterozygous SNPs were allowed in each scanning window for ROH detection to account for possible imputation errors. Another important assumption in this approach was that, given the large effect of the QTL on ECA3, the horses with the most extreme ECA3 gEBV (either high or low) should be homozygous for the haplotype that carries the founder mutation. In fact, due to the use of imputed genotypes, the true QTL genotypes were unknown, which should be kept in mind when interpreting the results. However, the dosage R-squared (DR2) values of all candidate mutations were reasonably high (> 0.8) and Sanger-sequencing confirmed the alternative homozygous genotype distribution of the horses with a high or low ECA3 gEBV for the four candidate SNPs in the *LCORL* and *NCAPG* genes. Therefore, the effect of imputation errors can be regarded as rather low, at least for these specific variants.

In contrast, Sanger-sequencing did not fully confirm the alternative homozygous genotype distribution for the fifth SNP, rs1137124154, which was located in the only exon of the *DCAF16* gene. Due to this deviation from the imputed data and the nature of the affected gene (no known candidate gene for size), this variant was assumed to be less likely causal for withers height compared to the other four. Overall, from our dataset, the four SNPs, rs1146838995, rs1148715914, rs1138481672, and rs1139684227, can be considered as the most likely candidate causal variants for the QTL on ECA3 for withers height in horses.

The three candidate variants in the CDS of *LCORL* were in almost perfect LD, i.e., the three mutations only appeared together. As the nonsense mutation at rs1146838995 is located upstream of the two missense mutations at rs1148715914 and rs1138481672, the latter two are not expected to have an impact because the protein is already truncated after amino acid 817 due to the presence of the mutation at rs1146838995. Hence, among these three variants, rs1146838995 can be assumed to be the only one with an actual effect, while the other two are “silent” in combination with the former. Remarkably, mutations resulting in the emergence of truncated LCORL proteins have also been reported to be associated with body size in other species, such as dogs and goats [76, 77]. According to Ensembl release 104 [67] for both these species and also for other species (e.g., humans and mice), there are several *LCORL* transcripts, which can be classified into long and short ones. Likewise, four different transcripts are listed for horses, with one being long (1871 amino acids) and three considerably shorter (320, 556 and 603 amino acids). The three candidate mutations were predicted to affect only the long transcript ENSECAT00000057726.2, namely exon 7 of the eight exons, which is not present in the other transcripts. To determine the expression levels of the different *LCORL* transcripts, additional experimental analyses are required. However, mapping of RNA sequencing reads derived from three horse brains (BioProject PRJEB33353, BioSamples SAMEA5756304, SAMEA5756306 and SAMEA5756308), following the methodology described by Falker-Gieske et al*.* [78], showed that the region that contains the nonsense mutation was covered by RNA-sequencing reads for two of these three brain transcriptome sequences, which confirms the general existence of the long transcript ENSECAT00000057726.2.

In the German warmblood horses, the nonsense mutation, rs1146838995, which affects the long *LCORL* transcript, was associated with an increase in withers height. Likewise, in dogs, a single base pair insertion in the terminal exon of only the long *LCORL* transcript, which introduces a premature stop codon, was primarily observed in large-sized breeds and only at low frequencies in medium-sized breeds, while it was not present at all in small-sized breeds [76]. Furthermore, there is evidence for a possible association between truncating mutations in the *LCORL* gene and an increased body size in goats [77]. As the long isoform of the canine and caprine LCORL protein comprises a DUF4553 DNA-binding domain, it was hypothesised that truncation of the protein might prevent the binding of the transcription factor LCORL to its target [76, 77]. Similarly, the long horse transcript also contains a DUF4553 domain [79], which is missing in the protein truncated by the nonsense mutation. Our findings, in combination with the results from other studies, highlight the nonsense mutation in exon 7 of the long transcript of *LCORL* as a good candidate causal mutation for withers height in warmblood horses.

However, a recent study has identified retrocopies in the *LCORL* gene that may have played a role in morphological changes during the evolution of equids and also affect body size in present horses, possibly by altering the translation or expression of their parent gene [49]. Considering these findings, it is also possible that the SNPs that we detected here are in LD with structural variants that influence body size rather than being causal themselves. The authors of the aforementioned study [49] argue that the initial retrotransposition that occurred during the evolution of equids coincided with major morphological changes such as increased size, decreased digit number, and altered dentation, which suggests a functional role of the *LCORL* gene in these changes. This is in line with the association of this gene with various morphological traits in our study, as well as with the results of Ricard et al*.* [38], who found associations with overall development, i.e., height, length, and width, but not with withers height when standardised to unit centroid size.

In contrast to the QTL on ECA3, the peak on ECA1 has rarely been described. Other studies have reported that relatively large fractions of the variance for withers height result from this chromosome but could not detect any significantly associated variants [29, 36]. Investigations in American miniature horses [80] and Shetland ponies [81] revealed an association of reduced body size with variants on ECA1 but these were located in other chromosomal regions than that identified in our study. However, one study on Belgian draft horses detected a single significant association signal on ECA1 close to the *MYPN* (*myopalladin*) gene [82] and hence at a similar location as the QTL that we identified for German Warmblood horses. Furthermore, a single significantly associated SNP was previously detected in this region when performing GWAS for withers height using SNP array data for a subset of the horses included in the present study [37]. A very recent study on the morphology of French trotters also detected this QTL [38].

Variant effect prediction for significantly associated SNPs identified five variants on ECA1 with a moderate impact on five genes: *MYPN*, *TET1*, *MACROH2A2*, *NPFFR1*, and *EIF4EBP2*. For three of these genes, according to the NHGRI-EBI GWAS Catalog [83] (accessed September 2022), one or more variants had previously been associated with body height in humans: *MYPN*, *TET1*, and *NPFFR1*. Hence, the missense variants that we identified in these genes could represent putative causal mutations that affect withers height in horses. However, further investigations are necessary to confirm our findings.

Apart from the QTL on ECA1 and ECA3, we could not identify any of the other loci that have been reported to be associated with withers height in different horse breeds, such as those on chromosomes 6, 9, and 11 [33]. Nevertheless, in our dataset, ECA6, ECA11, and ECA14 explained the next largest proportions of variance for withers height, after ECA3 and ECA1.

### Conformation traits

GWAS for 61 conformation traits revealed association signals on 23 chromosomes. While the majority of the QTL were unique to specific traits, the region on ECA3 that was associated with withers height likewise showed associations with several conformation traits. Except for rotation in the hock, all these traits were related to body size or type of horse. This QTL region was previously shown to be associated with several conformation traits in Franches-Montagnes horses [29, 34] and with a principal component reflecting body size in Tennessee Walking horses [50]. In addition, the SNP that showed the strongest association with withers height in several studies (BIEC2_808543) was also associated with several morphometric traits in Thoroughbreds and Spanish purebred horses [27, 28]. In French trotters, Ricard et al*.* [38] identified the two QTL that we found for withers height for a morphometric trait reflecting overall size, not only the QTL on ECA3 but also that on ECA1.

In addition to the shared QTL on ECA3, we identified a number of other QTL that were unique to one or two conformation traits. To date, only a few GWAS on equine conformation have been performed, most of which were based on SNP array data and included a markedly smaller number of traits and horses [6, 29, 30]. To the best of our knowledge, only one GWAS based on imputed sequence-level genotypes has been conducted for conformation traits in horses [9]. However, the number of imputed markers used was considerably smaller than in our study (4 vs. 13 million variants). Compared to the previous studies, we were able to identify QTL for a larger number of the investigated traits, which were generally more pronounced (several QTL detected in the previous studies were only suggestively significant [6, 9, 30]). These results suggest that, in addition to a larger sample size, a larger number of markers to be tested for association can contribute to an improved QTL detection in GWAS, as has been shown in cattle [16]. However, when comparing the results of these studies, it is also necessary to consider the differences in trait definition and recording.

Estimates of genetic correlations between the 13 conformation traits that were associated with the locus on ECA3 and of these traits with withers height varied considerably. However, when only the SNPs located in the QTL region on ECA3 were considered, two clear clusters of highly positively correlated traits could be observed, with strong negative genetic correlations between these two clusters. Hence, all traits that had an association signal on ECA3 showed strong local genetic correlations for the QTL on ECA3, which in several cases were masked by reverse or considerably weaker genetic correlations for the other chromosomes.

GWAS results and genetic correlation estimates suggest that the QTL for withers height on ECA3 influences simultaneously several other conformation traits. However, whether we must assume real pleiotropic effects at this locus or whether all traits simply represent various aspects of stature, is subject to discussion. Another possibility is that the size of an animal biases phenotyping for other conformation traits, which is supported by the fact that almost all traits with a peak on ECA3 are related to body size or type of horse. Hence, one could consider reducing the number of recorded traits associated with body size and preferably focus on the direct measurement of a few clearly defined and objectively ascertainable traits. By improving objectivity and repeatability, this approach could increase the heritability of the recorded traits and improve QTL discovery by GWAS.

### The use of imputed data

Many of the analyses in the present study are based on imputed data. By increasing the number of markers, genotype imputation can boost the power of GWAS and facilitate fine-mapping of causal variants [19]. However, inaccurate imputation can affect the results of GWAS based on imputed data [17]. The accuracy of imputing the warmblood horse variants included in our study was estimated to be rather low, i.e., 0.66 [55]. The main reason for this low accuracy is a shortage of WGS data from horses in general and from specific breeds, preventing the establishment of a larger haplotype reference panel, which could make imputation in horses more accurate. In several studies, the size and composition of the reference panel have been shown to be an important factor influencing the accuracy of imputation (e.g., [84, 85]).

Working with the available resources, the resulting rather low accuracy of imputation should be taken into account when interpreting the results of subsequent analyses. One way to consider imputation errors is to filter the variants based on their imputation accuracy before performing follow-up analyses [17]. Although we did not explicitly filter for Beagle DR2 values before GWAS, we indirectly excluded many variants with a low imputation accuracy by requiring a minimum MAF, as rare variants tend to be imputed with a lower accuracy [86, 87].

Low imputation accuracy affects different types of analyses to varying degrees. Compared to downstream analyses, imputation errors should be less problematic for GWAS, as the purpose of a GWAS is to identify genomic regions associated with traits [11], rather than detecting single causal mutations. When moving on to investigating single variants instead of regions, imputation errors have a more serious impact on the results. In case of the ROH analysis, we considered possible imputation errors by adjusting the parameter settings such that three heterozygous genotypes were allowed in each scanning window, preventing single wrongly imputed heterozygous genotypes to break up a ROH. To investigate putative causal variants for withers height, we Sanger-sequenced the main candidate mutations to validate the imputed genotypes. As the sequencing results for the remaining four top candidate mutations generally matched the imputed genotypes and their DR2 values were reasonably high, we assumed that the imputation accuracy for these variants was sufficiently high for the whole dataset and that the results from GWAS for the SNPs were fairly reliable. Hence, although we tried to reduce the impact of imputation errors in our study, they can represent a potential source of error in the interpretation of the results.

Overall, on the one hand, our study highlights the advantages of using imputed sequence data, such as increased significance levels in GWAS and improved resolution in downstream analyses. On the other hand, it shows that interpreting the results obtained from imputed data requires caution and validation, such as by Sanger-sequencing.

## Conclusions

Applying a large dataset of imputed sequence-level genotypes from German Warmblood horses, we were able to confirm and further characterise a known QTL for withers height on ECA3, including the identification of putative causal variants. Our results suggest that this QTL does not only play an important role for withers height of horses but also influences the manifestation of various other conformation traits related to body size and type of horse. Furthermore, we discovered a number of novel QTL associated with various conformation traits and estimated their genetic parameters based on marker data. This study highlights the suitability and benefits of using imputed sequence data for QTL detection and fine mapping of causal variants, although it also shows that the results have to be handled with caution.

### Supplementary Information

**Additional file 1: Figure S1.** Population structure of the horses included in the study. Multidimensional scaling plot showing the population structure of all horses with available genotype data after filtering and quality control (n = 4972). The different colours represent the different breeding associations Holstein (HOL), Oldenburg (OL), Oldenburg International (OS), Trakehner (TRAK), and Westfalian (WESTF). Multidimensional scaling was performed with PLINK 1.9 using medium-density SNP array data (61,599 variants).

**Additional file 2: Table S1.** Descriptive statistics and variance component estimates for withers height and 81 conformation traits in German Warmblood horses. Trait description and descriptive statistics for all conformation traits considered in the study. Descriptive statistics include the sample size (N) of horses with genotype and phenotype data for the respective trait, minimum (Min), maximum (Max), mean and standard deviation (SD). Furthermore, variance component estimates are presented for all conformation traits: genetic variance (V_G_), residual variance (V_E_), phenotypic variance (V_P_) and ratio of genetic to phenotypic variance (heritability, h^2^) with the respective standard errors (SE), and the *p*-value (*p*) for the genetic variance calculated from the log-likelihood ratio test (LRT). Traits with a *p*-value larger than 0.05 were excluded from further analyses (highlighted in grey).

**Additional file 3: Table S2.** PCR and sequencing primers. Primers used for amplifying and sequencing the target DNA segments surrounding putative causal variants for withers height in German Warmblood horses.

**Additional file 4: Figure S2.** Quantile–quantile plots for the preliminary and conditional GWAS for withers height. The observed *p*-values (black) are plotted against the expected *p*-values (red) and have a genomic inflation factor of λ.

**Additional file 5: Table S3.** Quantitative trait loci (QTL) identified in genome-wide association studies for 61 conformation traits in 4768 to 4891 horses (depending on the trait). This table presents the *Equus caballus* (ECA) chromosome and the position of the QTL, the number of SNPs genome-wide significantly associated with the respective trait at the given locus (number of SNPs) and the *p*-value of the most significantly associated SNP at the locus (lowest *p*-value).

**Additional file 6: Figure S3.** Results of the genome-wide association studies for 61 conformation traits in 4768 to 4891 horses (depending on the trait). Manhattan plots of the –log_10_ *p*-values for the association of variants with the respective trait. The dark red horizontal line indicates the genome-wide significance threshold with α = 0.05 and Bonferroni correction for multiple testing (*p* = 3.8 × 10^–9^). Due to computational limitations, variants with a *p*-value > 0.05 were excluded from the plots. In addition to the Manhattan plots (left-hand side), the respective quantile–quantile plots for the traits are given (right-hand side). The observed *p*-values (black) are plotted against the expected *p*-values (red) and have a genomic inflation factor of λ (stated below the plot).

**Additional file 7: Table S4.** Top associated SNPs for withers height and 13 conformation traits with a significant association signal on chromosome 3. This table includes the positions of the top SNPs on EquCab3.0, the respective alleles and frequencies, the SNP effect (beta), standard error (SE) and *p*-value (*p*). SNPs highlighted in bold were included in conditional GWAS for the respective conformation trait.

**Additional file 8: Figure S4.** Results of the conditional genome-wide association studies in 4769 to 4891 horses (depending on the trait) for 13 conformation traits showing a peak on chromosome 3 next to the *LCORL/NCAPG*-locus. Manhattan plots of the –log_10_ *p*-values for the association of variants with withers height. The dark red horizontal line indicates the genome-wide significance threshold with α = 0.05 and Bonferroni correction for multiple testing (*p* = 3.8 × 10^–9^). Due to computational limitations, variants with a *p*-value > 0.05 were excluded from the plots. Either the top associated SNP from the GWAS for the respective conformation trait itself, the top associated SNP from the GWAS for withers height, or withers height itself was included in the initial model as an additional fixed effect. In addition to the Manhattan plots (left-hand side), the respective quantile–quantile plots for the traits are given (right-hand side). The observed *p*-values (black) are plotted against the expected *p*-values (red) and have a genomic inflation factor of λ (stated below the plot).

**Additional file 9: Table S5.** Genetic correlations of 61 conformation traits with withers height. Genetic correlations (r_G_) and standard errors (SE) were estimated from the data of 4768 German Warmblood horses (for the combination of two conformation traits) or 2938 horses (for the combination of one conformation trait and withers height). In genome-wide association studies, all traits showed a significant association signal in the same region on *Equus caballus* chromosome 3 (ECA3). Genetic correlations were estimated for the markers on all autosomes on the one hand, and separately for the markers in the QTL region on ECA3 and those in the rest of the genome on the other hand. **Table S6.** Genetic correlations of 13 conformation traits with withers height. Genetic correlations (r_G_) and standard errors (SE) were estimated from the data of 4768 German Warmblood horses (for the combination of two conformation traits) or 2938 horses (for the combination of one conformation trait and withers height). In genome-wide association studies, all traits showed a significant association signal in the same region on *Equus caballus* chromosome 3 (ECA3). Genetic correlations were estimated for the markers on all autosomes on the one hand, and separately for the markers in the QTL region on ECA3 and for those in the rest of the genome on the other hand.

**Additional file 10: Table S7.** Results of the variant effect prediction for withers height in 2709 German Warmblood horses. Only the variants that were statistically significantly associated with withers height in the preliminary genome-wide association study (GWAS) were used as the input. Shown are all the variants that were predicted to have a high or moderate impact, including their dosage R-squared (DR2) value from imputation with Beagle 5.1 and the following information from GWAS: SNP effect (beta), standard error (SE) and *p*-value (*p*). The variants highlighted in grey are located within runs of homozygosity shared by the ten horses with the highest estimated breeding value for withers height (based on the markers on chromosome 3). The variants printed in bold were in high linkage disequilibrium (R^2^ > 0.8) with the top variants identified in GWAS.

**Additional file 11: Table S8.** Linkage disequilibrium structure of putative causal variants for withers height. Variants that were significantly associated with withers height and in high linkage disequilibrium (R^2^ > 0.8) to the top variants identified in a genome-wide association study in German Warmblood horses.

**Additional file 12: Figure S5.** Predicted structures of proteins from genes carrying putative causal variants for withers height. Proteins altered by candidate mutations (red) in comparison to wild-type proteins (blue) for the putative causal variants rs1146838995 (a), rs1148715914 (b), rs1138481672 (c), rs1139684227 (d) and rs1137124154 (e).

## Data Availability

The raw sequence data from all horses included in the reference panel applied for genotype imputation is publicly available from the European Nucleotide Archive (ENA) at EMBL-EBI (https://www.ebi.ac.uk/ena/browser/view) under the accession numbers stated in Additional file 1 in Reich et al*.* [55]. The horse genome assembly used for the analyses was EquCab3.0 (GCA_002863925.1) obtained from Ensembl (ftp://ftp.ensembl.org/pub/release-100/fasta/equus_caballus/dna/Equus_caballus.EquCab3.0.dna.toplevel.fa.gz).
