# Supplementary figures and images for: Genomic analyses of withers height and linear conformation traits in German Warmblood horses using imputed sequence-level genotypes
Source: Genet Sel Evol. 2024 Jun 13;56:45. doi: 10.1186/s12711-024-00914-6 (PMC11177368; doi:10.1186/s12711-024-00914-6)

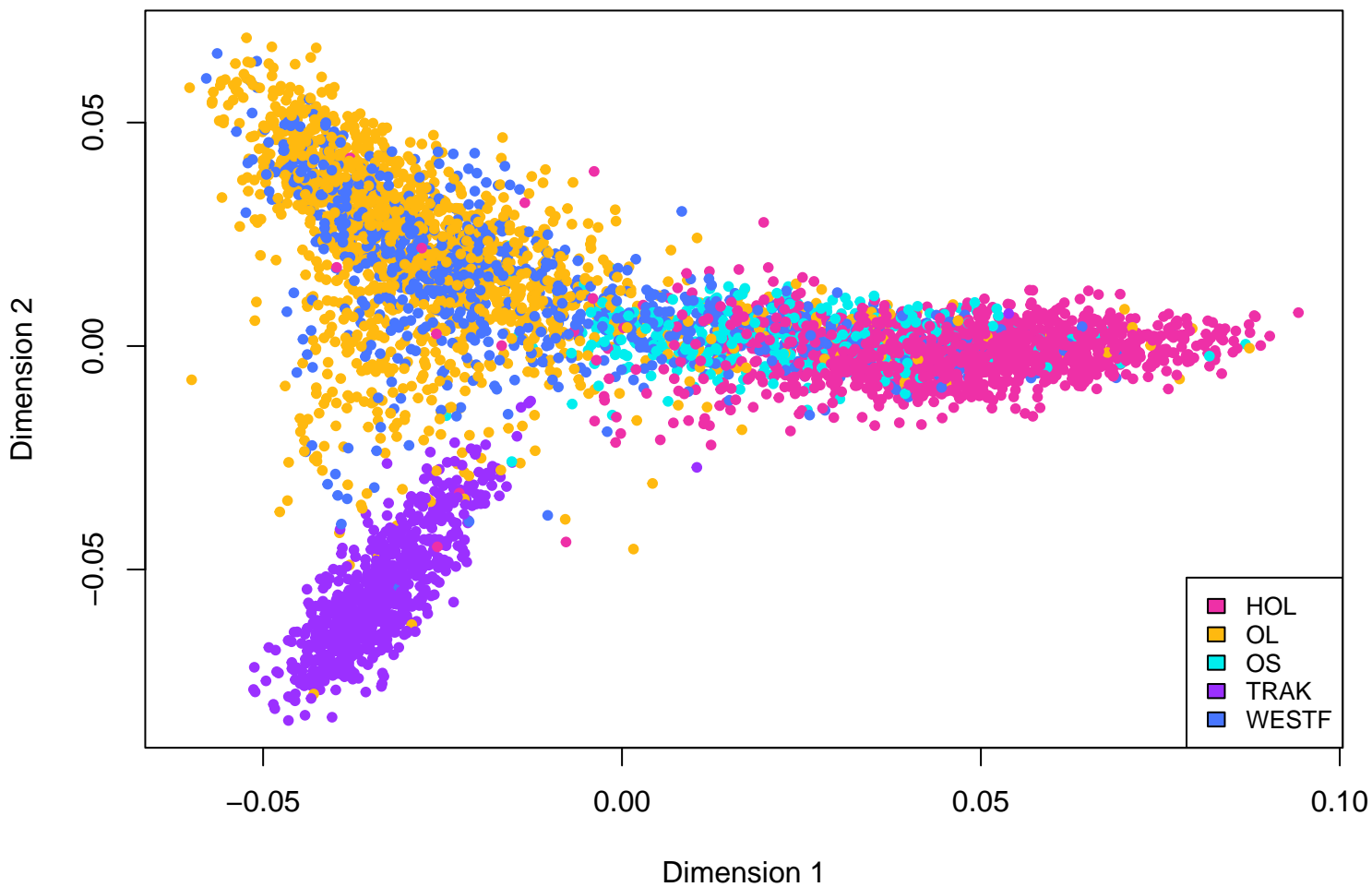

Supplement: Supplementary file 1 — Additional file 1: Figure S1. Population structure of the horses included in the study. Multidimensional scaling plot showing the population structure of all horses with available genotype data after filtering and quality control (n = 4972). The different colours represent the different breeding associations Holstein (HOL), Oldenburg (OL), Oldenburg International (OS), Trakehner (TRAK), and Westfalian (WESTF). Multidimensional scaling was performed with PLINK 1.9 using medium-density SNP array data (61,599 variants). [file 12711_2024_914_MOESM1_ESM.pdf]

**GWAS – Withers height**

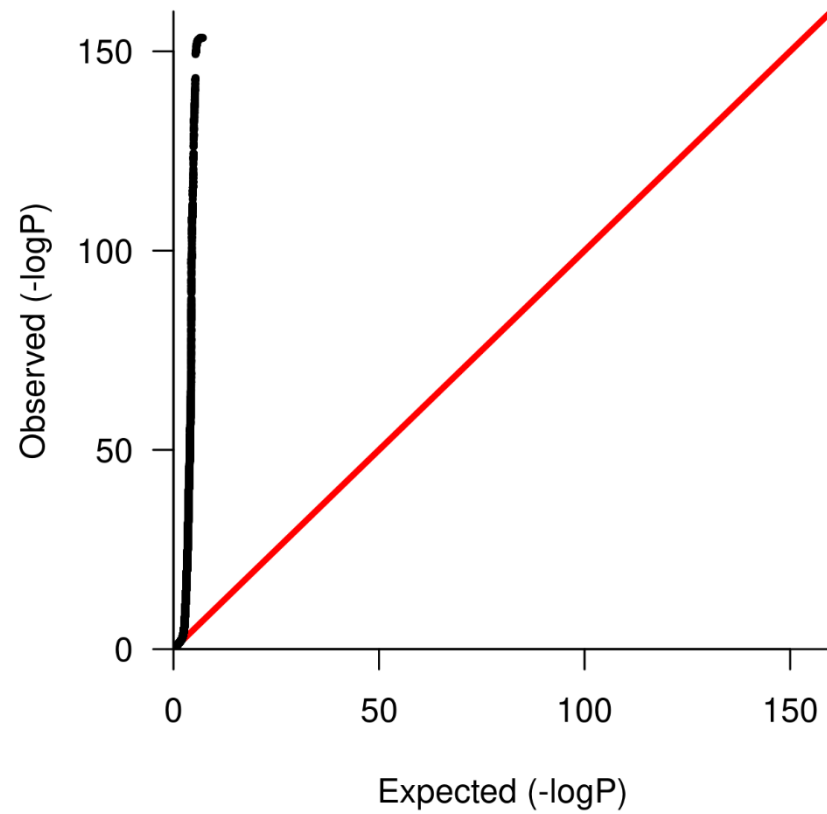

$\lambda = 1.120$

**Conditional GWAS – Withers height**

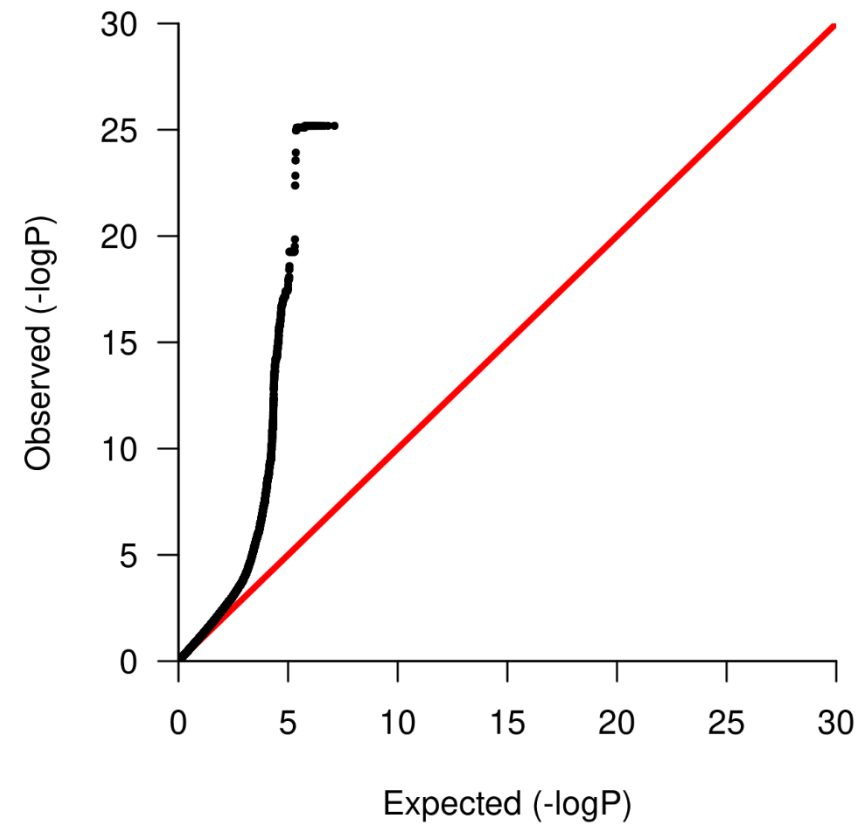

$\lambda = 1.203$

Supplement: Supplementary file 4 — Additional file 4: Figure S2. Quantile–quantile plots for the preliminary and conditional GWAS for withers height. The observed p-values (black) are plotted against the expected p-values (red) and have a genomic inflation factor of λ. [file 12711_2024_914_MOESM4_ESM.pdf]

a

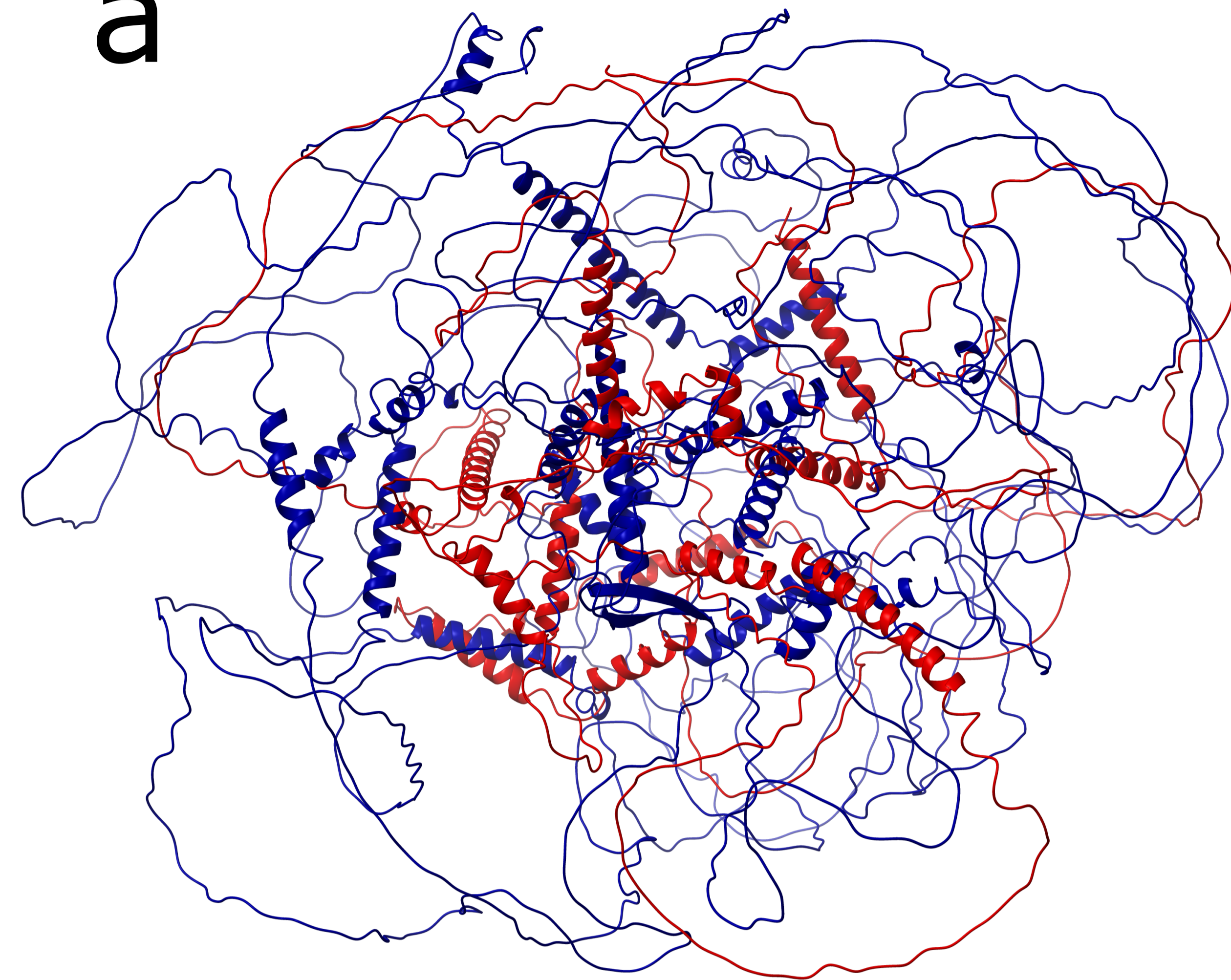

b

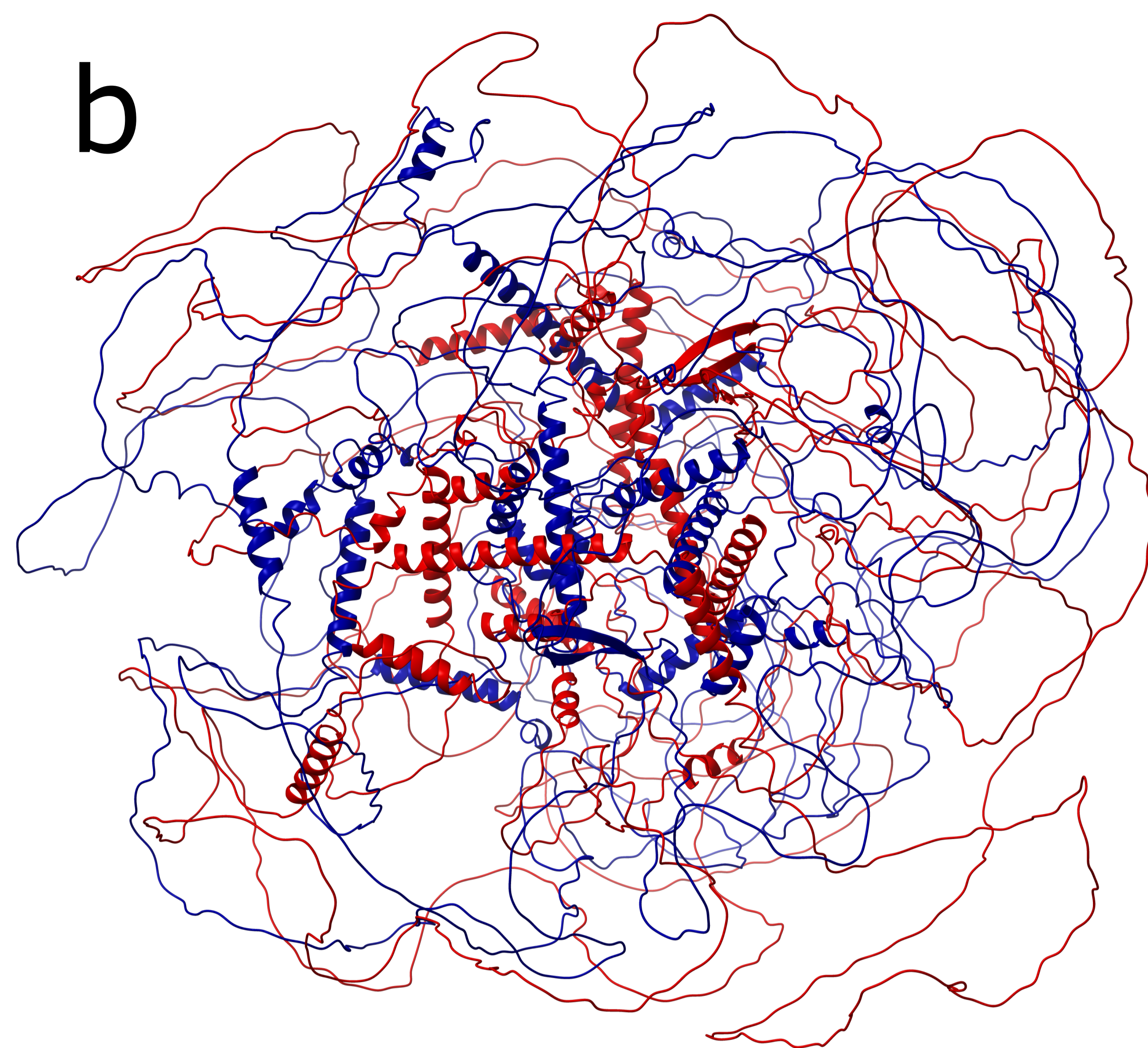

c

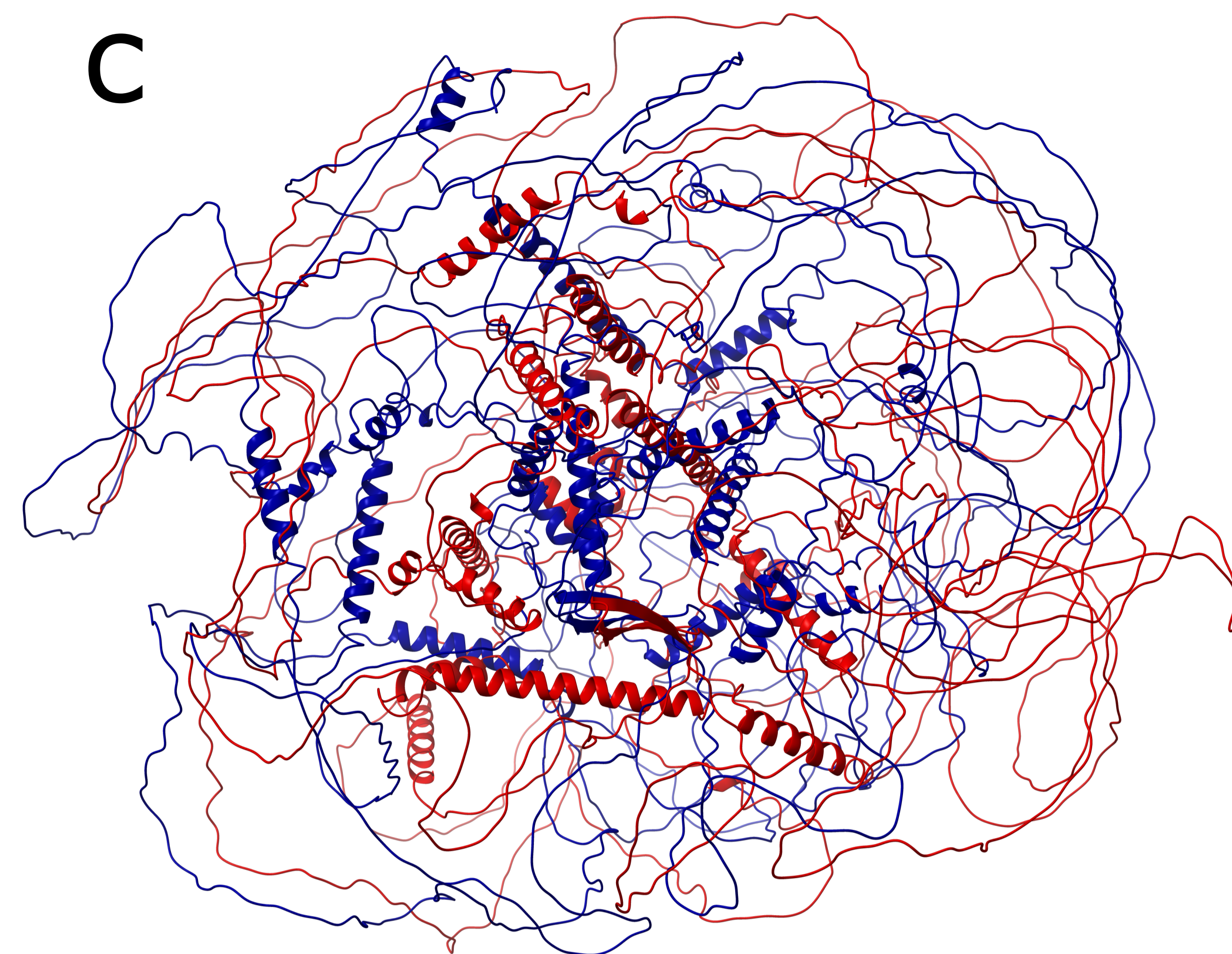

d

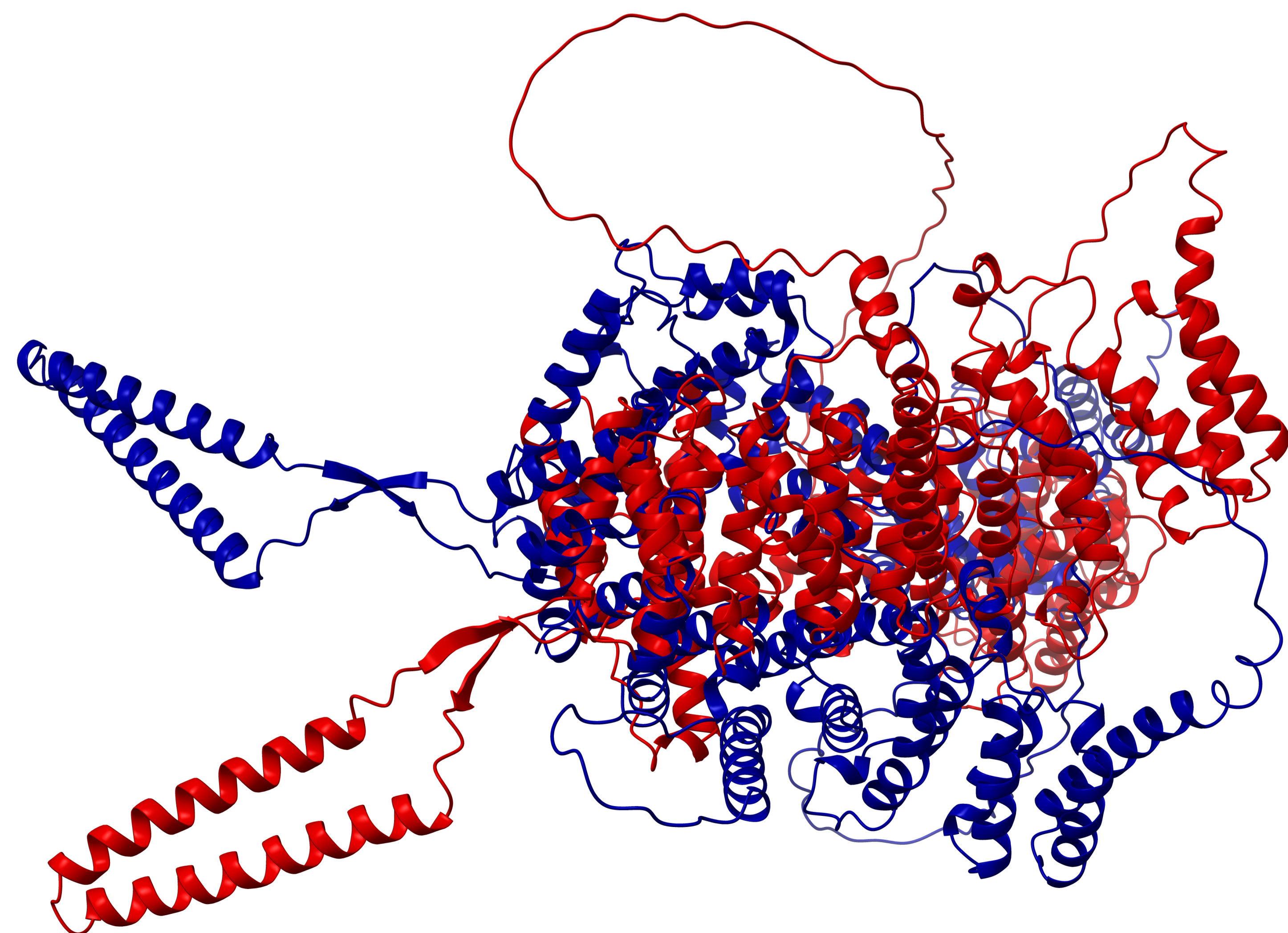

e

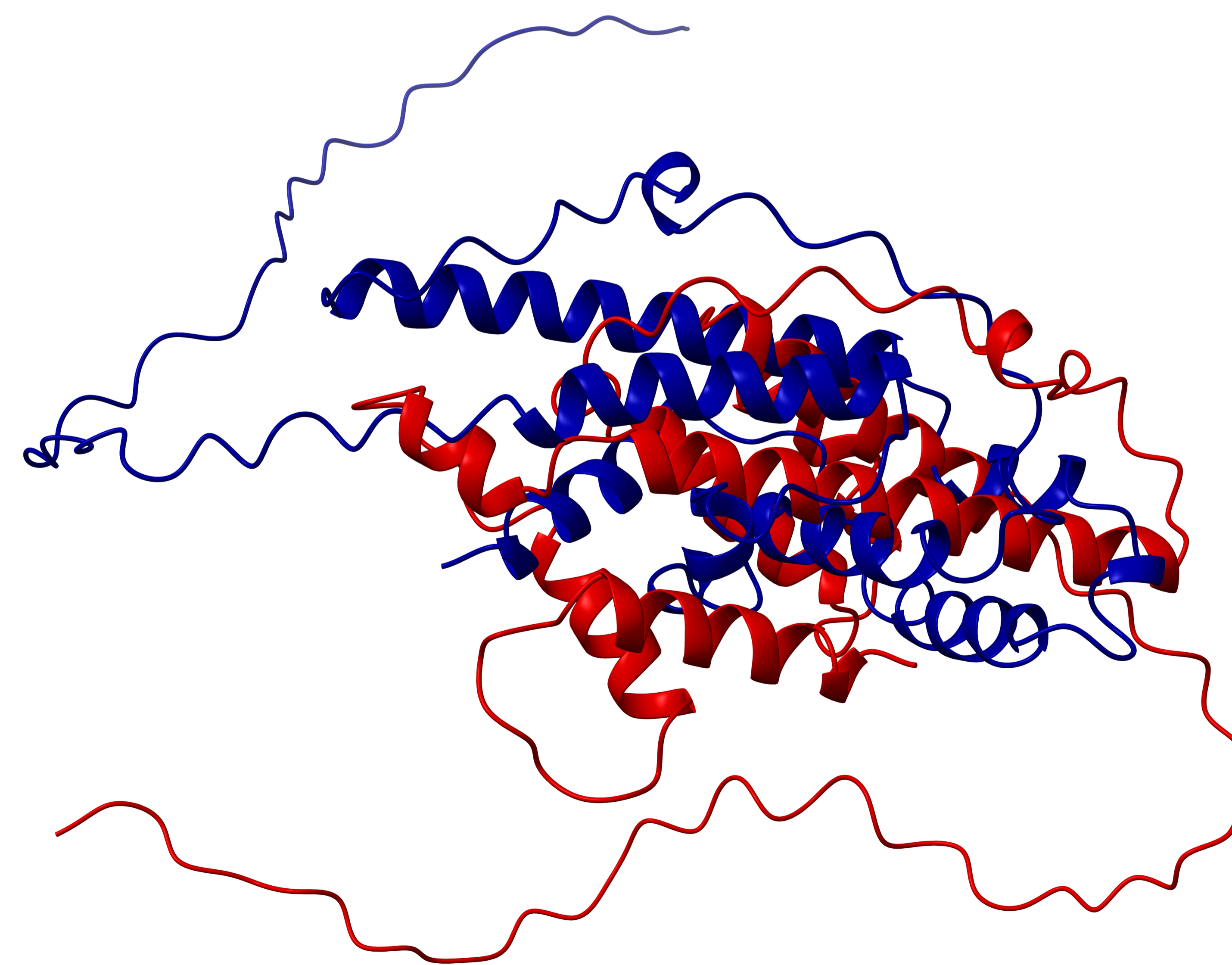

Supplement: Supplementary file 12 — Additional file 12: Figure S5. Predicted structures of proteins from genes carrying putative causal variants for withers height. Proteins altered by candidate mutations (red) in comparison to wild-type proteins (blue) for the putative causal variants rs1146838995 (a), rs1148715914 (b), rs1138481672 (c), rs1139684227 (d) and rs1137124154 (e). [file 12711_2024_914_MOESM12_ESM.pdf]
