# Supplementary material for: Genomic analyses of withers height and linear conformation traits in German Warmblood horses using imputed sequence-level genotypes
Source: Genet Sel Evol. 2024 Jun 13;56:45. doi: 10.1186/s12711-024-00914-6 (PMC11177368; doi:10.1186/s12711-024-00914-6)
Supplement: Supplementary file 6 — Additional file 6: Figure S3. Results of the genome-wide association studies for 61 conformation traits in 4768 to 4891 horses (depending on the trait). Manhattan plots of the –log10 p-values for the association of variants with the respective trait. The dark red horizontal line indicates the genome-wide significance threshold with α = 0.05 and Bonferroni correction for multiple testing (p = 3.8 × 10^–9^). Due to computational limitations, variants with a p-value > 0.05 were excluded from the plots. In addition to the Manhattan plots (left-hand side), the respective quantile–quantile plots for the traits are given (right-hand side). The observed p-values (black) are plotted against the expected p-values (red) and have a genomic inflation factor of λ (stated below the plot). [file 12711_2024_914_MOESM6_ESM.pdf]

Breed type [plain - true to type]

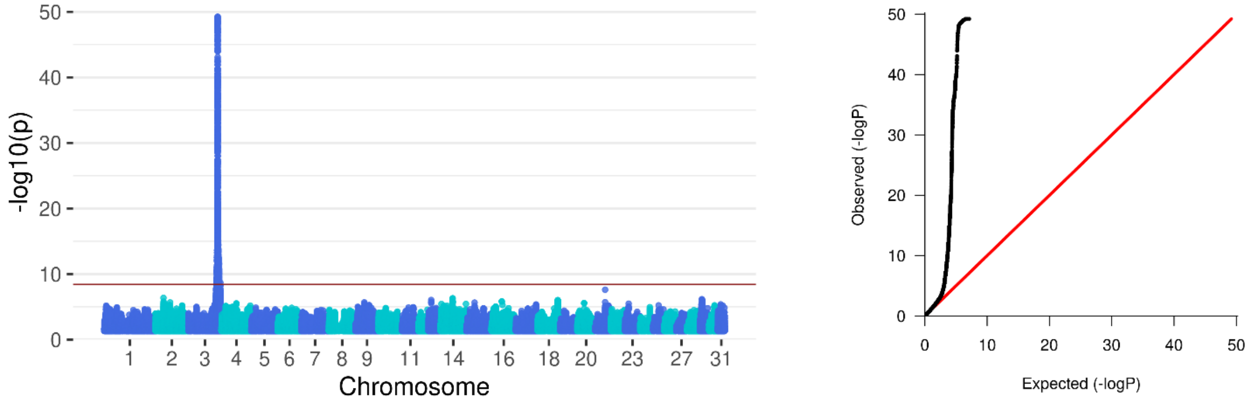

λ = 1.133

Gender expression [weak - strong]

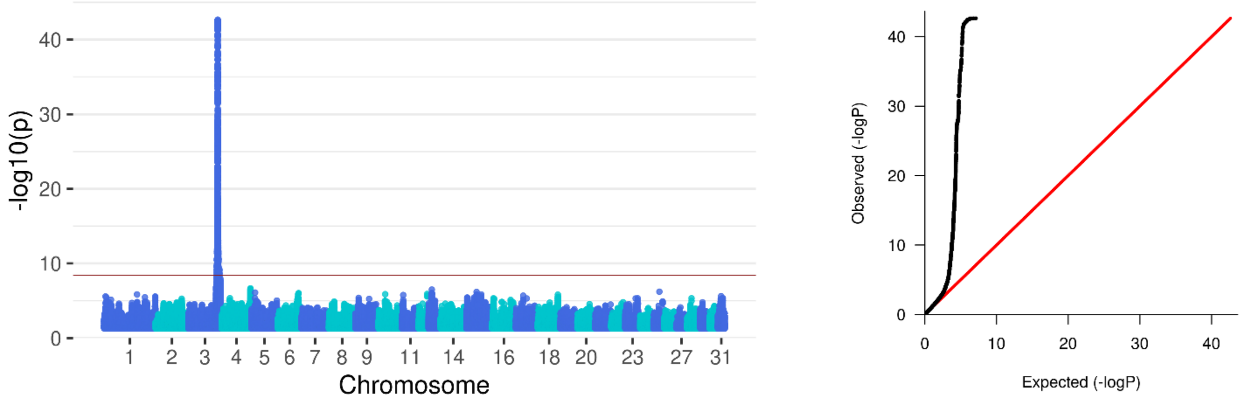

λ = 1.115

Frame [small-framed - large-framed]

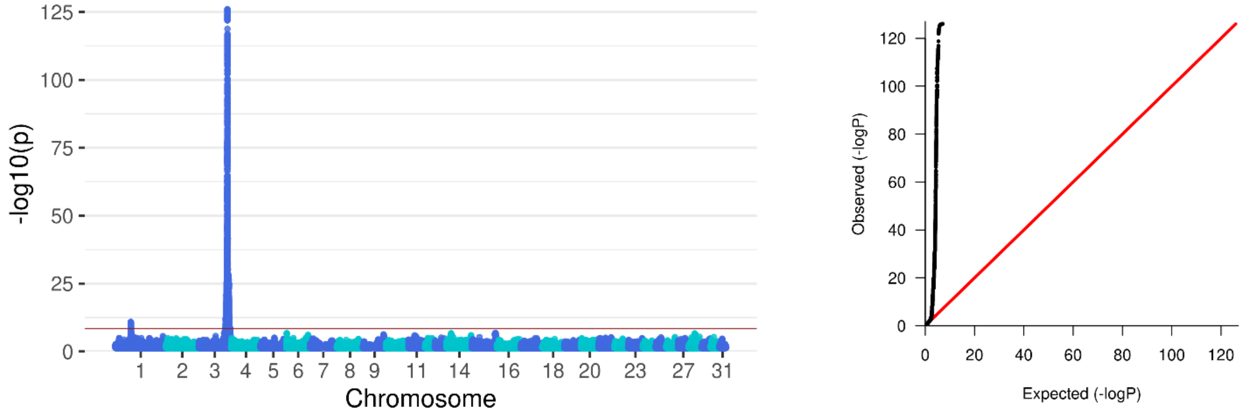

λ = 1.083

Caliber [light - heavy]

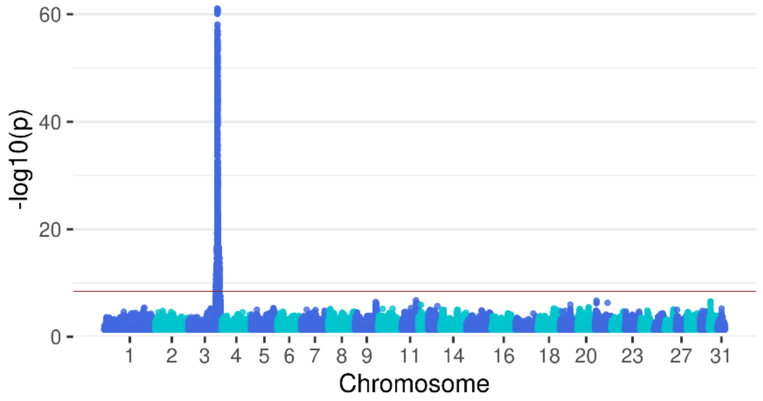

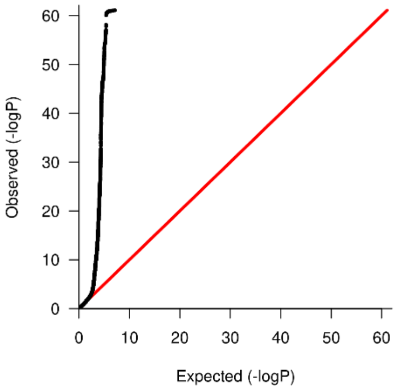

λ = 1.018

Chest width [narrow - wide]

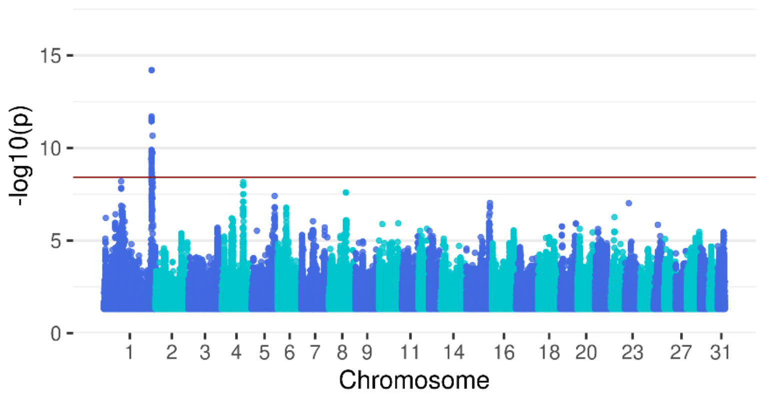

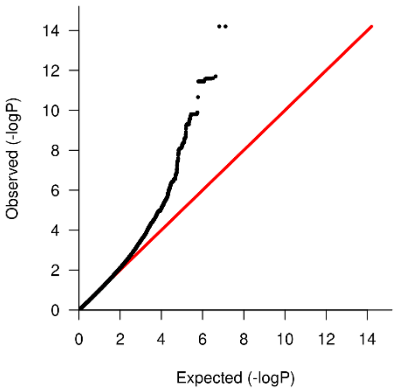

λ = 0.945

Barrel [shallow (tucked-up) - deep]

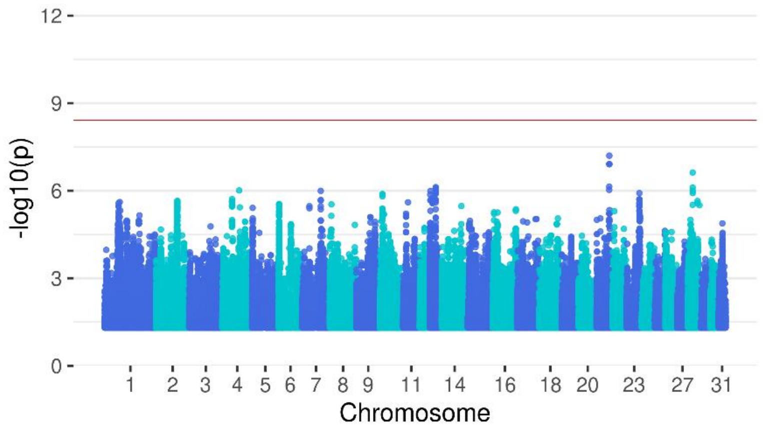

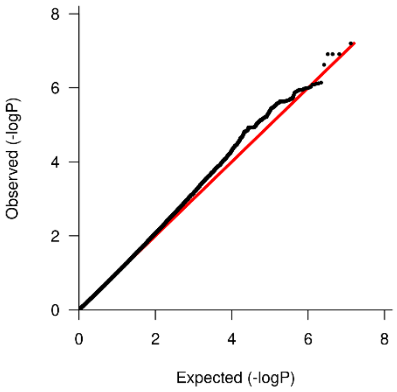

λ = 0.983

Condition [skinny - fat]

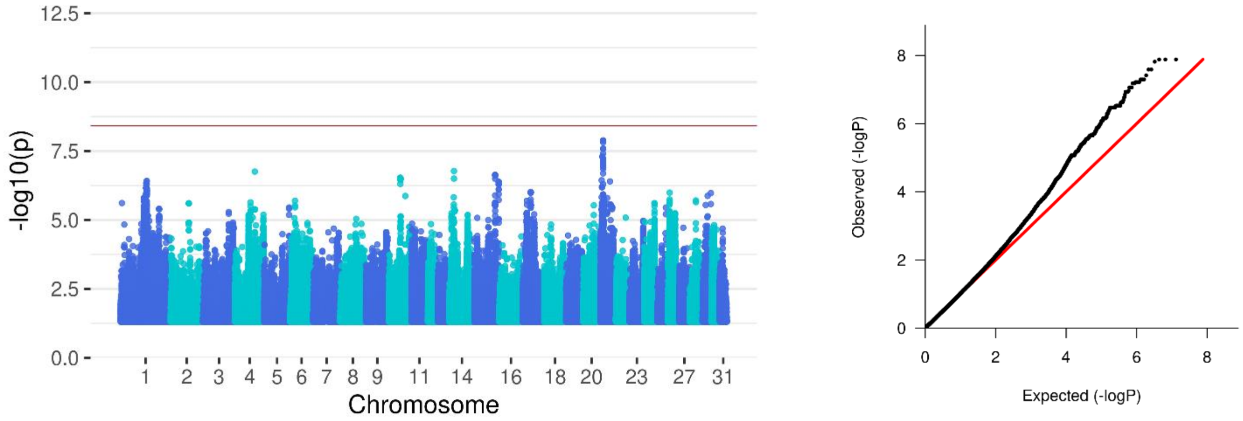

λ = 1.001

Development [poor - much]

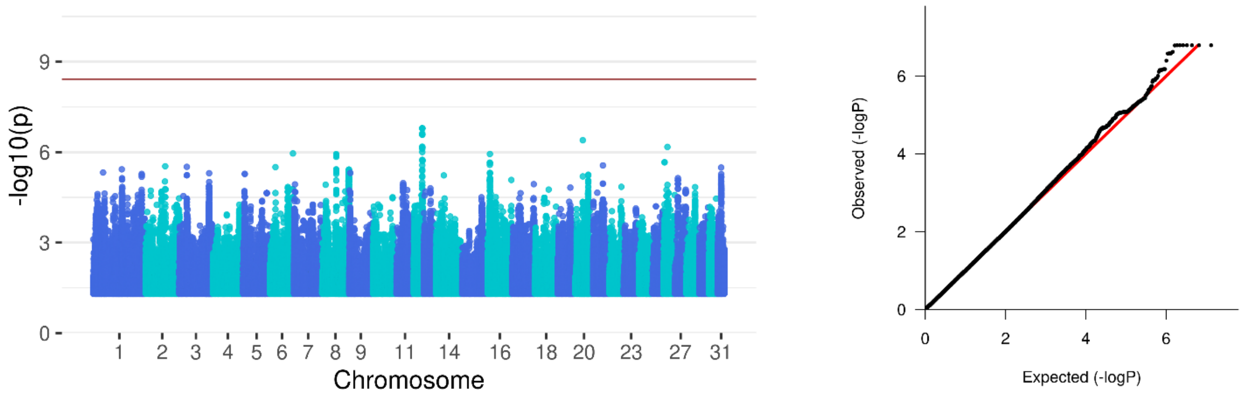

λ = 0.994

Length of legs [short-legged - long-legged]

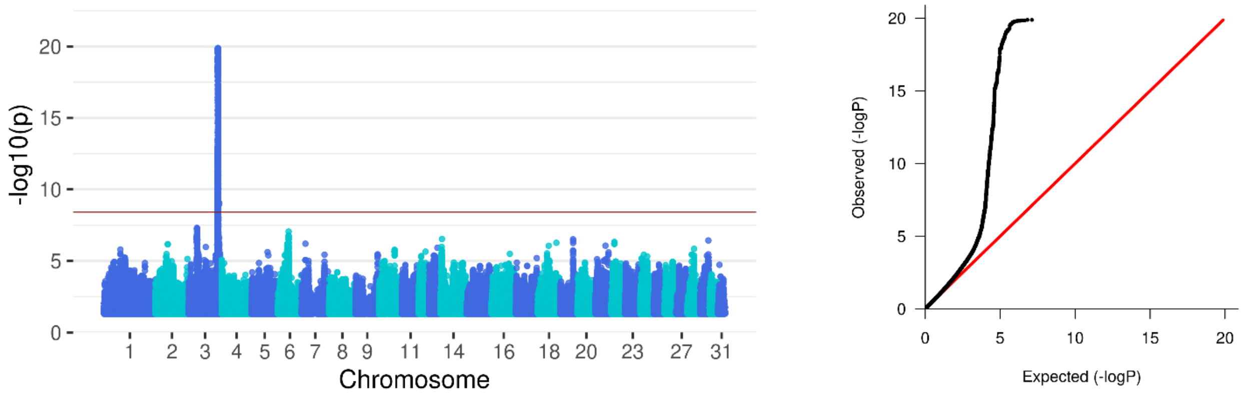

λ = 1.061

Harmony of proportions [unharmonious - harmonious]

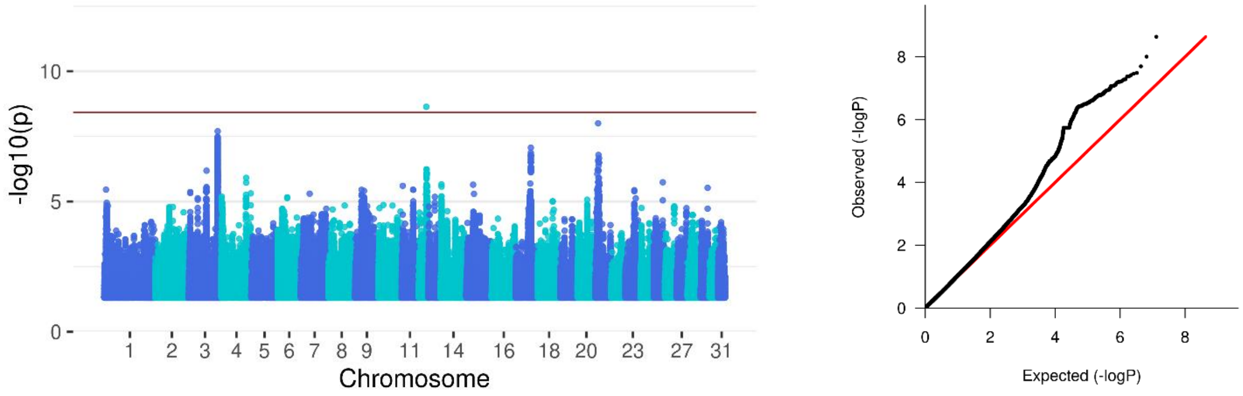

λ = 1.024

Body shape [square - (long-)rectangular]

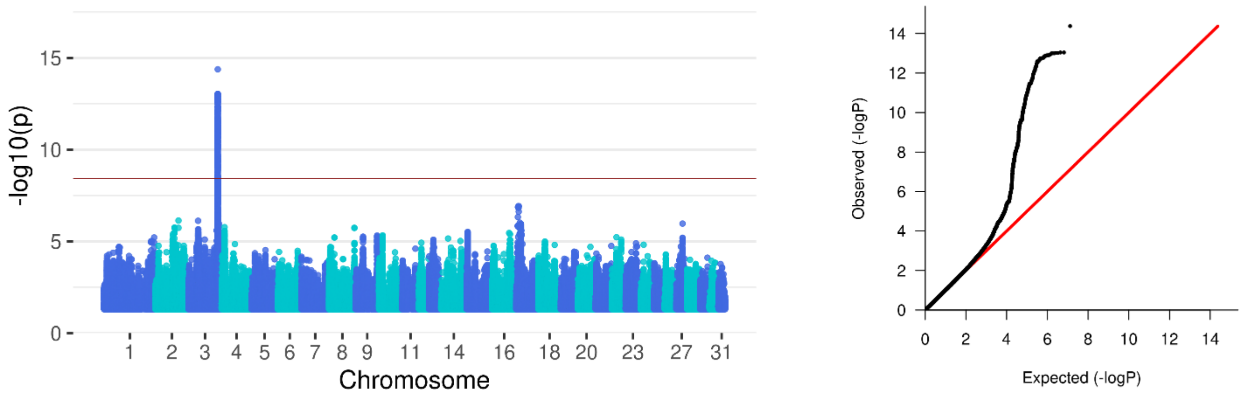

λ = 0.996

Body direction [downhill - uphill]

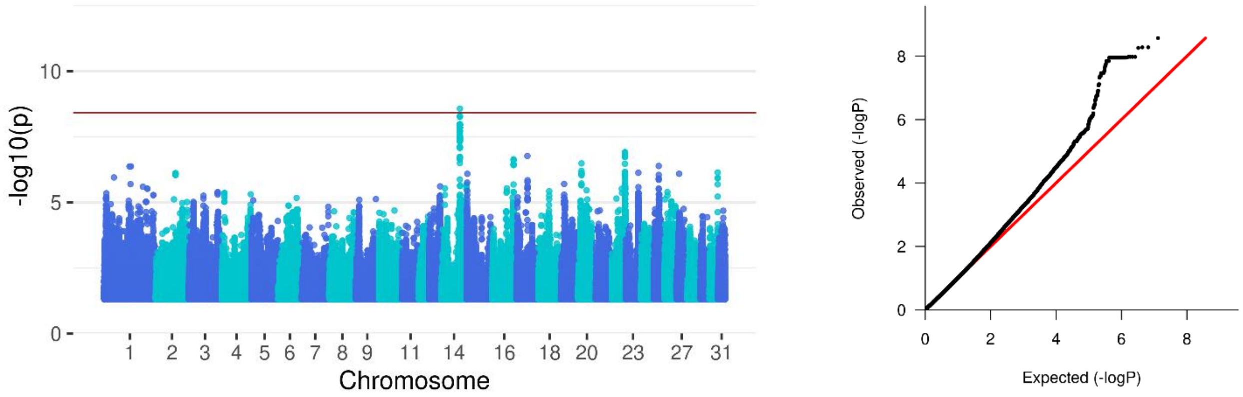

λ = 0.989

Head shape [coarse - fine]

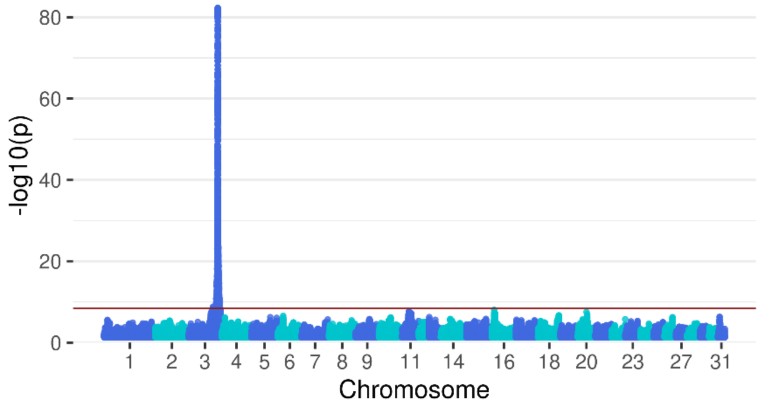

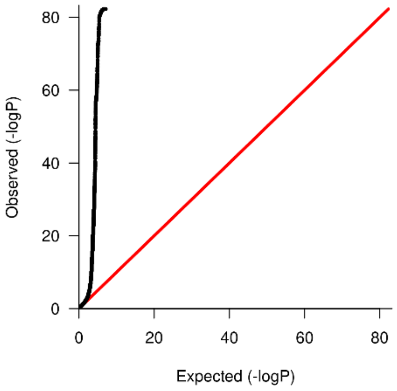

λ = 1.181

Head length [short - long]

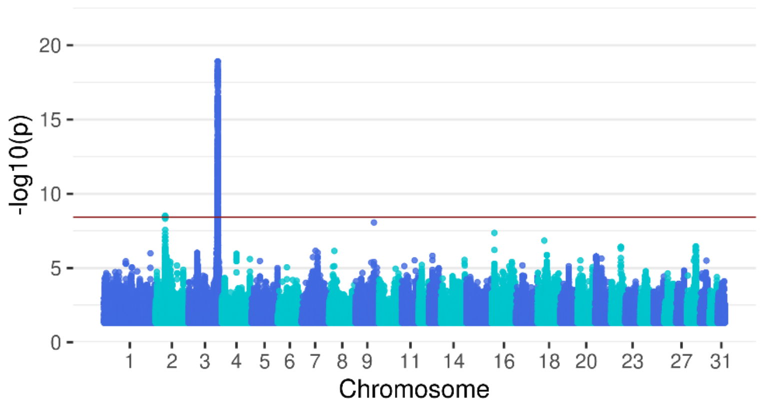

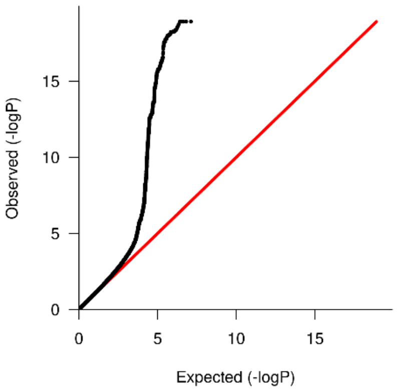

λ = 1.035

Eye size [small - large]

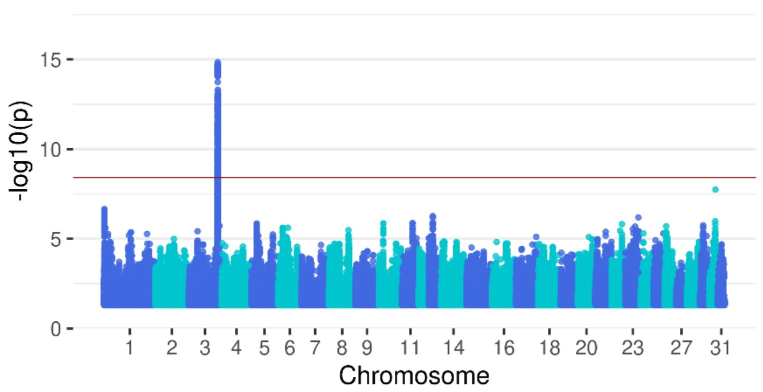

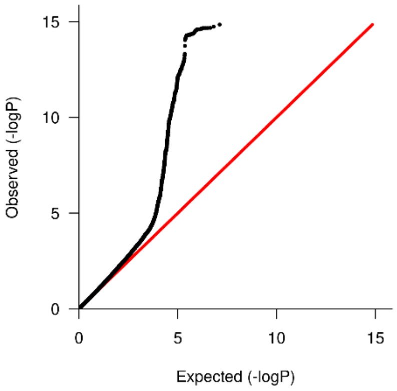

λ = 1.057

Eye colour [white in the eye]

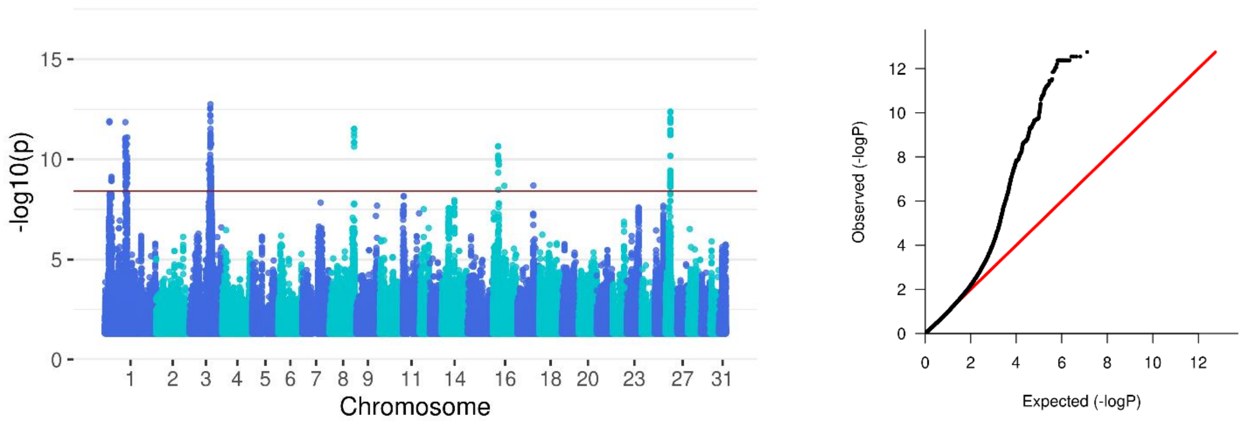

λ = 0.932

Head-neck connection [heavy - light]

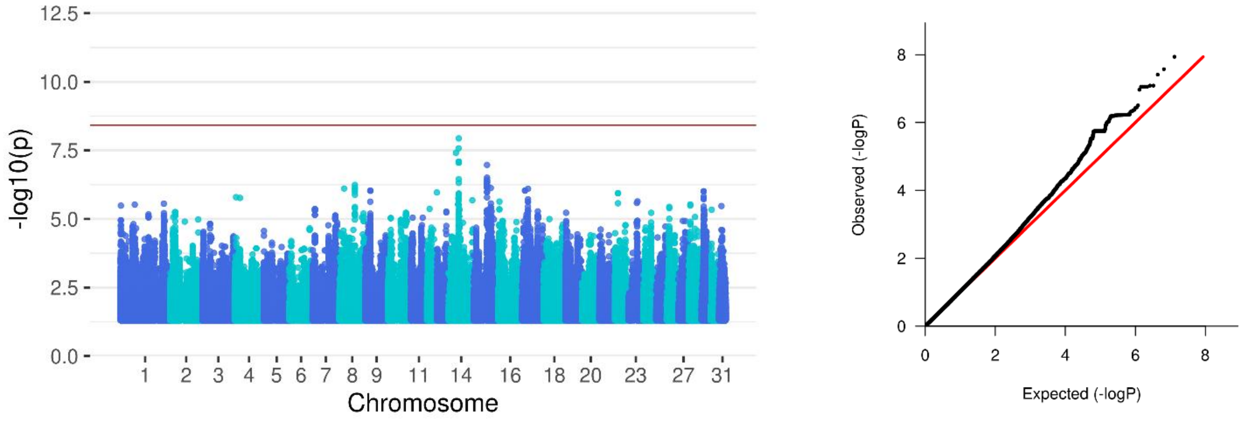

λ = 1.009

Cheeks (jowl) [heavy - light]

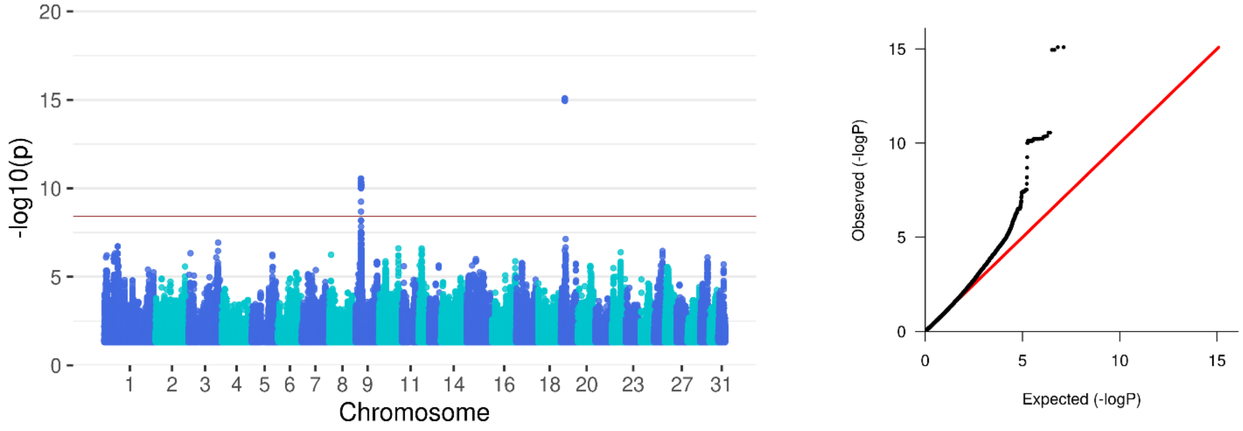

λ = 0.978

Length of neck [short - long]

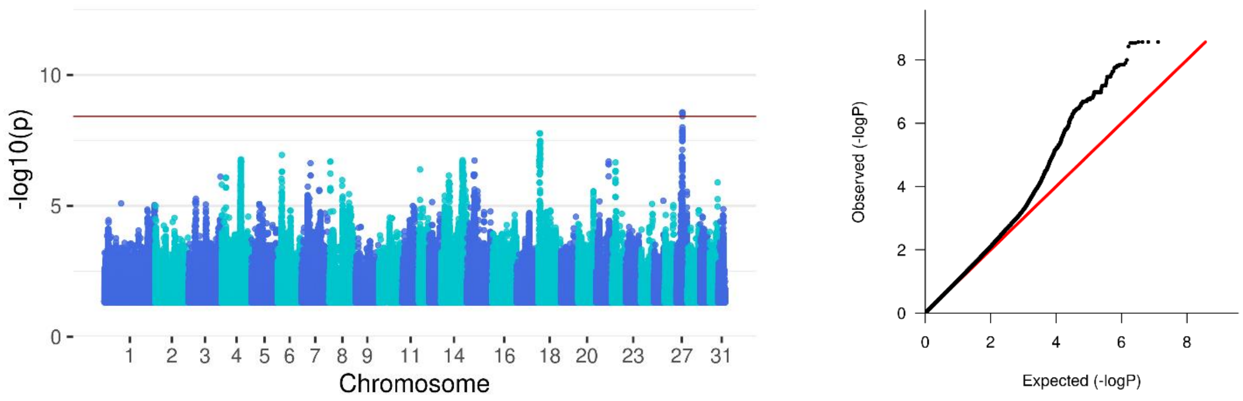

λ = 1.041

Set of neck [low - high]

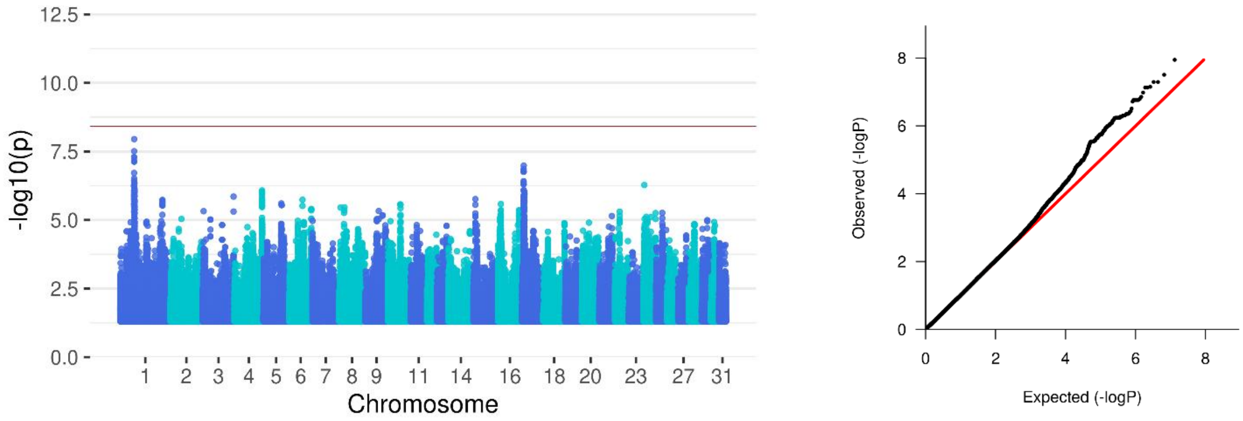

λ = 1.002

Muscling area of neck [ewe-necked - top line dominated neck]

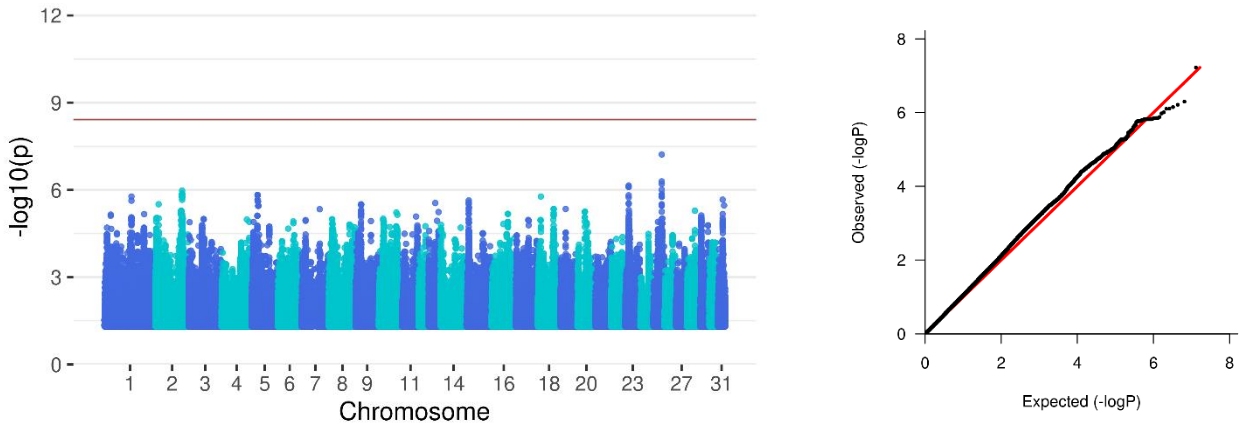

λ = 1.054

Shape of neck [straight - arched]

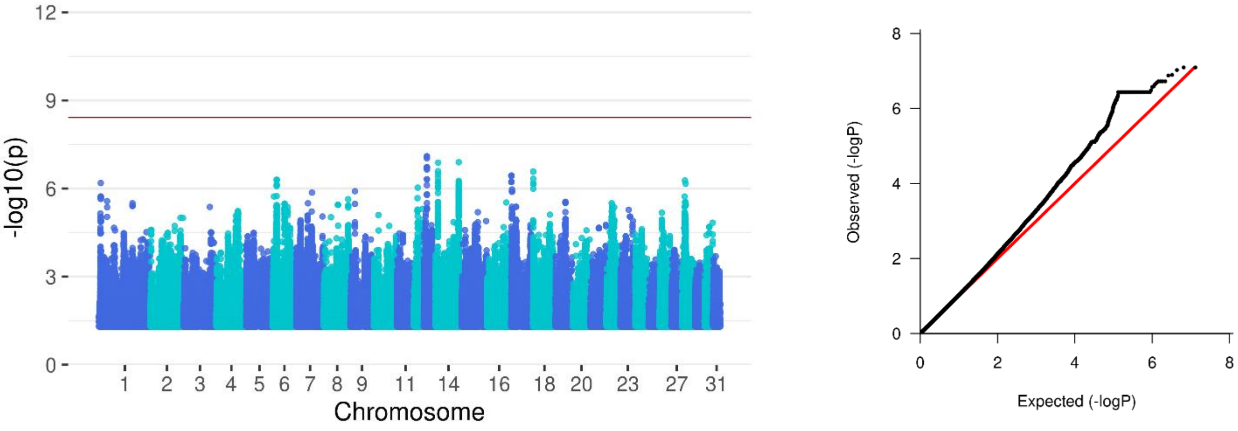

λ = 1.013

Shape of neck [thin - thick]

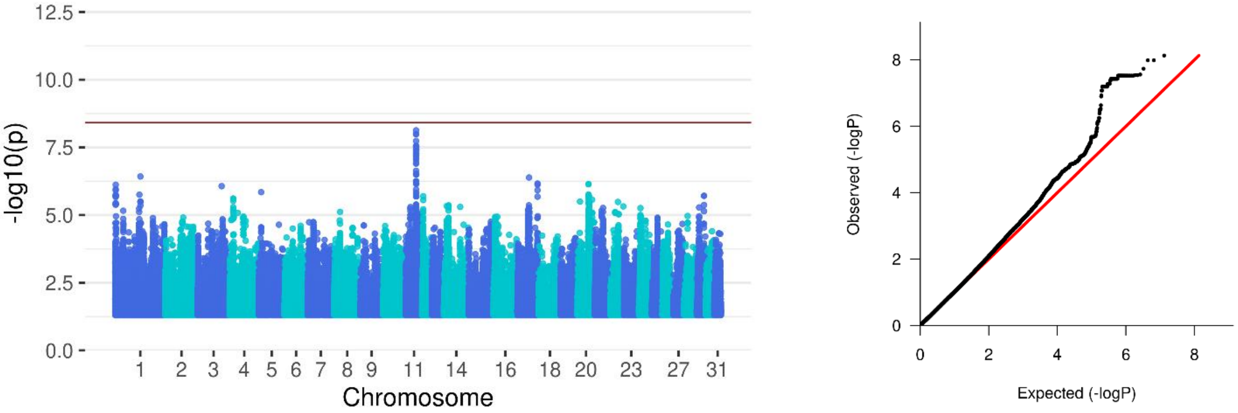

λ = 1.017

Neck connection to withers [marked notch/dip]

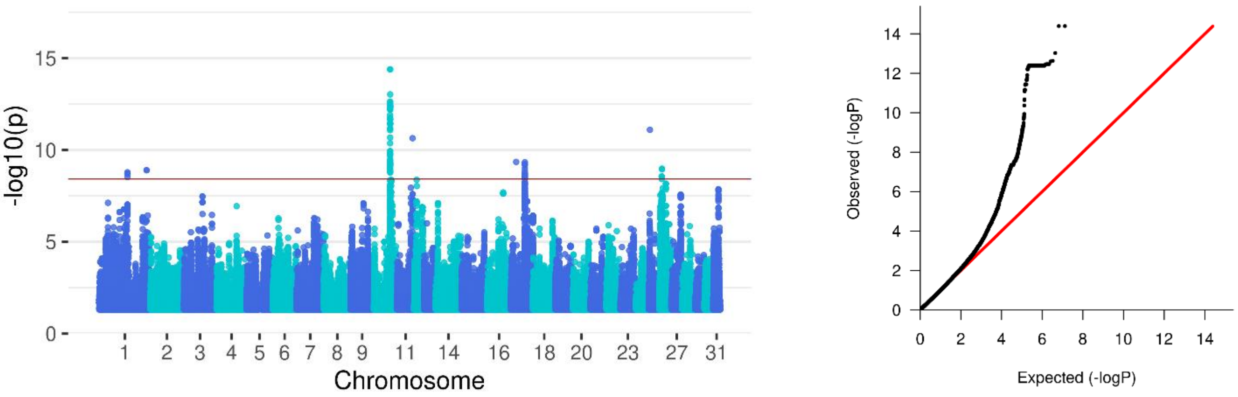

λ = 0.984

Length of withers [short - long]

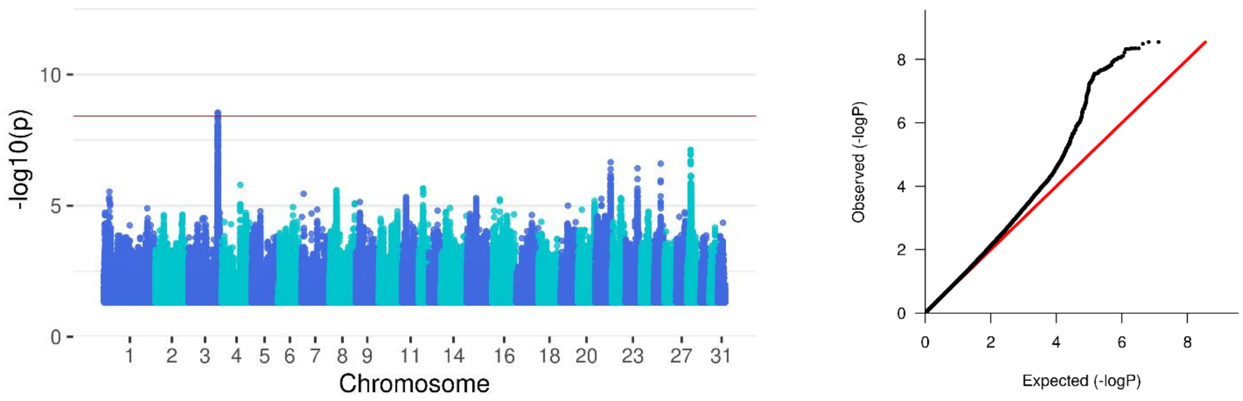

λ = 1.046

Height of withers [flat - high]

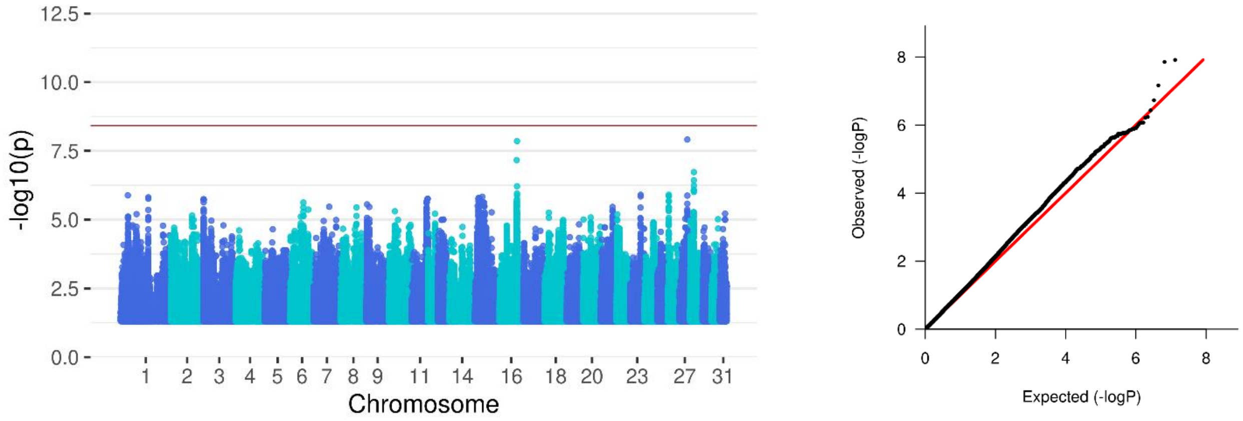

λ = 1.062

Length of shoulder [short - long]

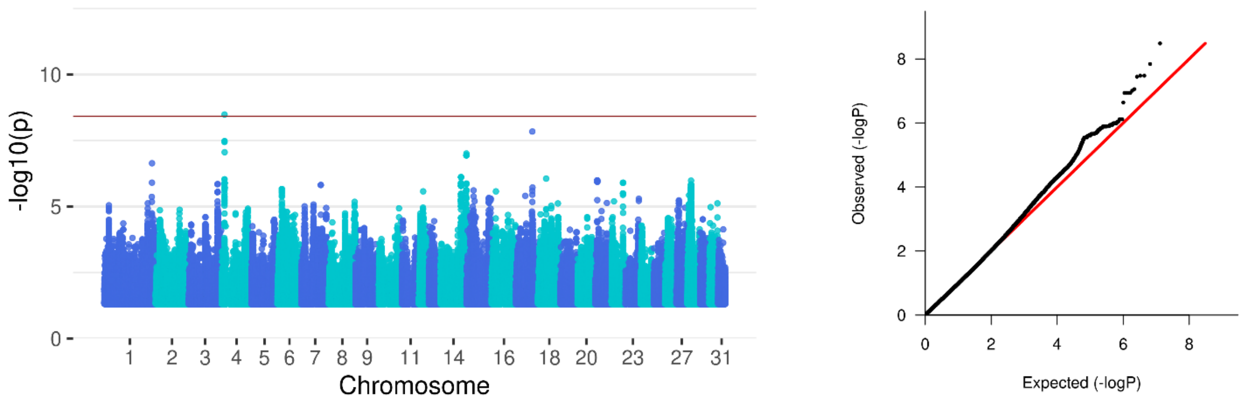

λ = 0.974

Shoulder position [clearly pushed forward]

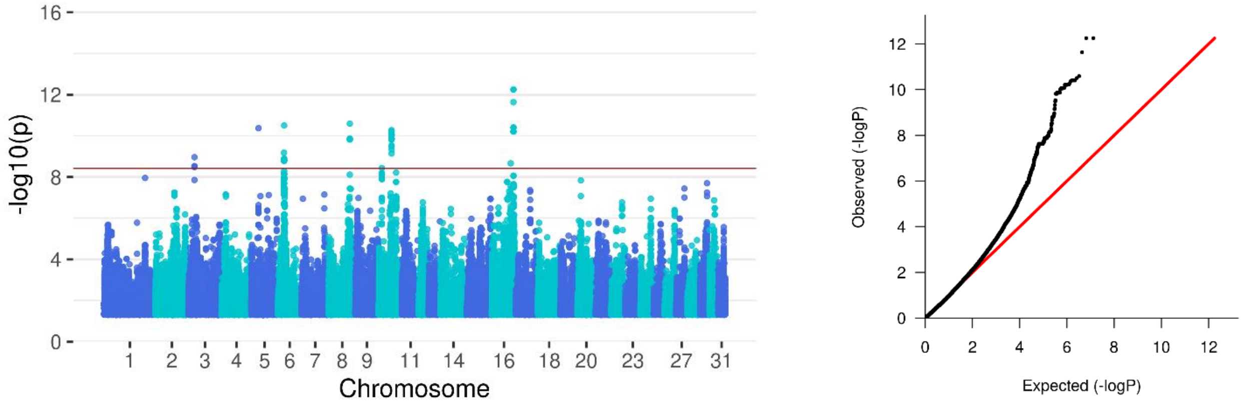

λ = 0.913

Course of topline [disturbed - straight]

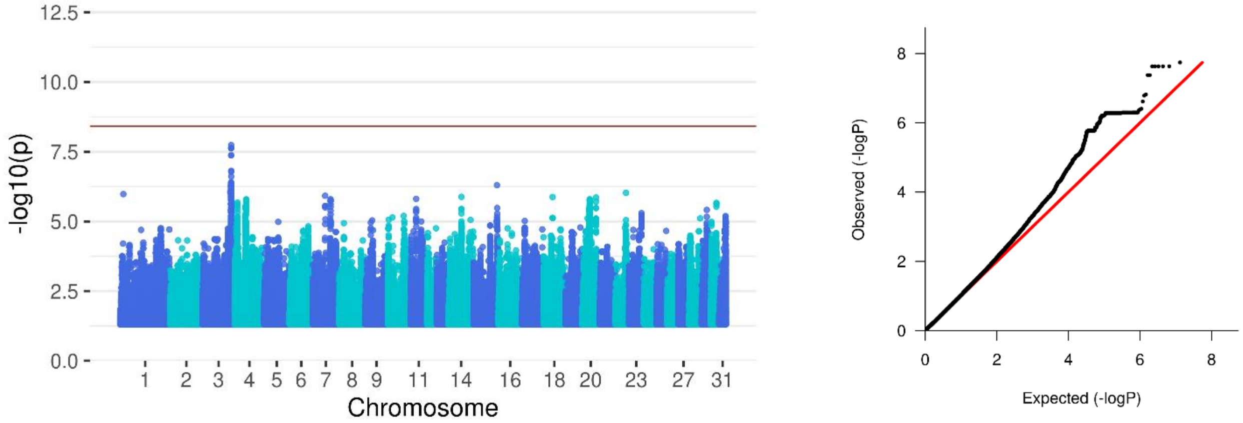

λ = 1.014

Length of back [short - long]

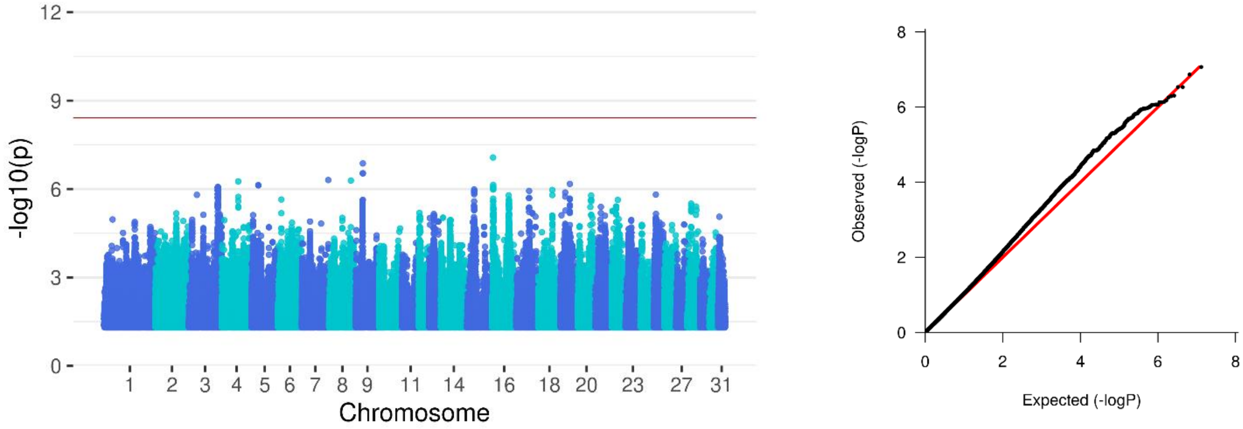

λ = 1.031

Line (strength) of back [dipped - roached]

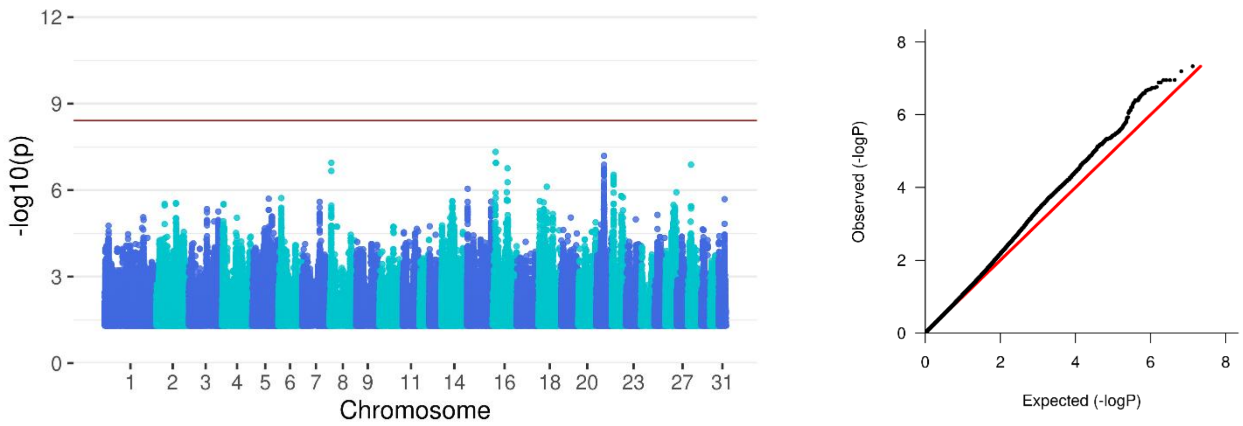

λ = 1.055

Line (strength) of loins [dipped (weak) - roached]

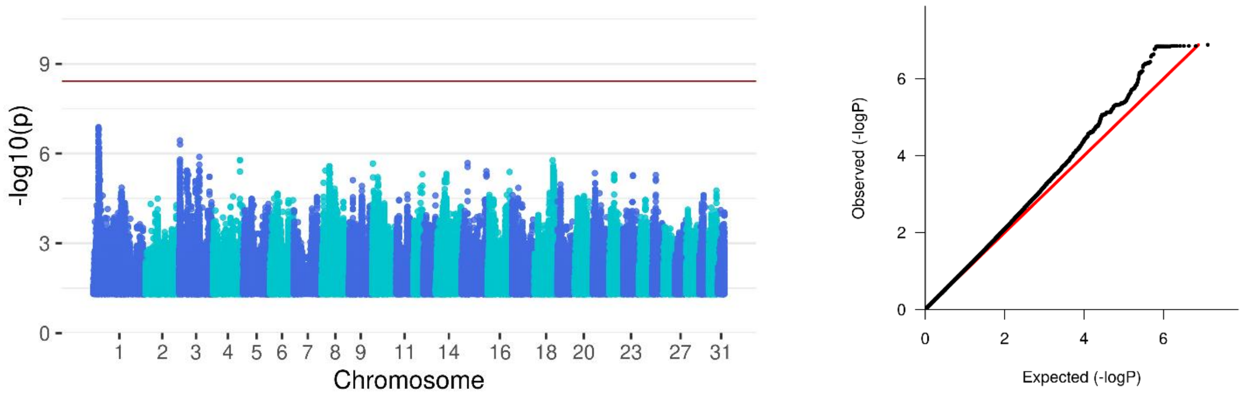

λ = 1.039

Length of croup [short - long]

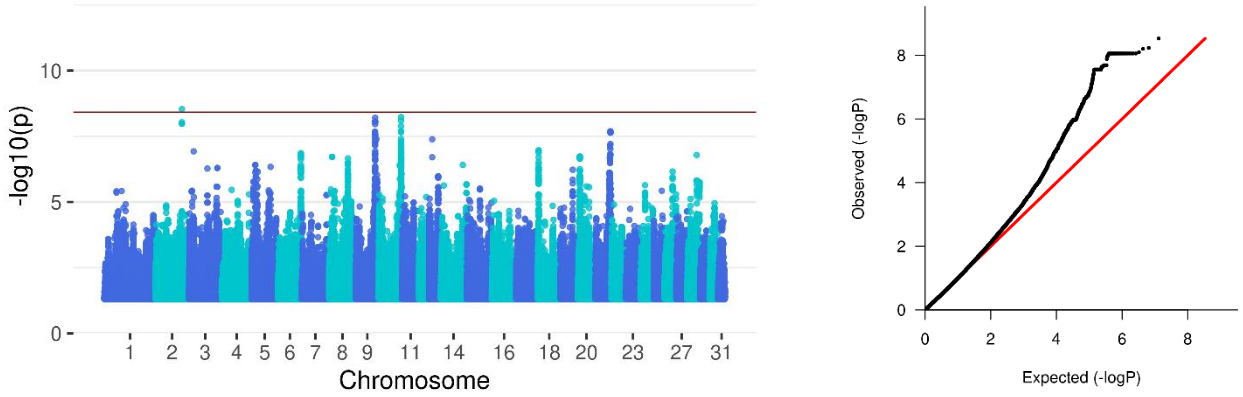

λ = 0.950

Angle (inclination) of croup [flat (level) - sloping]

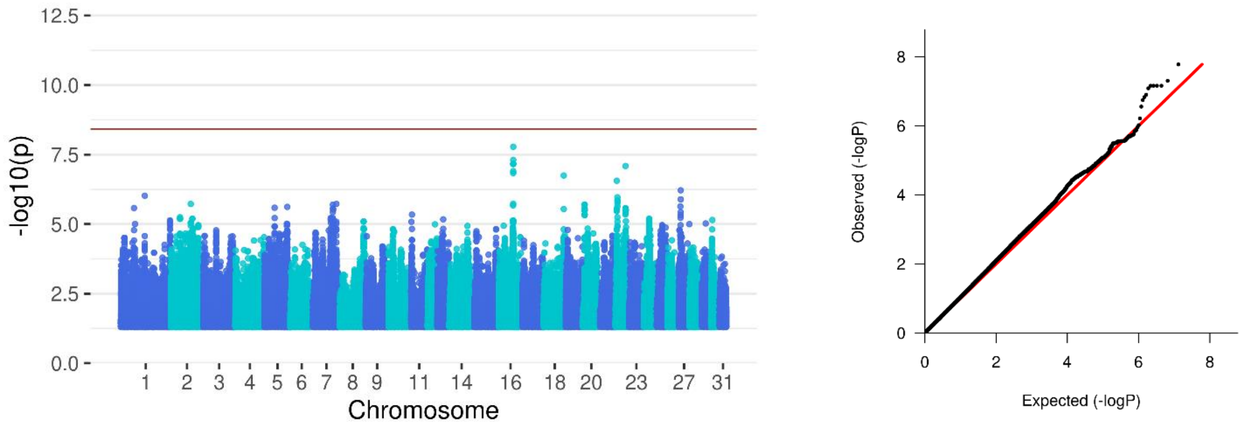

λ = 1.033

Shape of croup [angular - round]

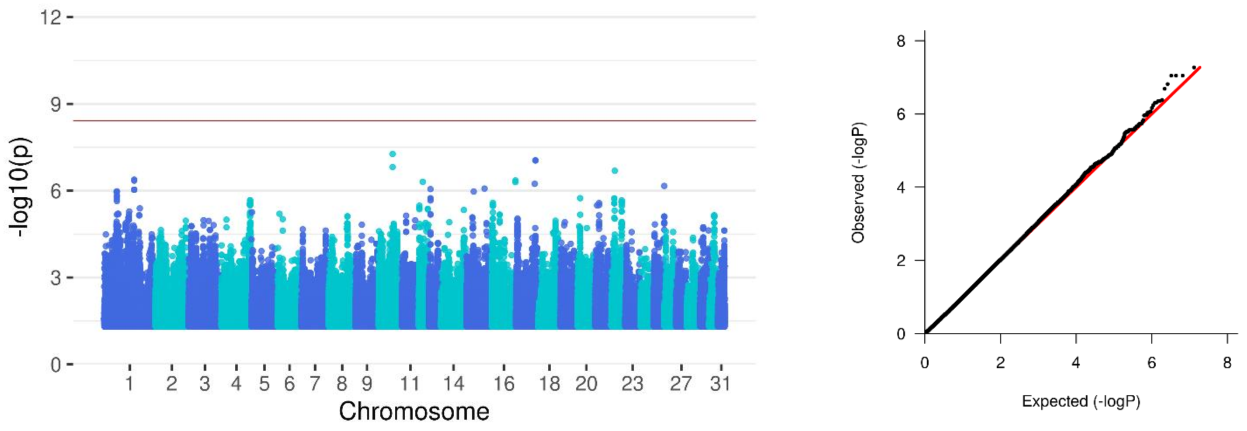

λ = 0.948

Set of tail [low - high]

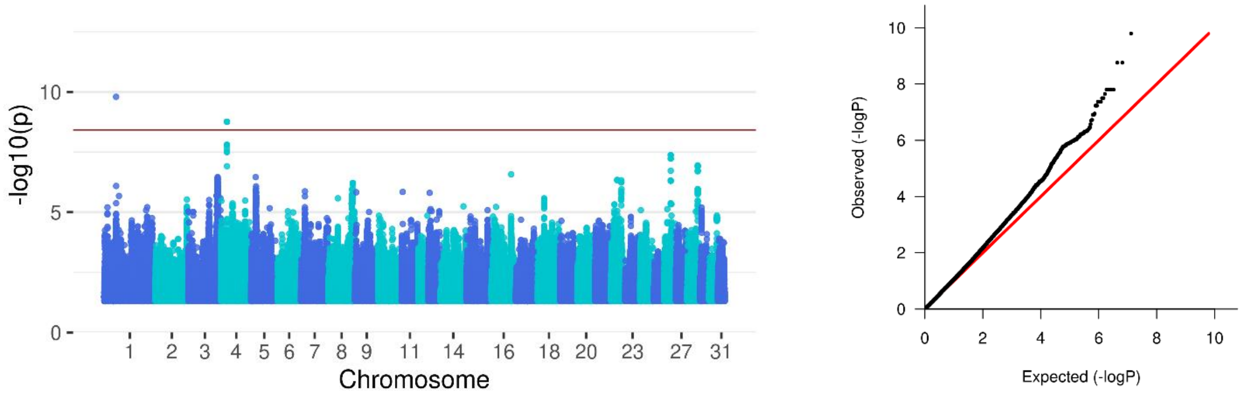

λ = 1.060

Position of carpus [over at knee - back at knee]

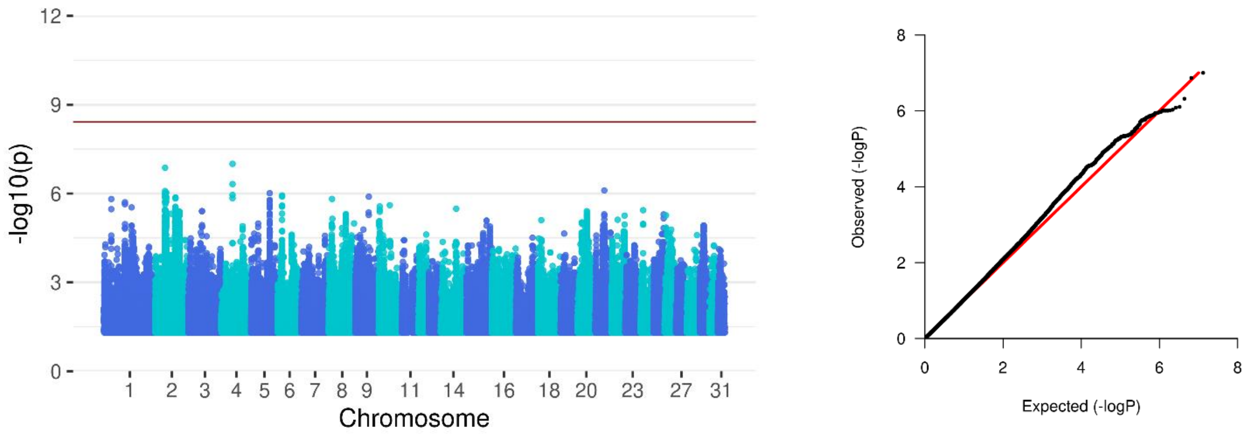

λ = 1.008

Length of forelimb pastern [short - long]

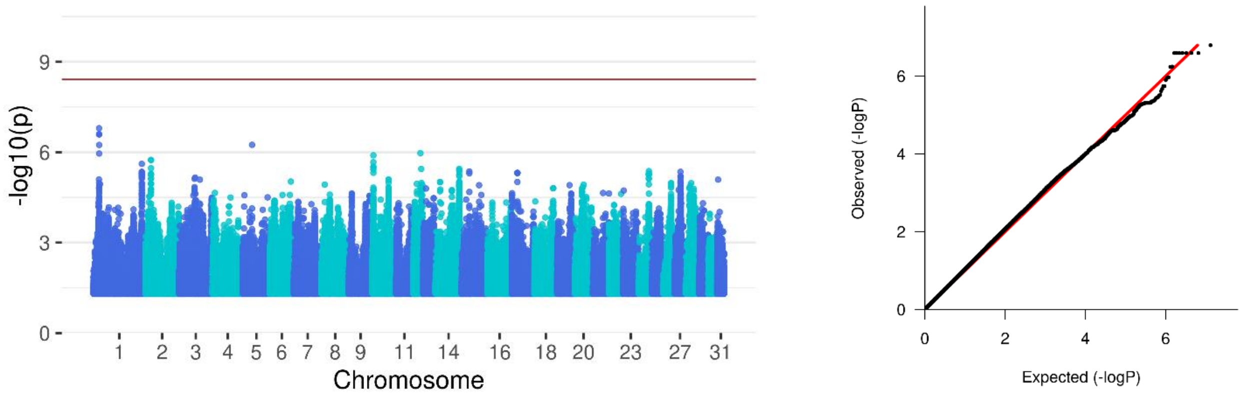

λ = 1.038

Stance of forelimb pastern [upright - sloping (weak)]

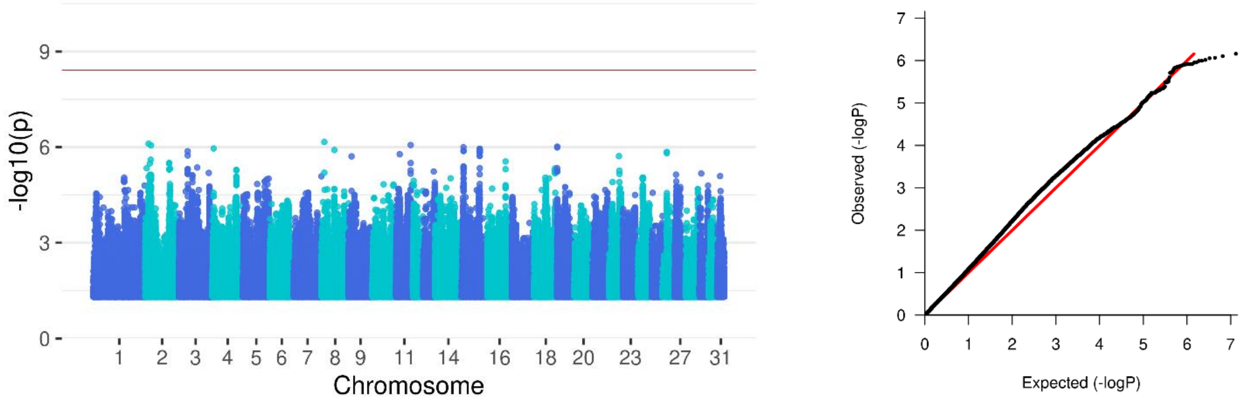

λ = 1.102

Broken toe axis in front limbs [markedly broken toe axis]

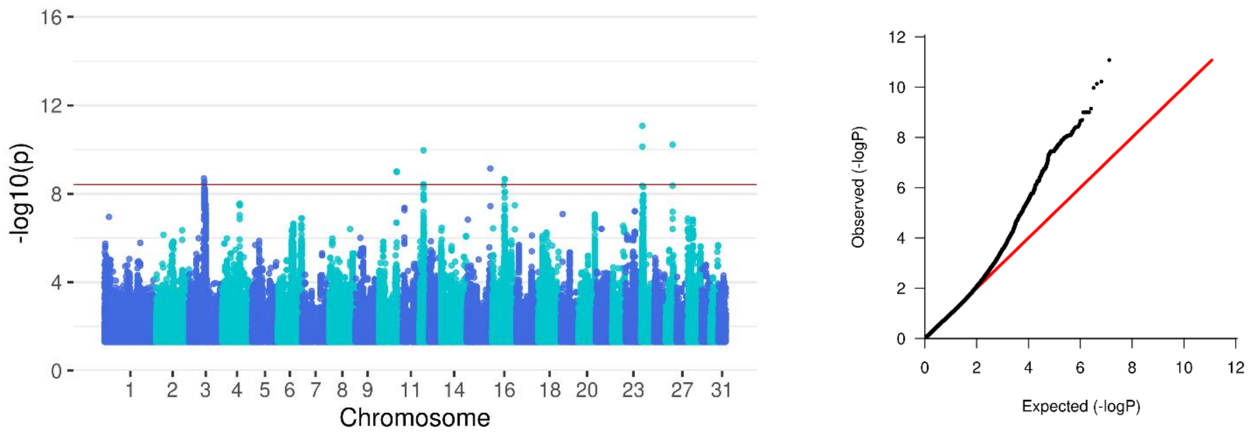

λ = 1.004

Definition of foreleg joints [flat (weak) - distinct]

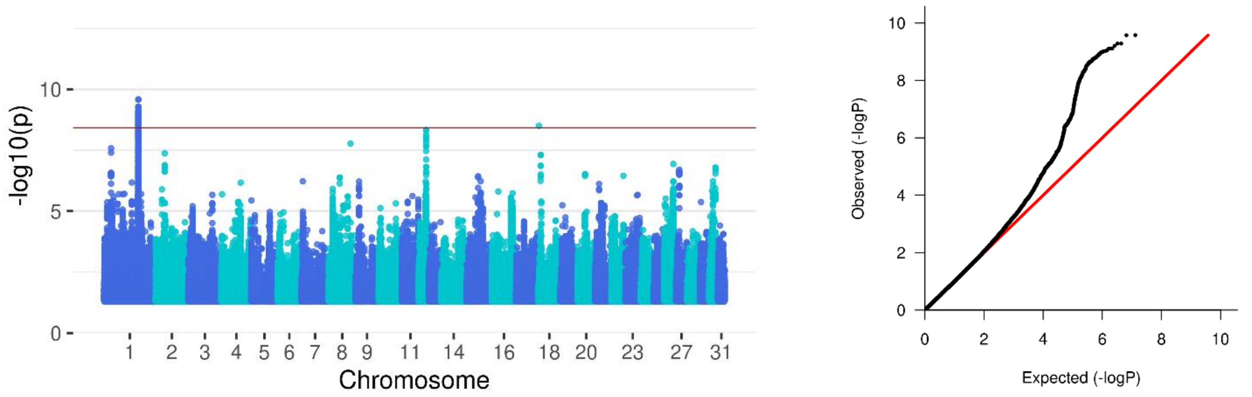

λ = 0.988

Definition of carpus-cannon articulation [flat - tied-in]

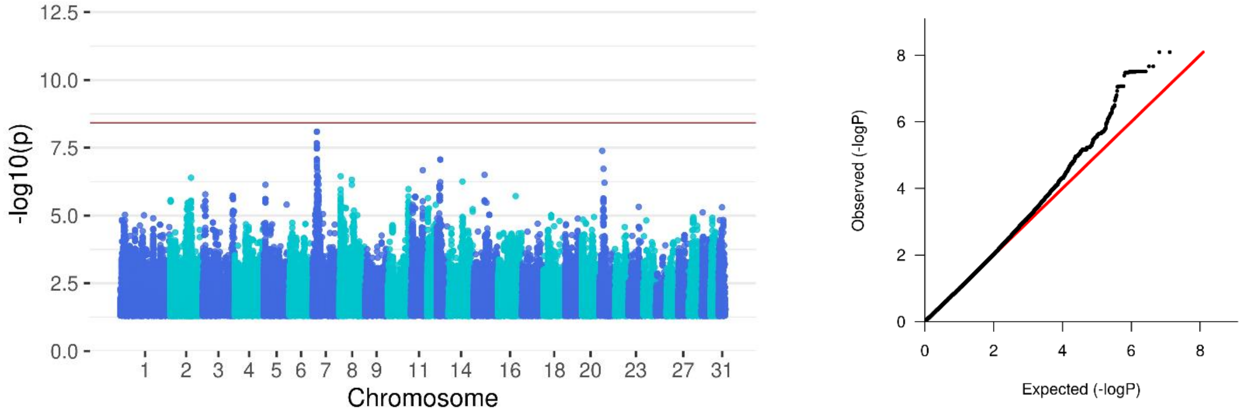

λ = 0.990

Length of hind limb pastern [short - long]

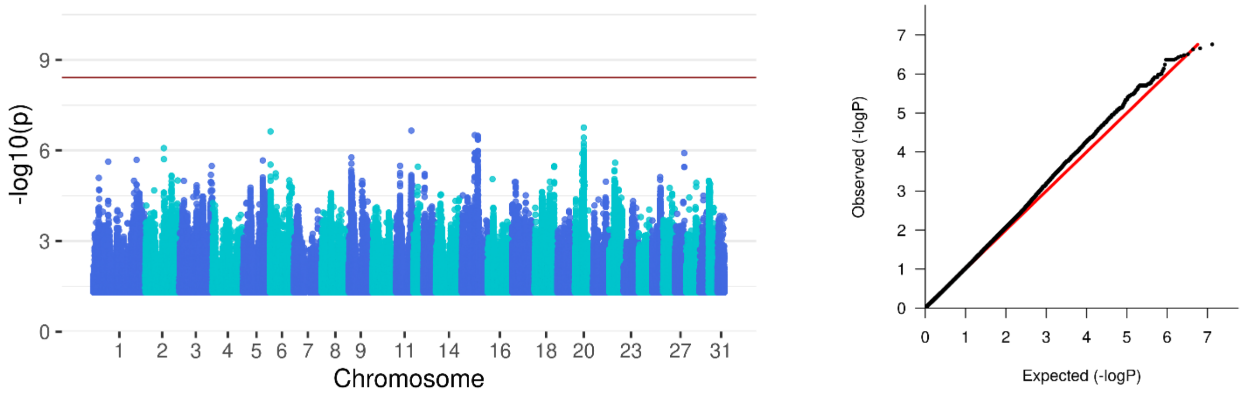

λ = 1.024

Stance of hind limb pastern [upright - weak]

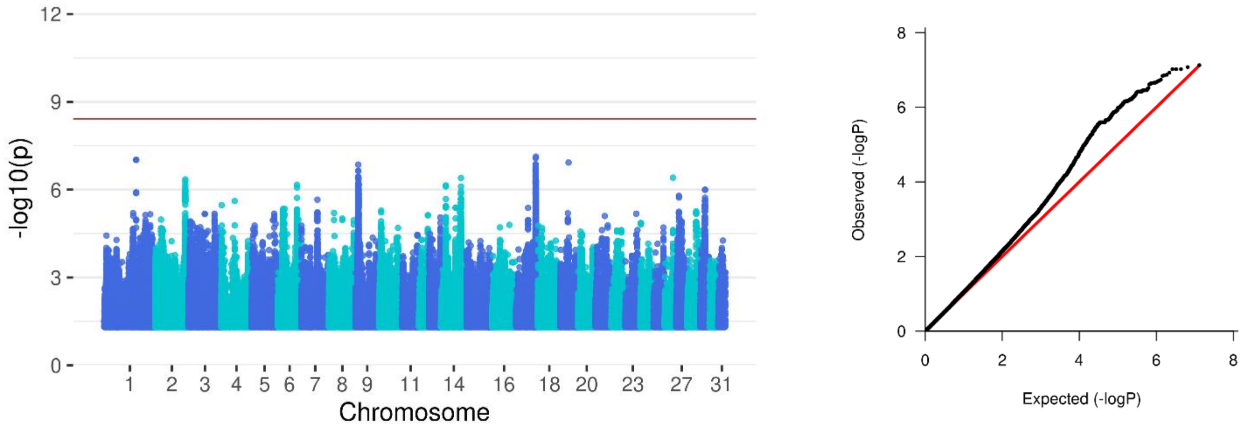

λ = 1.050

Hock angulation [straight - angulated]

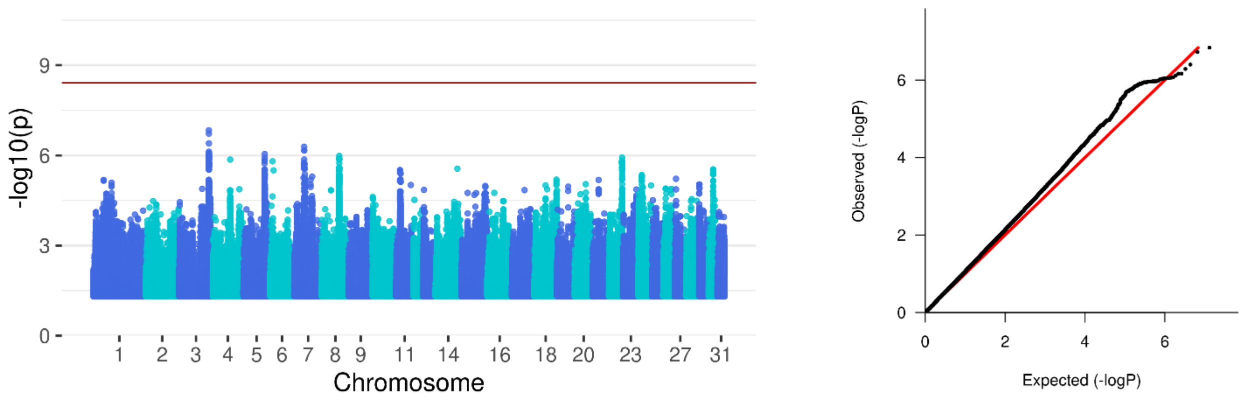

λ = 1.076

Hind leg [round]

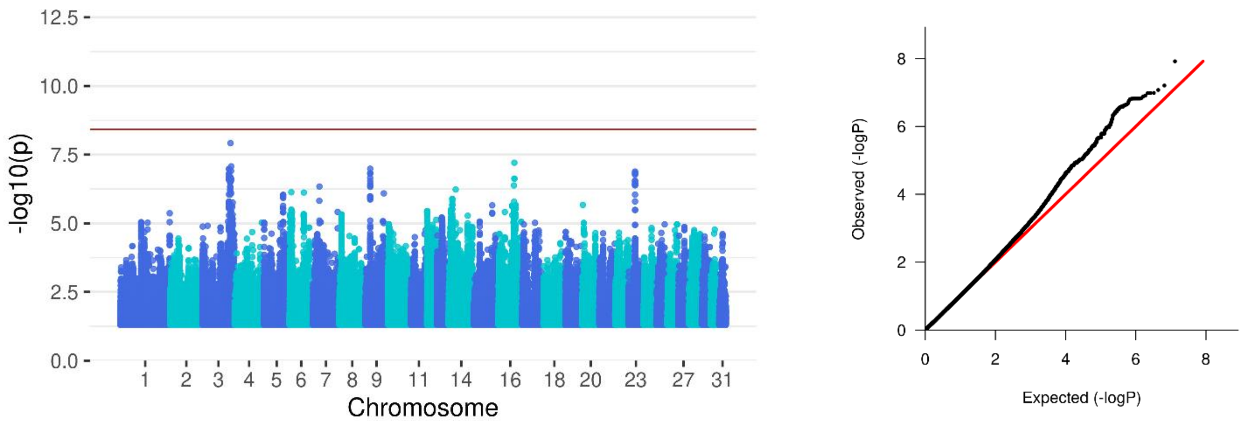

λ = 1.014

Capped hock [markedly capped hock]

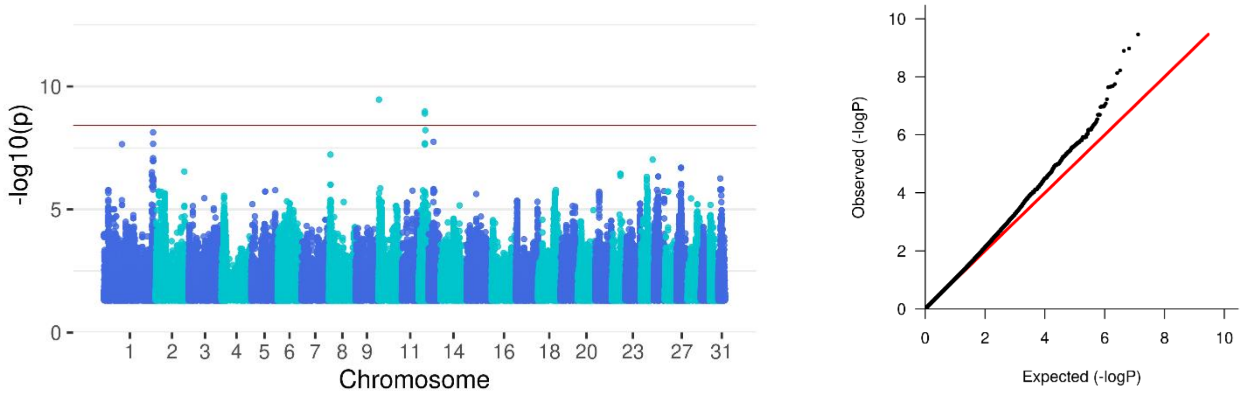

λ = 1.050

Definition of tarsus-cannon articulation [flat - tied-in]

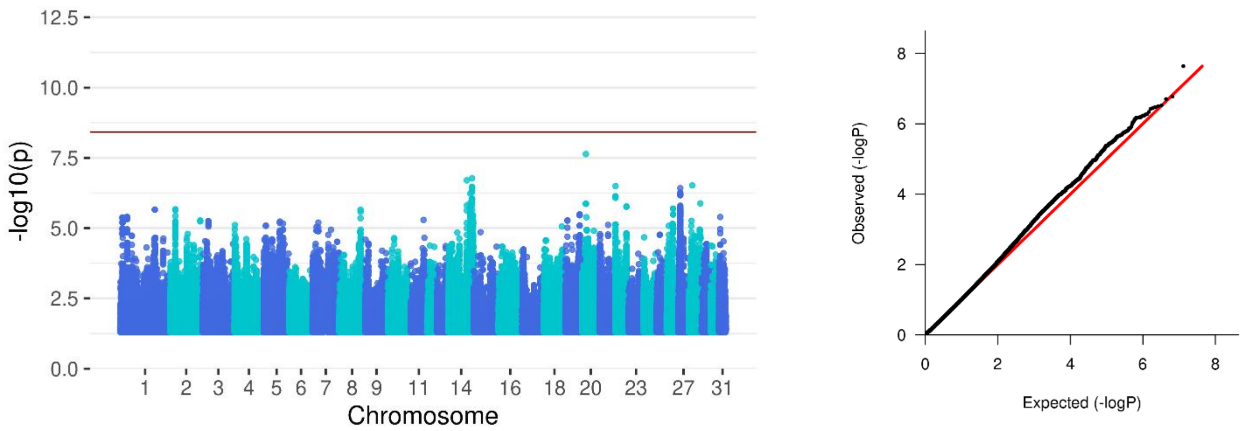

λ = 0.985

Size of joints [small - big]

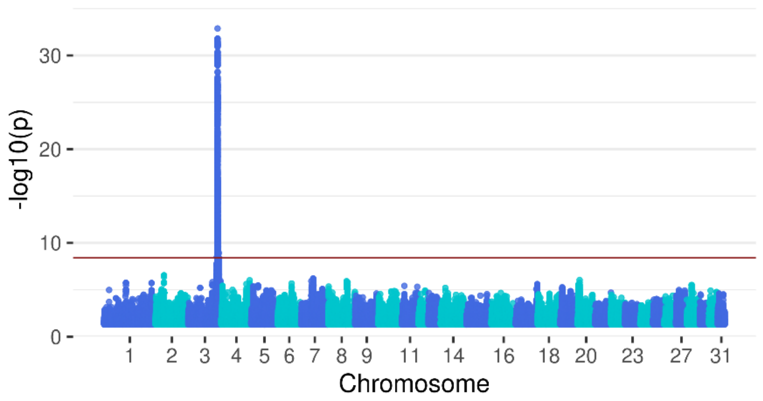

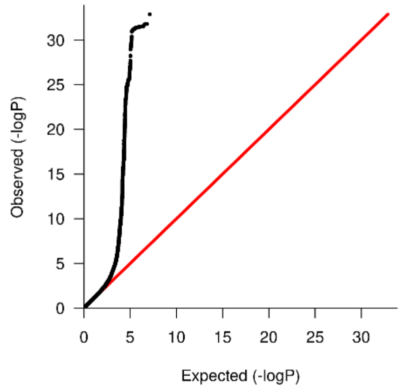

λ = 1.024

Shape of feet (hoof size) [narrow, small - wide, big]

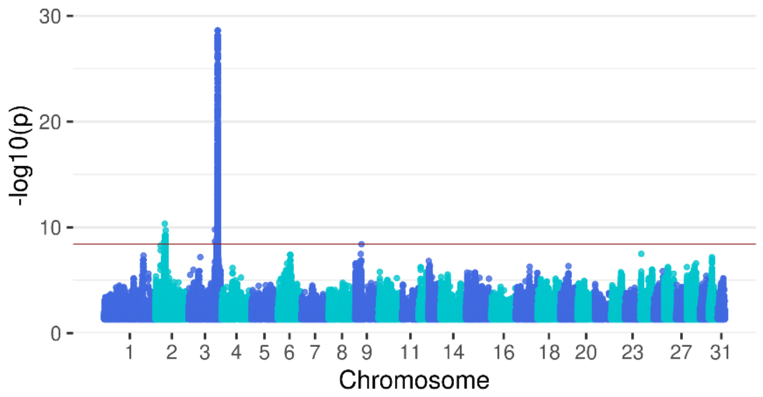

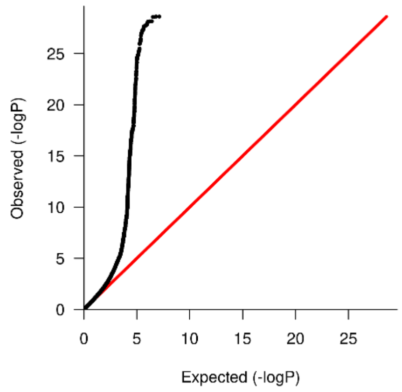

λ = 1.066

Heel height [flat hoof (-3), low heels - high heels, club foot (+3)]

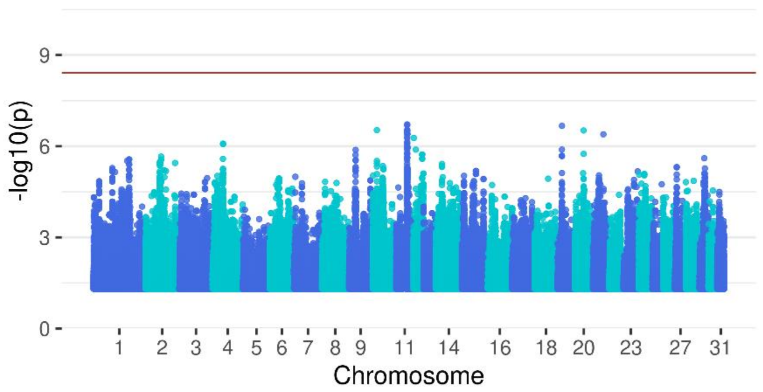

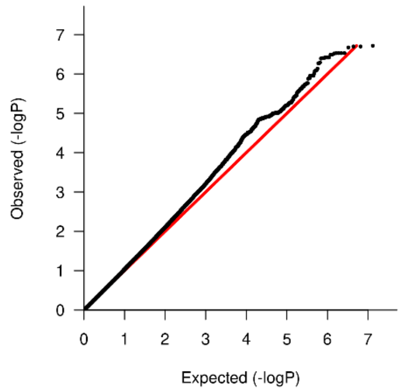

λ = 1.045

Hoof asymmetry (uneven shape of feet) [markedly uneven feet]

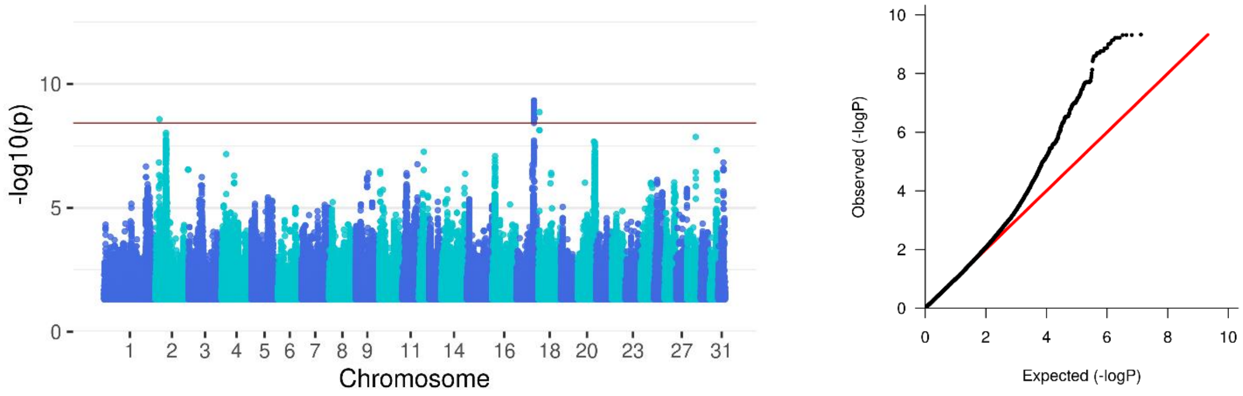

λ = 0.990

Toe stance of forelegs [toe-in - toe-out]

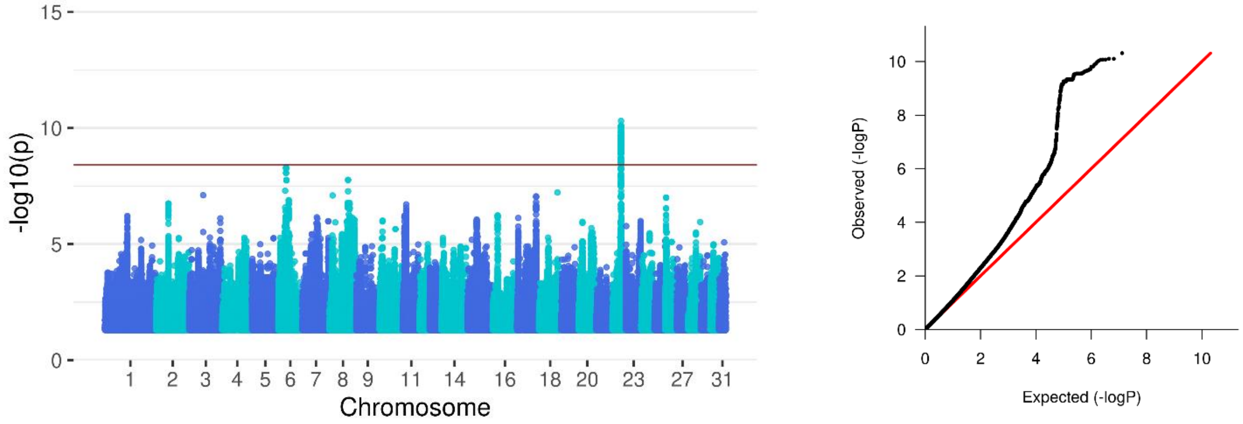

λ = 1.087

Standing position of front limbs [base-narrow - base-wide]

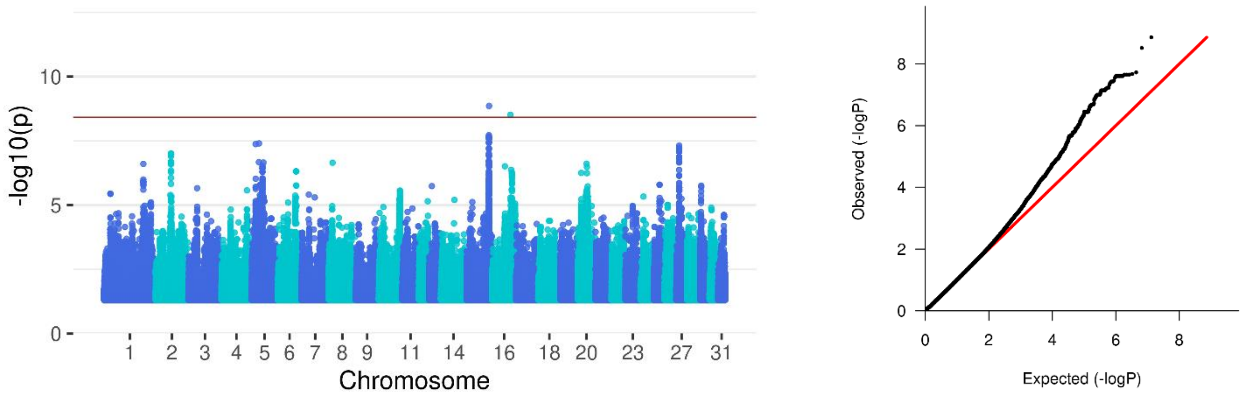

λ = 0.981

Toe stance of hind legs [toe-in - toe-out]

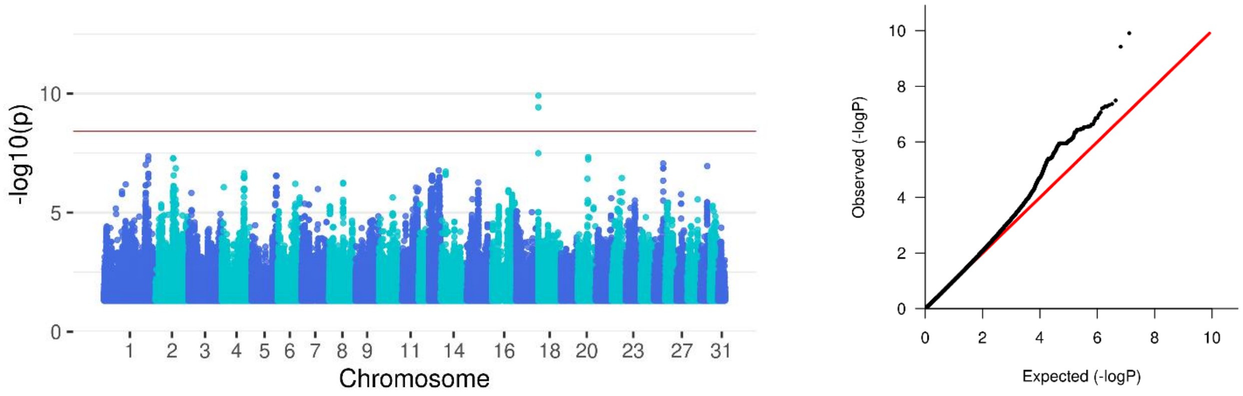

λ = 0.976

Standing position of hind limbs [base-narrow - base-wide]

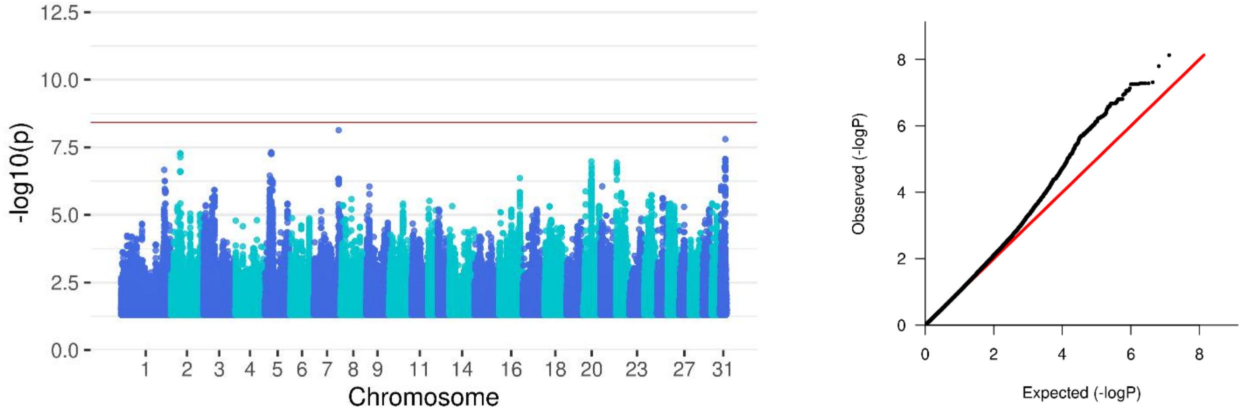

λ = 0.986

Position of hock - back view [bow-hocked - cow-hocked]

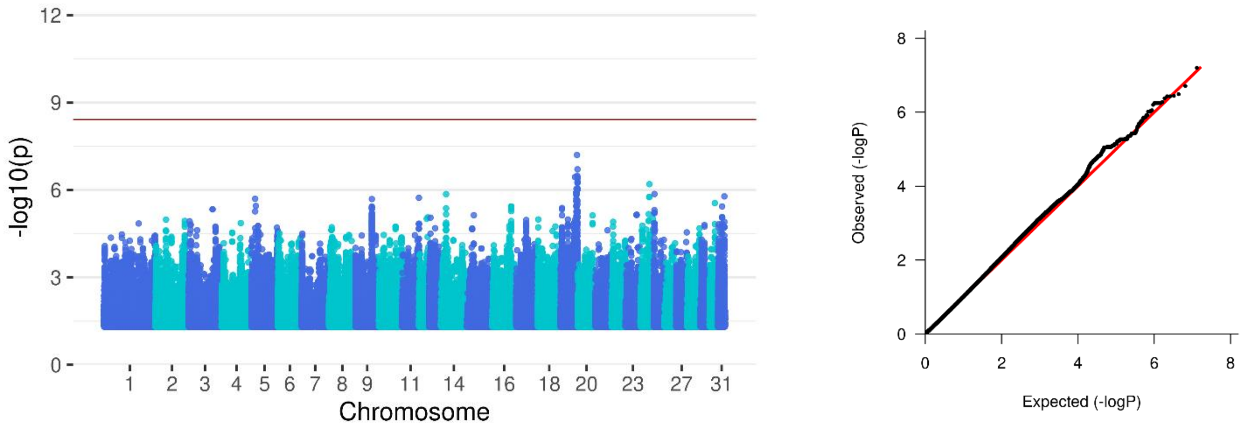

λ = 0.979

Correctness of limb movement [plaiting (brushing) - dishing (winging)]

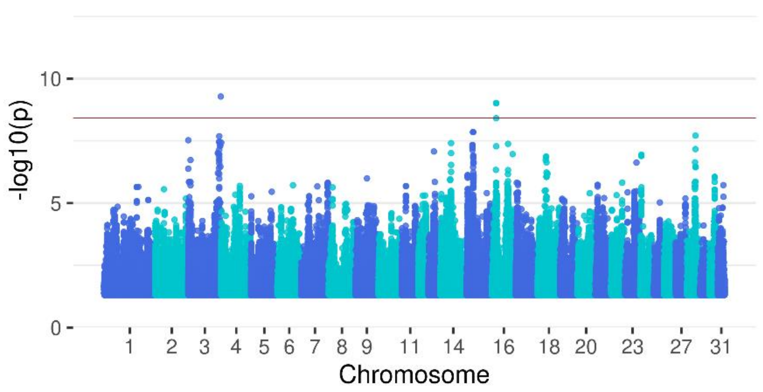

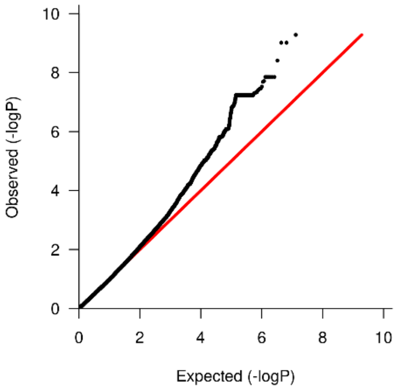

λ = 0.958

Rotation in the hock [marked rotation]

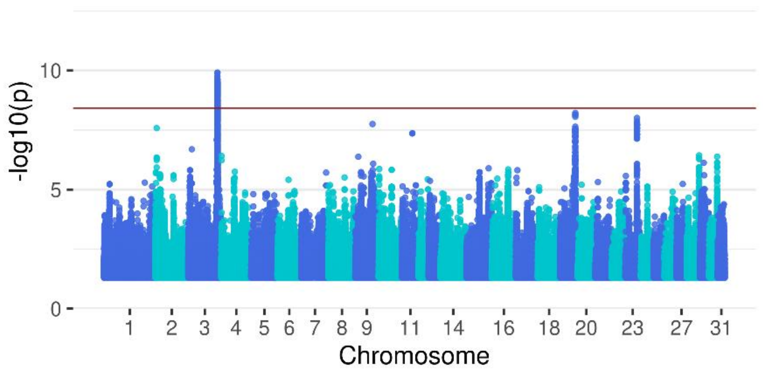

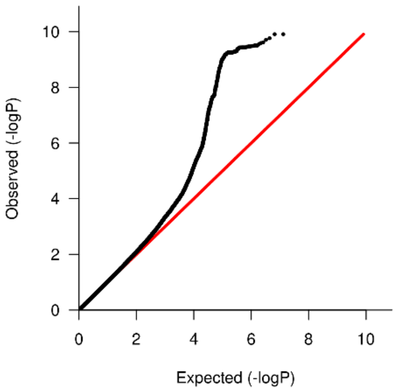

λ = 1.017

Tail position [markedly off-center]

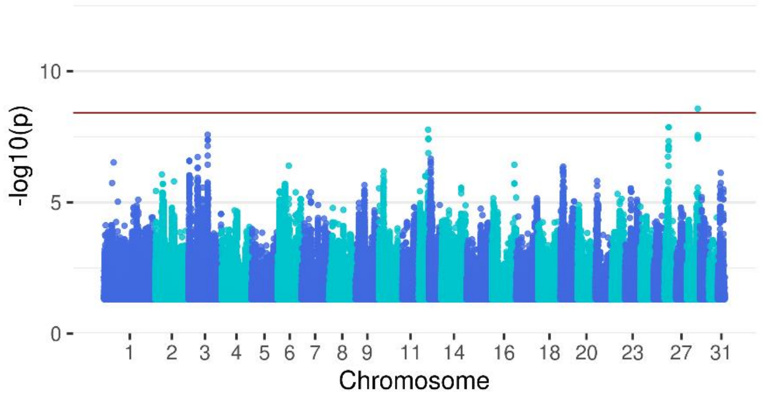

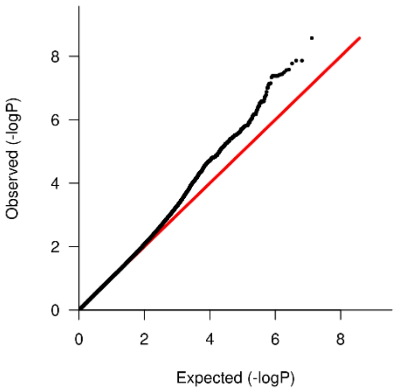

λ = 1.046

Tail tone [un-toned - over-toned]

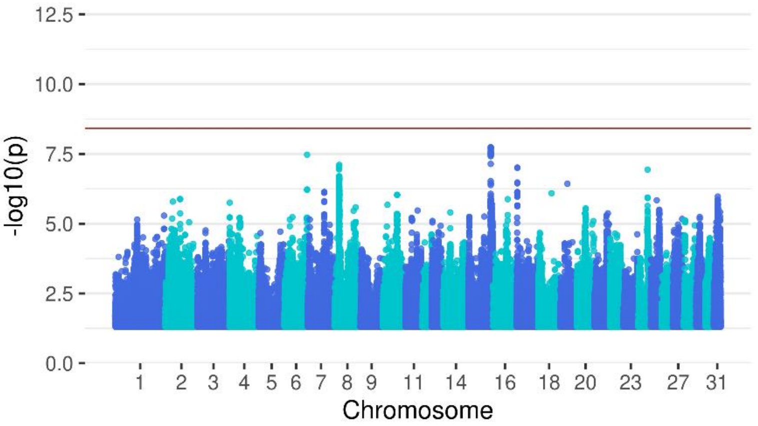

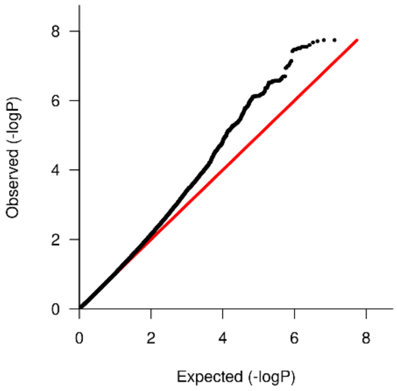

λ = 1.008
