# Supplementary material for: Genomic analyses of withers height and linear conformation traits in German Warmblood horses using imputed sequence-level genotypes
Source: Genet Sel Evol. 2024 Jun 13;56:45. doi: 10.1186/s12711-024-00914-6 (PMC11177368; doi:10.1186/s12711-024-00914-6)
Supplement: Supplementary file 8 — Additional file 8: Figure S4. Results of the conditional genome-wide association studies in 4769 to 4891 horses (depending on the trait) for 13 conformation traits showing a peak on chromosome 3 next to the LCORL/NCAPG-locus. Manhattan plots of the –log10 p-values for the association of variants with withers height. The dark red horizontal line indicates the genome-wide significance threshold with α = 0.05 and Bonferroni correction for multiple testing (p = 3.8 × 10^–9^). Due to computational limitations, variants with a p-value > 0.05 were excluded from the plots. Either the top associated SNP from the GWAS for the respective conformation trait itself, the top associated SNP from the GWAS for withers height, or withers height itself was included in the initial model as an additional fixed effect. In addition to the Manhattan plots (left-hand side), the respective quantile–quantile plots for the traits are given (right-hand side). The observed p-values (black) are plotted against the expected p-values (red) and have a genomic inflation factor of λ (stated below the plot). [file 12711_2024_914_MOESM8_ESM.pdf]

<u>Breed type</u> [plain - true to type]

Fixed effect: withers height

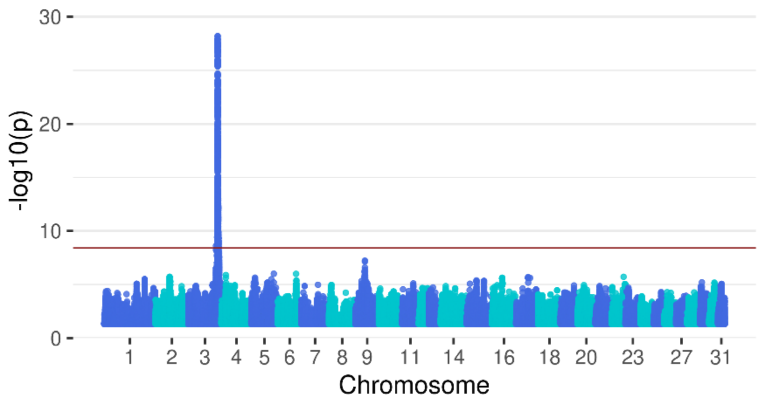

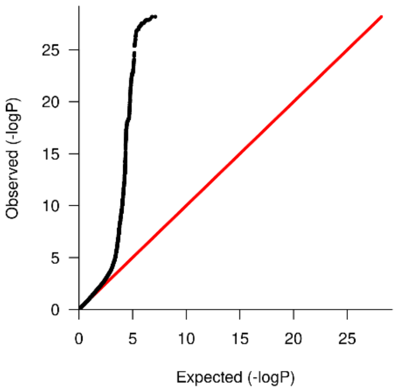

λ = 1.085

Fixed effect: top SNP from GWAS withers height (rs68603062) = own top SNP from GWAS

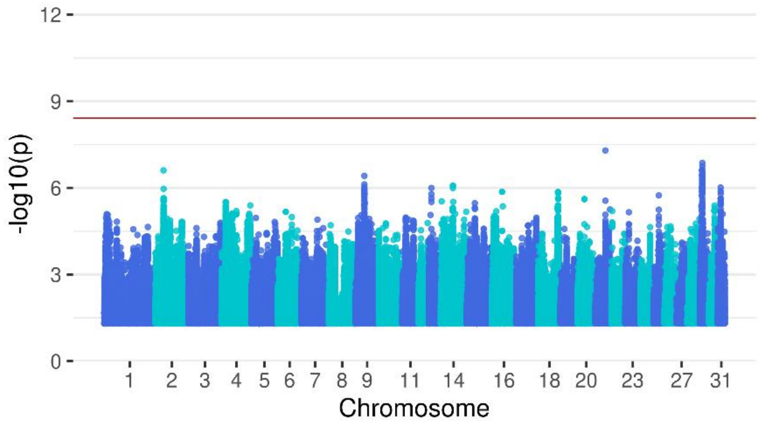

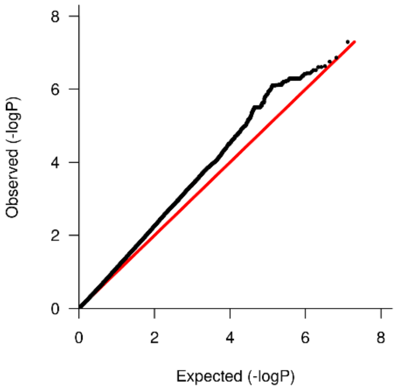

λ = 1.127

<u>Gender expression</u> <u>[weak - strong]</u>

Fixed effect: withers height

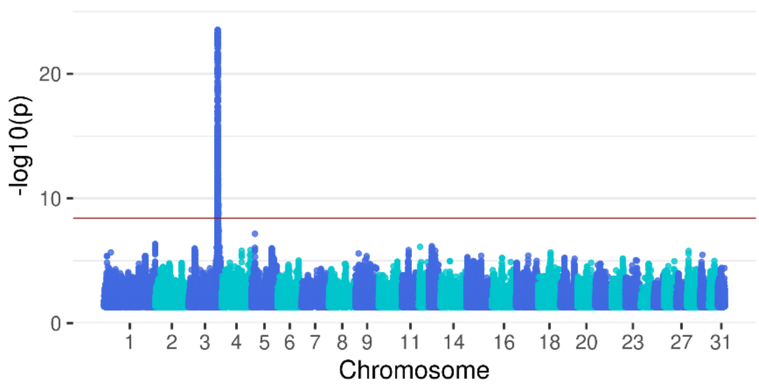

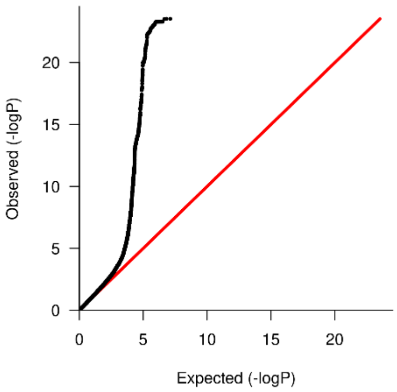

λ = 1.077

Fixed effect: top SNP from GWAS withers height (rs68603062)

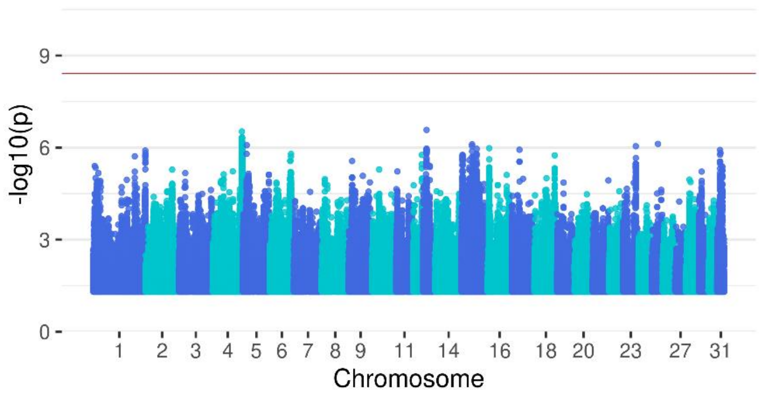

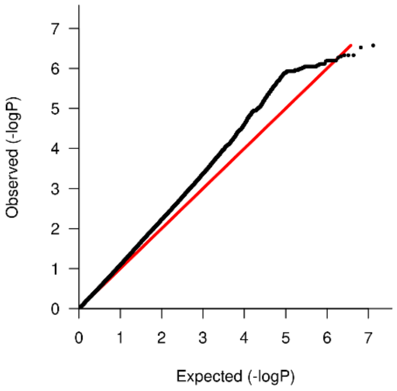

λ = 1.111

Fixed effect: own top SNP from GWAS (rs1139838416)

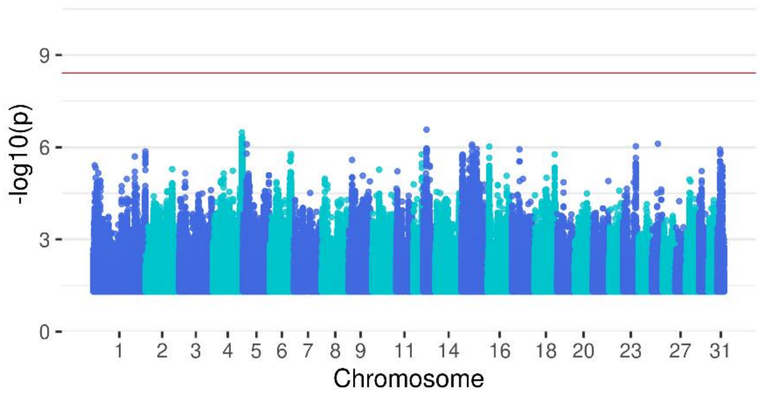

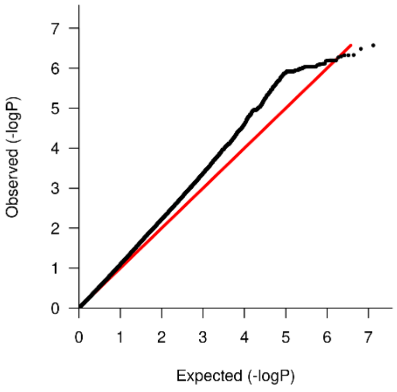

λ = 1.111

Frame [small-framed - large-framed]

Fixed effect: withers height

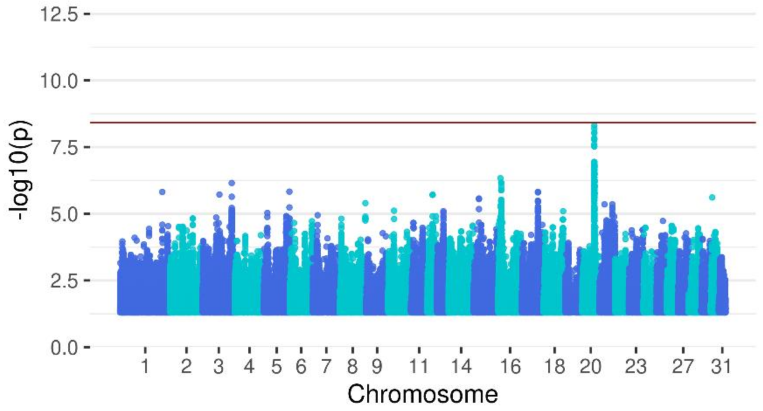

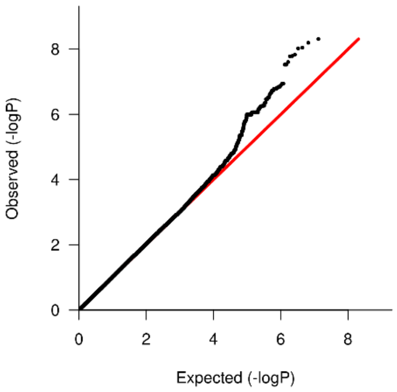

λ = 1.010

Fixed effect: top SNP from GWAS withers height (rs68603062)

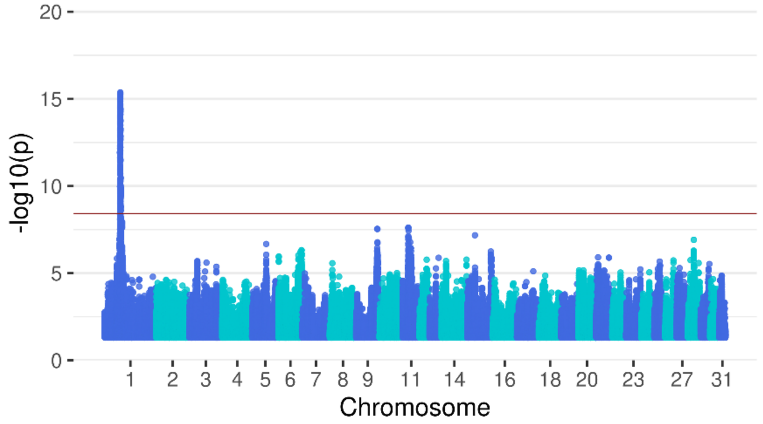

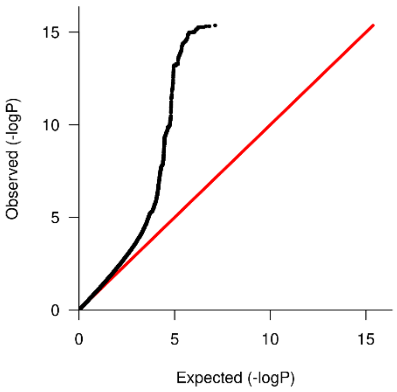

λ = 1.121

Fixed effect: own top SNP from GWAS (rs1145719639)

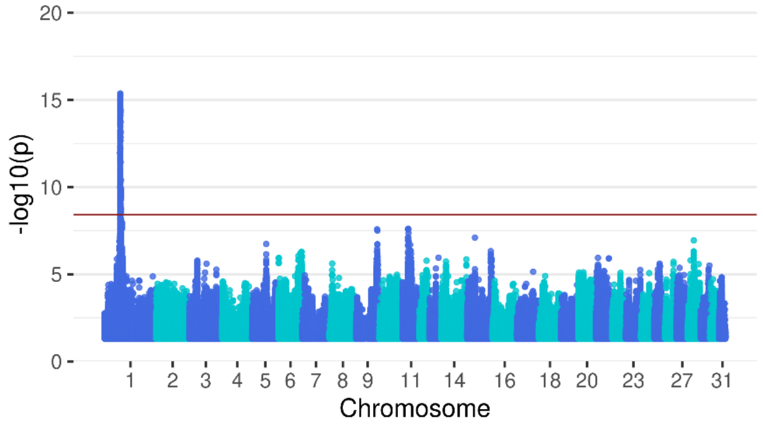

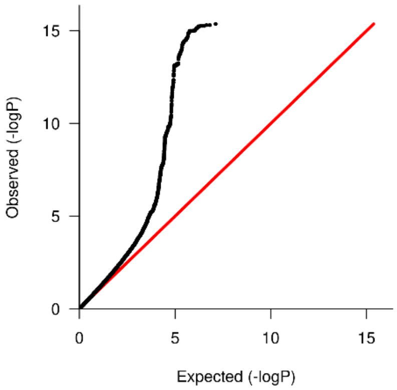

λ = 1.122

<u>Caliber</u> <u>[light - heavy]</u>

Fixed effect: withers height

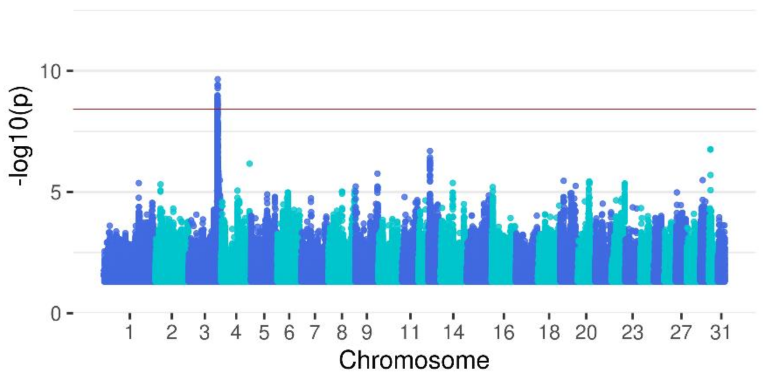

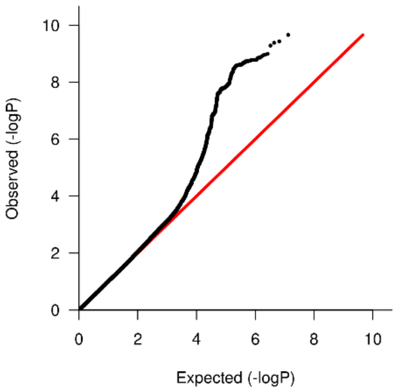

λ = 0.998

Fixed effect: top SNP from GWAS withers height (rs68603062)

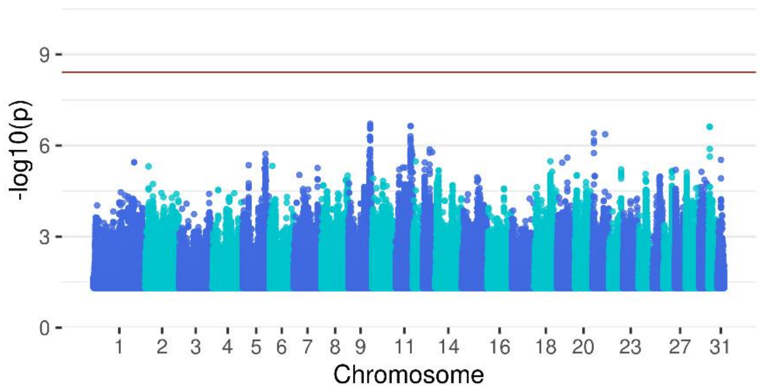

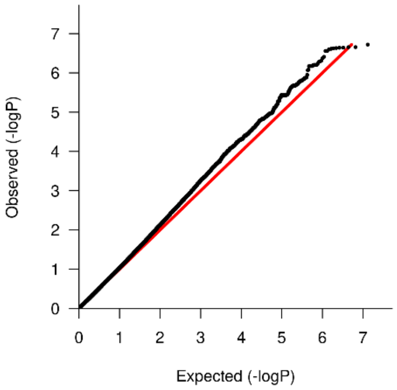

λ = 1.029

Fixed effect: own top SNP from GWAS (rs1141597173)

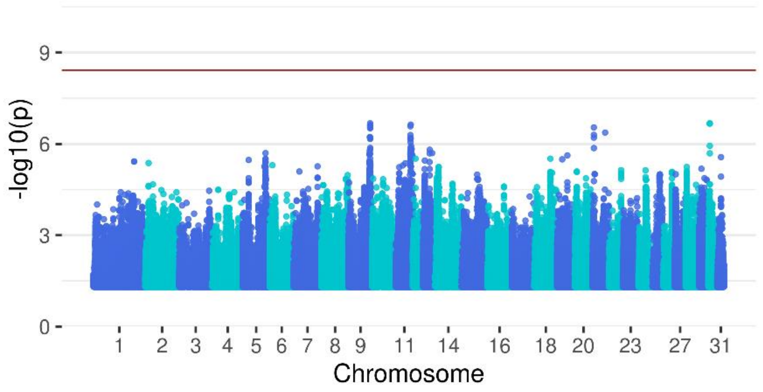

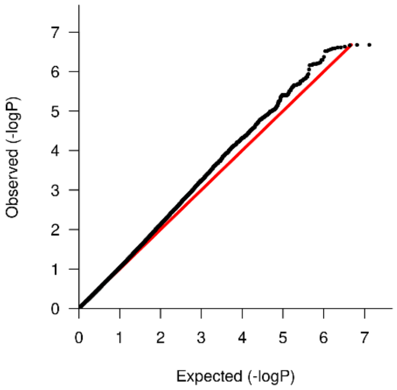

λ = 1.029

Length of legs [short-legged - long-legged]

Fixed effect: withers height

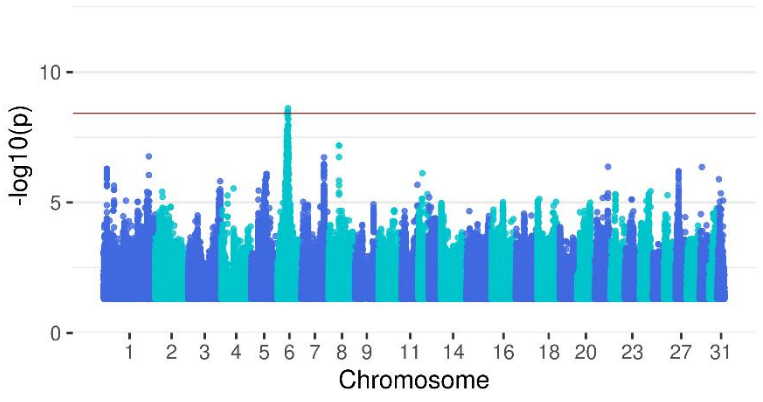

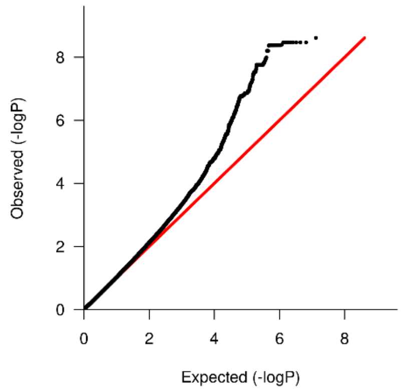

λ = 1.006

Fixed effect: top SNP from GWAS withers height (rs68603062)

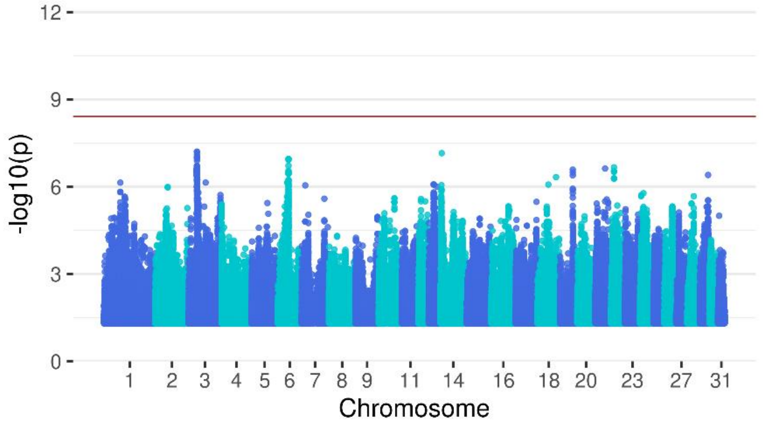

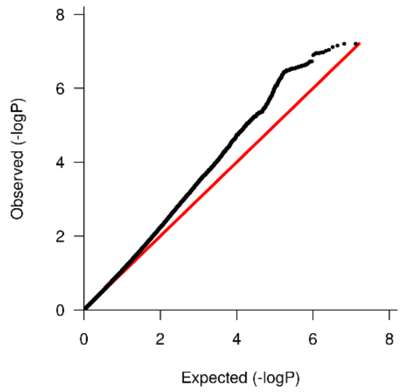

λ = 1.071

Fixed effect: own top SNP from GWAS (rs1139838416)

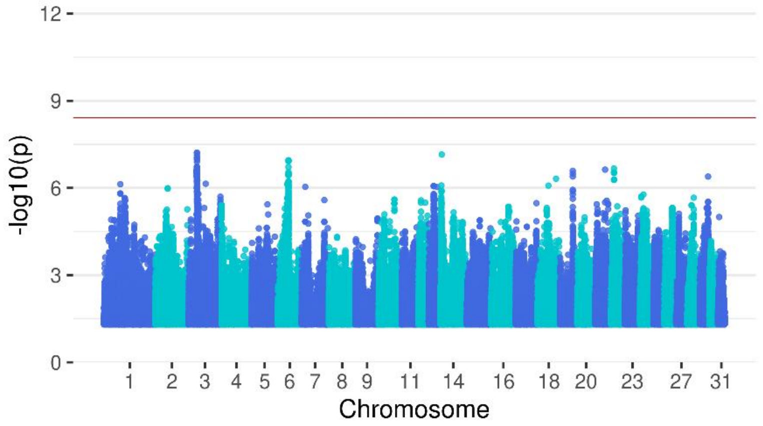

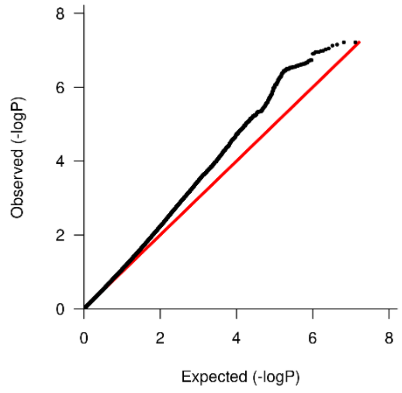

λ = 1.071

<u>Body shape</u> <u>[square - (long-)rectangular]</u>

Fixed effect: withers height

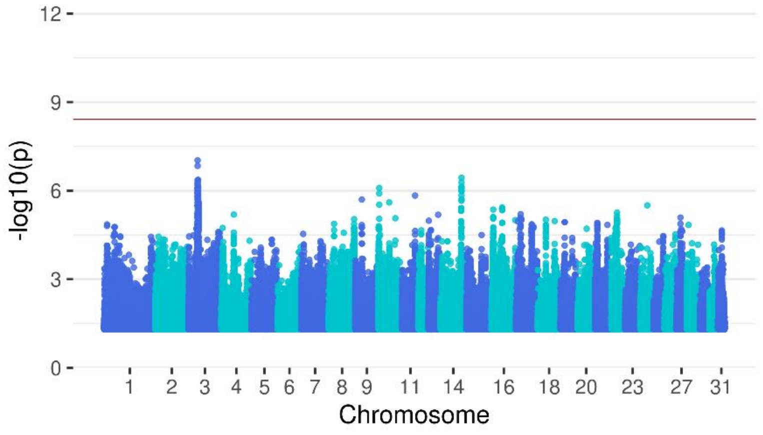

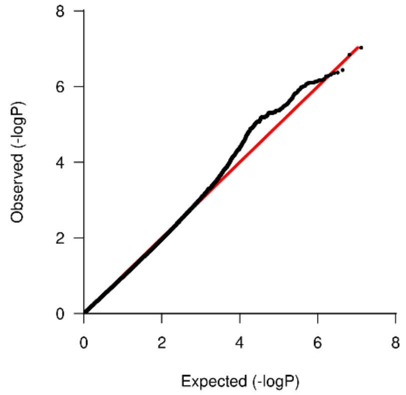

λ = 0.953

Fixed effect: top SNP from GWAS withers height (rs68603062)

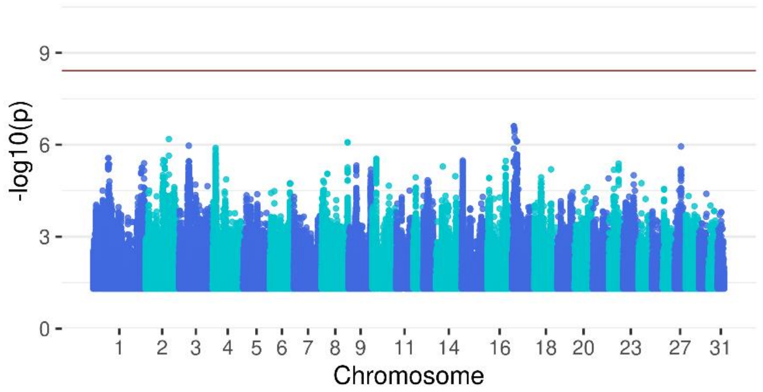

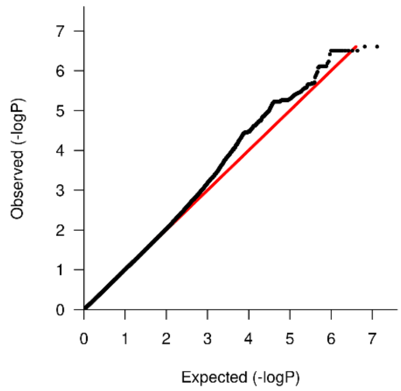

λ = 0.998

Fixed effect: own top SNP from GWAS (rs1138898791)

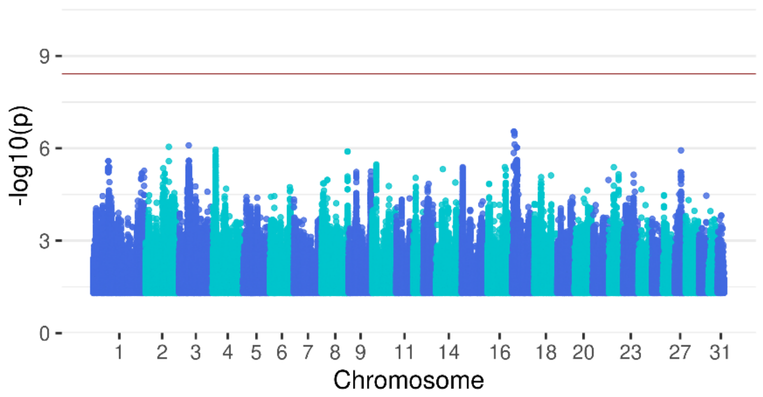

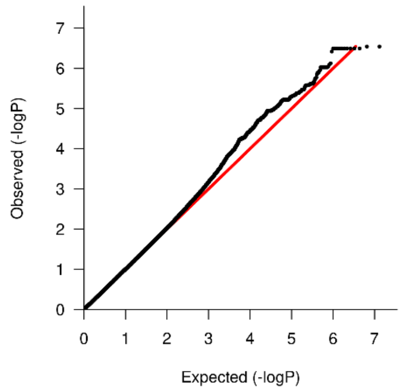

λ = 0.992

Head shape [coarse - fine]

Fixed effect: withers height

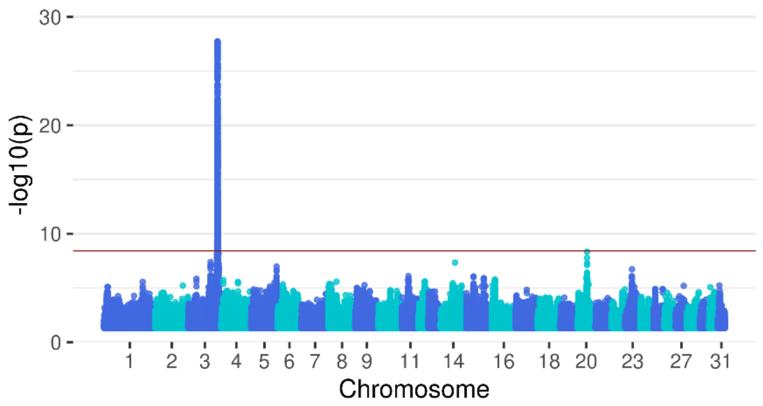

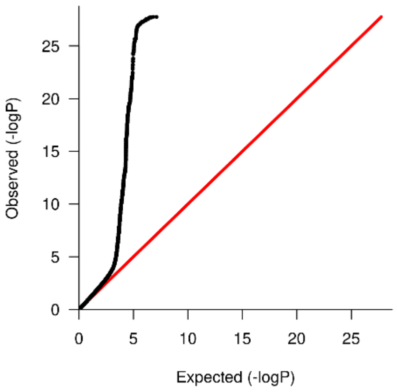

λ = 1.119

Fixed effect: top SNP from GWAS withers height (rs68603062) = own top SNP from GWAS

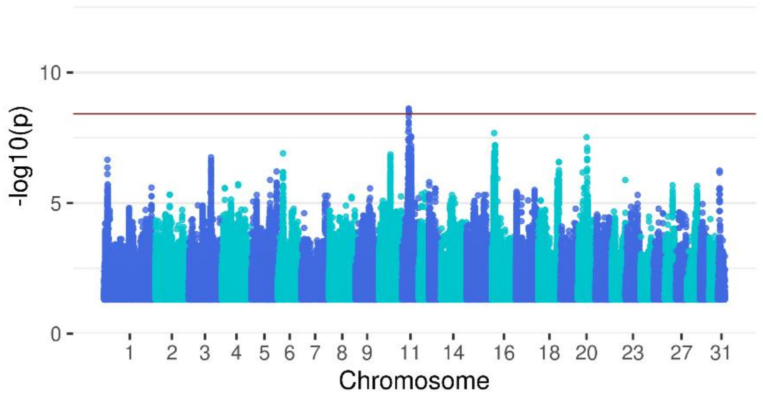

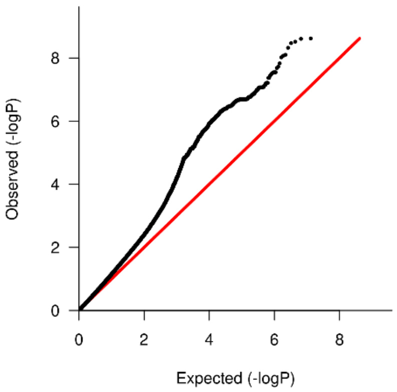

λ = 1.187

<u>Head length</u> [short - long]

Fixed effect: withers height

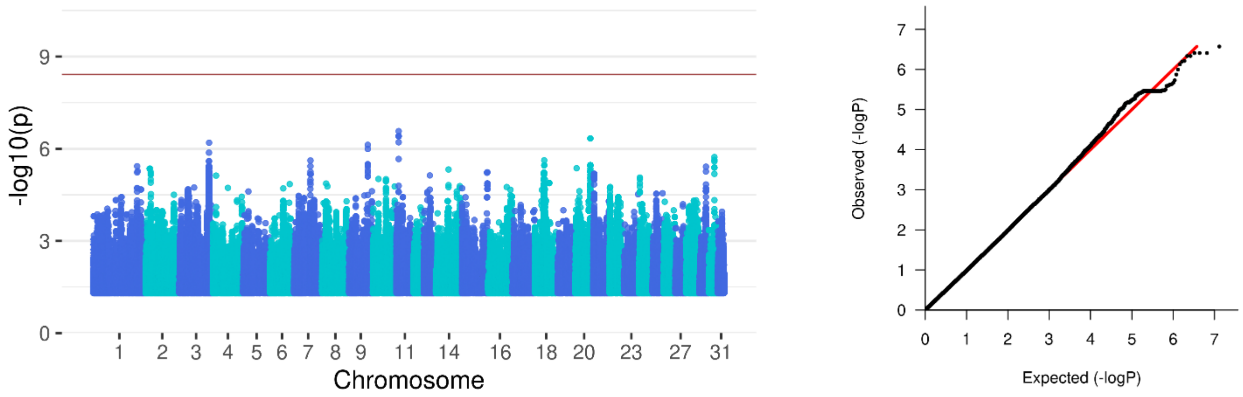

λ = 0.976

Fixed effect: top SNP from GWAS withers height (rs68603062)

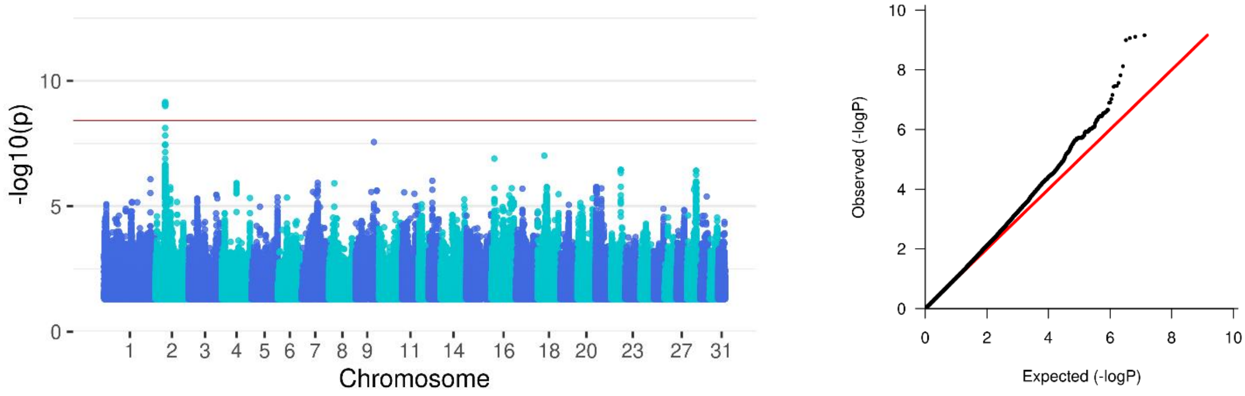

λ = 1.037

Fixed effect: own top SNP from GWAS (rs1151617437)

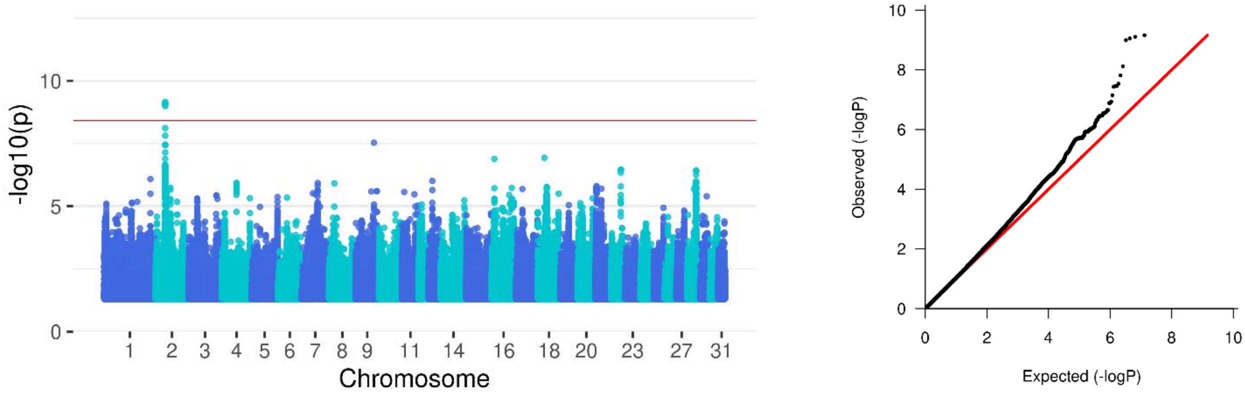

λ = 1.037

<u>**Eye size** [small - large]</u>

Fixed effect: withers height

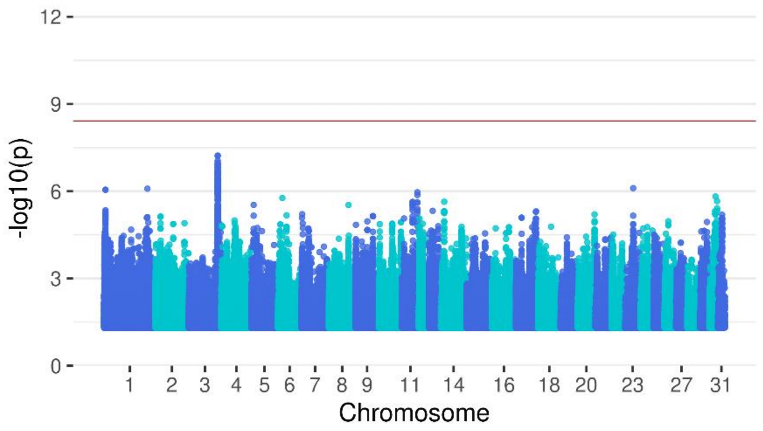

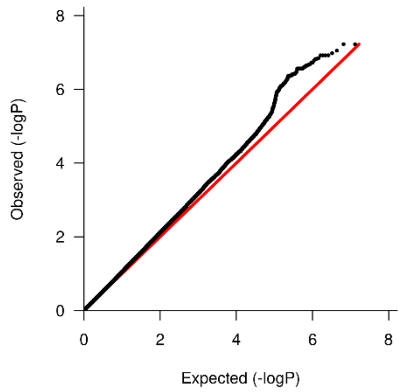

λ = 1.056

Fixed effect: top SNP from GWAS withers height (rs68603062)

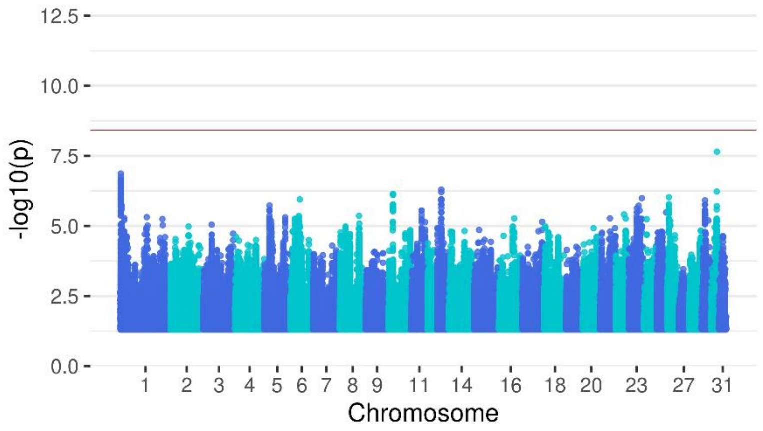

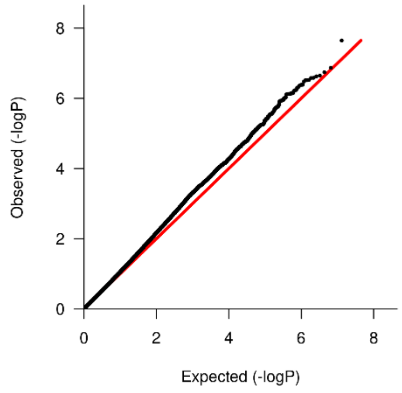

λ = 1.056

Fixed effect: own top SNP from GWAS (rs1141505835)

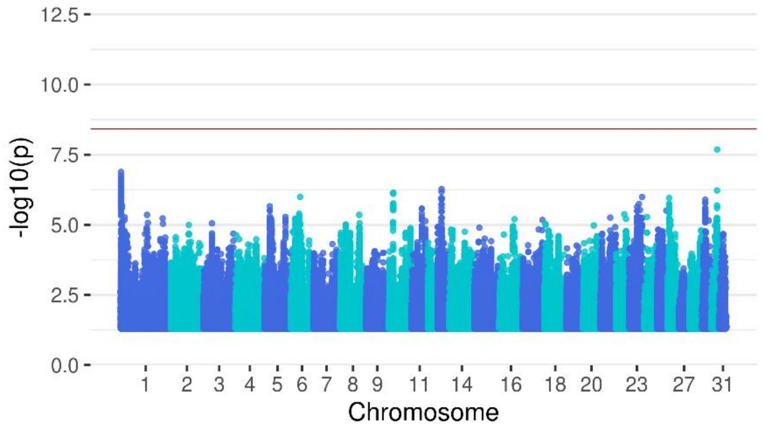

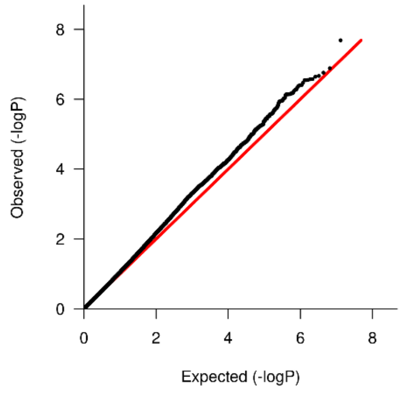

λ = 1.055

<u>Length of withers</u> <u>[short - long]</u>

Fixed effect: withers height

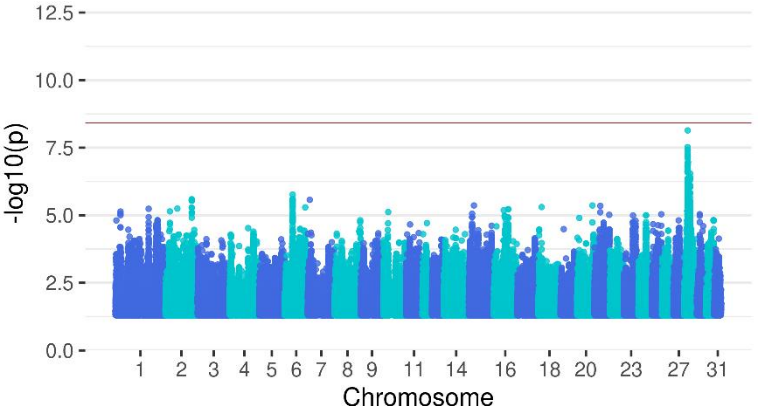

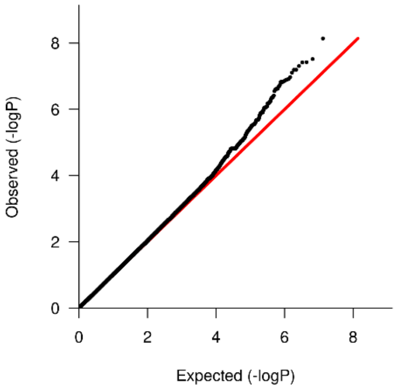

λ = 1.024

Fixed effect: top SNP from GWAS withers height (rs68603062)

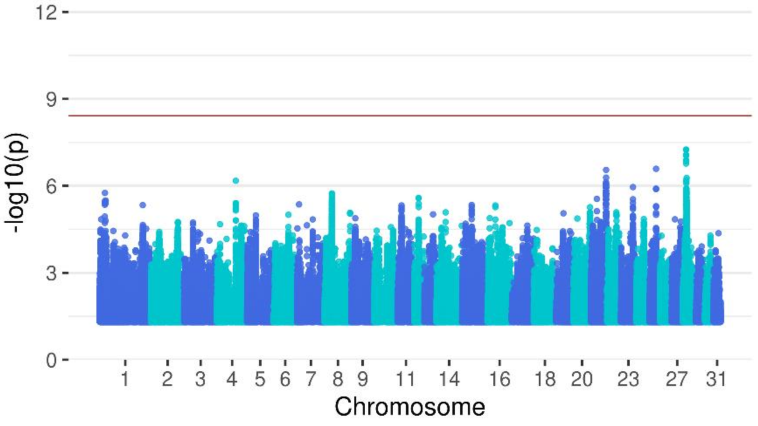

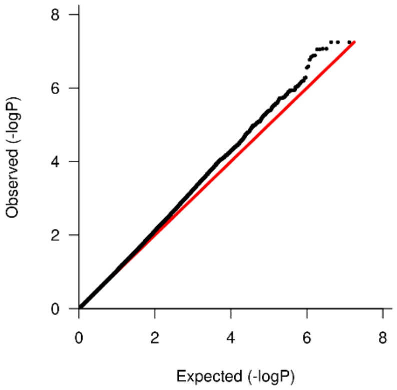

λ = 1.041

Fixed effect: own top SNP from GWAS (rs1152276079)

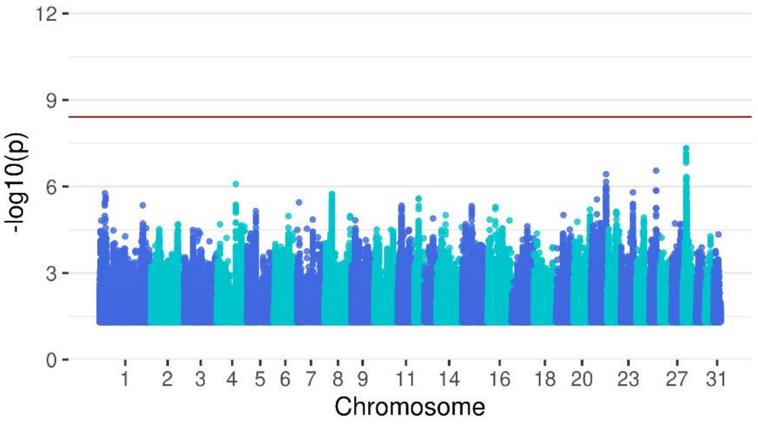

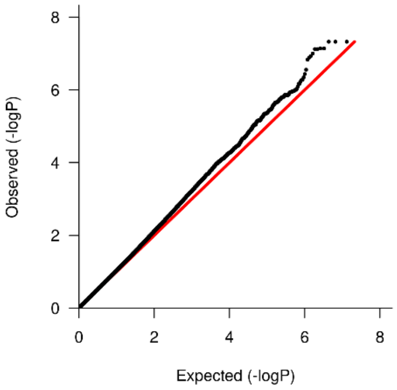

λ = 1.040

Size of joints [small - big]

Fixed effect: withers height

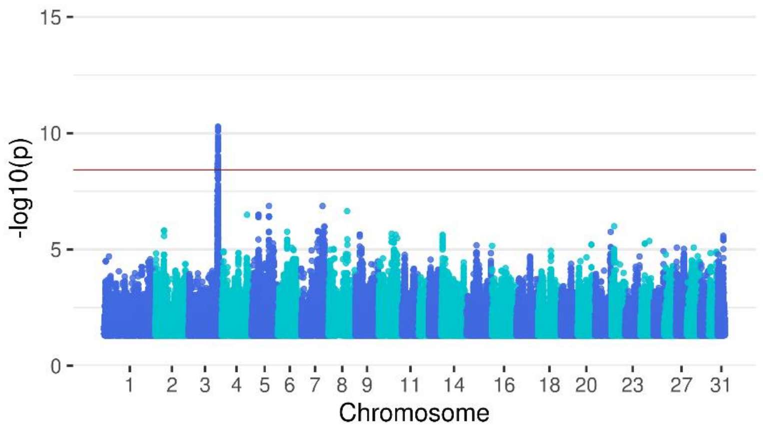

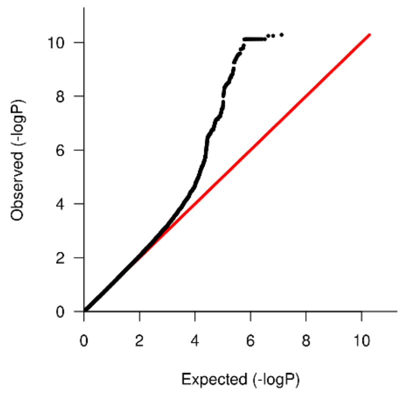

λ = 0.990

Fixed effect: top SNP from GWAS withers height (rs68603062)

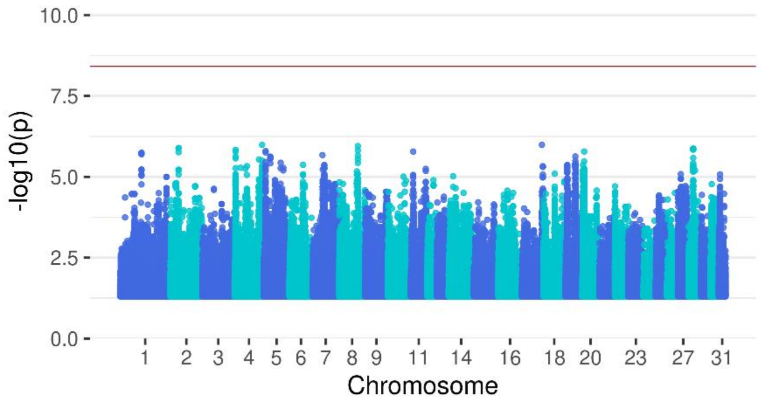

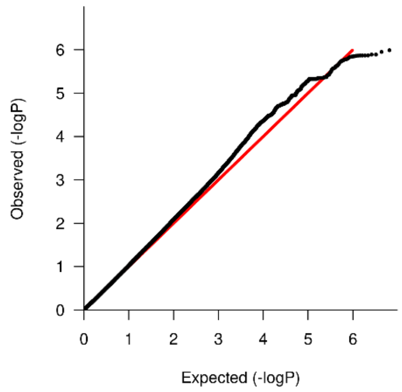

λ = 1.016

Fixed effect: own top SNP from GWAS (rs1148343189)

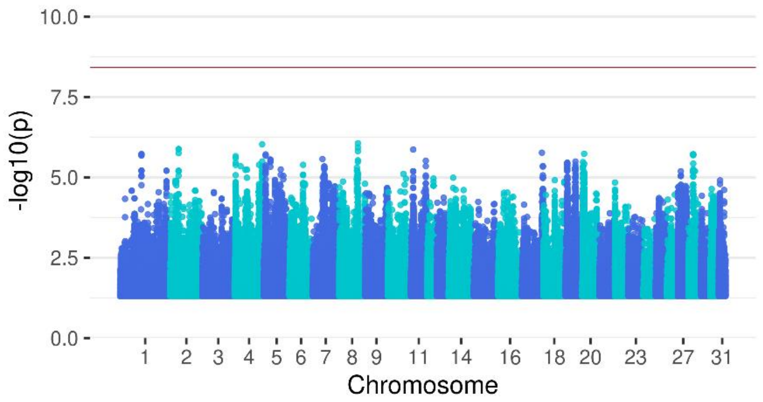

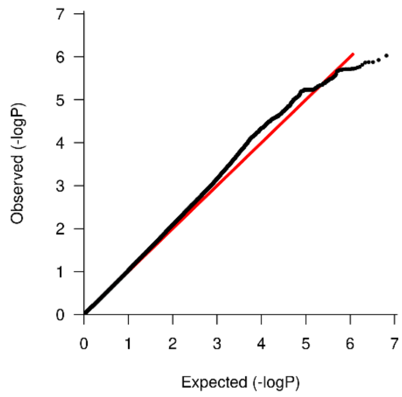

λ = 1.019

<u>Shape of feet (hoof size)</u> <u>[narrow, small - wide, big]</u>

Fixed effect: withers height

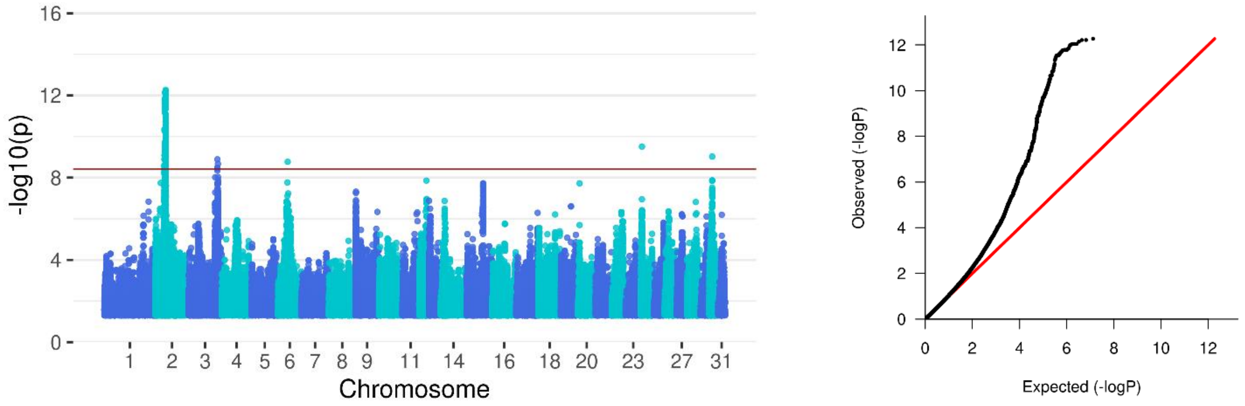

λ = 0.984

Fixed effect: top SNP from GWAS withers height (rs68603062)

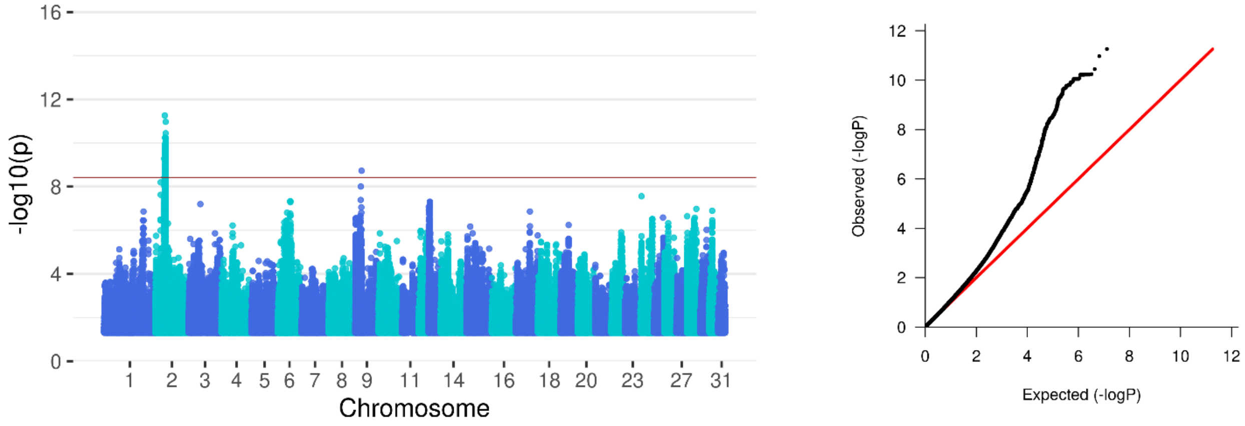

λ = 1.063

Fixed effect: own top SNP from GWAS (rs1137427665)

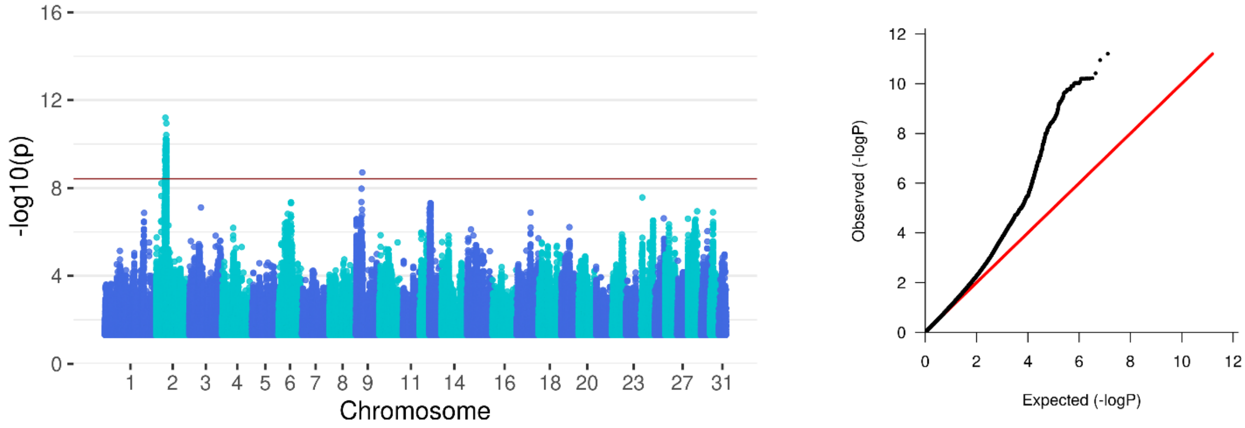

λ = 1.064

<u>Rotation in the hock</u> <u>[marked rotation]</u>

Fixed effect: withers height

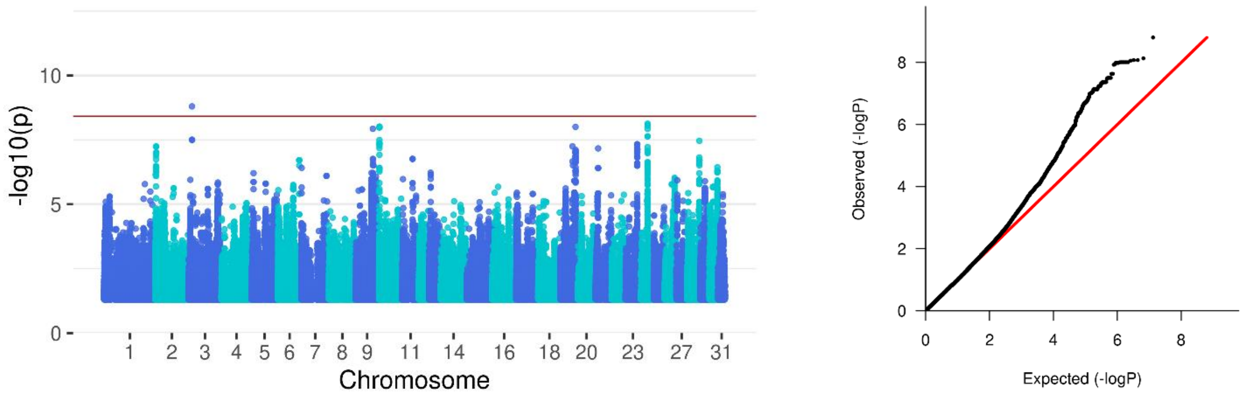

λ = 0.996

Fixed effect: top SNP from GWAS withers height (rs68603062)

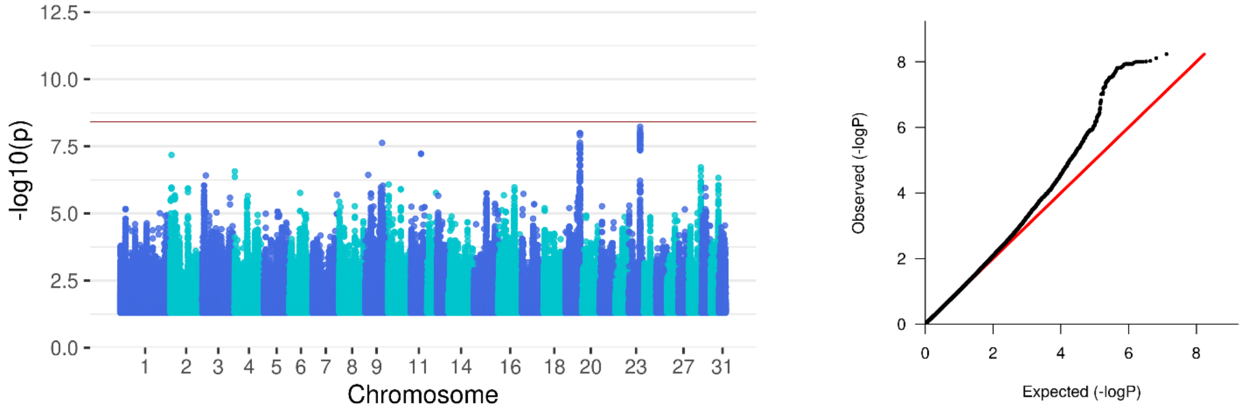

λ = 1.016

Fixed effect: own top SNP from GWAS (ECA3: 107,211,701 bp)

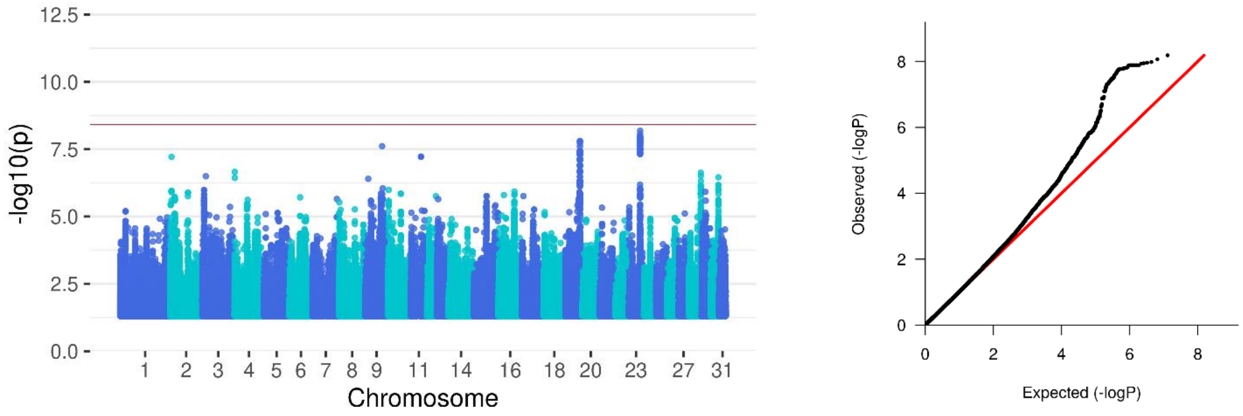

λ = 1.014
